# Human in vitro airway models to assess point-of-contact respiratory toxicity of surfactants and derive human equivalent concentrations after single dose exposure

**DOI:** 10.1093/toxsci/kfag118

**Published:** 2026-09-10

**Authors:** Andreas O Stucki, Monita Sharma, Jessica R Murray, Sandra Verstraelen, Evelien Frijns, Karen Hollanders, An Jacobs, Anthony Luz, Sylvie Remy, Nuria Roldan, Jo Van Laer, Miyoung Yoon, Keith Salazar, Natalia Garcia-Reyero Vinas, Anna B Lowit, Amy J Clippinger

**Affiliations:** PETA Science Consortium International e.V., Stuttgart 70499, Germany; PETA Science Consortium International e.V., Stuttgart 70499, Germany; Office of Chemical Safety and Pollution Prevention, U.S. Environmental Protection Agency, Research Triangle Park, NC 27711, United States; Environmental Intelligence Unit, Flemish Institute for Technological Research (VITO), Mol BE-2400, Belgium; Environmental Intelligence Unit, Flemish Institute for Technological Research (VITO), Mol BE-2400, Belgium; Environmental Intelligence Unit, Flemish Institute for Technological Research (VITO), Mol BE-2400, Belgium; Environmental Intelligence Unit, Flemish Institute for Technological Research (VITO), Mol BE-2400, Belgium; Office of Chemical Safety and Pollution Prevention, U.S. Environmental Protection Agency, Research Triangle Park, NC 27711, United States; Environmental Intelligence Unit, Flemish Institute for Technological Research (VITO), Mol BE-2400, Belgium; PETA Science Consortium International e.V., Stuttgart 70499, Germany; Environmental Intelligence Unit, Flemish Institute for Technological Research (VITO), Mol BE-2400, Belgium; Office of Chemical Safety and Pollution Prevention, U.S. Environmental Protection Agency, Washington, DC 20460, United States; Office of Chemical Safety and Pollution Prevention, U.S. Environmental Protection Agency, Washington, DC 20460, United States; Office of Chemical Safety and Pollution Prevention, U.S. Environmental Protection Agency, Washington, DC 20460, United States; Office of Chemical Safety and Pollution Prevention, U.S. Environmental Protection Agency, Washington, DC 20460, United States; PETA Science Consortium International e.V., Stuttgart 70499, Germany

**Keywords:** new approach methodologies (NAMs), nonanimal methods, inhalation toxicity, respiratory tissue irritation, in vitro, surfactants

## Abstract

Surfactants are widely used in products such as soaps, adhesives, and agrochemical formulations where they function as emulsifiers, wetting agents, detergents, foaming agents, or dispersants. Despite the potential for inhalation exposure, human-relevant hazard data are limited. Surfactants can interact with amphiphilic cell membranes, compromising membrane integrity and leading to cytotoxicity. To assess point-of-contact respiratory tissue irritation potential of these chemicals, a human bronchial epithelial cell line (BEAS-2B) and a reconstructed human respiratory epithelial (RHRE; MucilAir) model were exposed to two surfactants—oleoyl sarcosine or Triton X-100 as aerosols (BEAS-2B cells only) or liquids (in both systems). Aerosol exposures were not further pursued due to similar trends in results as liquid exposures and the need for further technical optimization. Cells were exposed for 24 h to 30 µl (91 µl/cm²) of either surfactant at five concentrations. Cellular effects were assessed after 24 h of exposure in BEAS-2B cells and MucilAir, and at 7 d after the start of exposure in MucilAir only. Generally, dose-dependent effects were observed for both surfactants within each cell system. Oleoyl sarcosine was more toxic in BEAS-2B cells compared with Triton X-100, and no clear difference in toxicity between the two surfactants was observed in MucilAir. Benchmark Dose (BMD) and multiple-path particle dosimetry (MPPD) modeling were performed to translate in vitro data to human equivalent concentrations (HECs). This manuscript demonstrates the application of an in vitro test method and in vitro to in vivo extrapolation (IVIVE) to predict point-of-contact respiratory tissue irritation in humans.

Regulators require data on the biological effects of inhaled chemicals, traditionally obtained from rat studies (OECD 2018). However, differences in anatomy and physiology between rat and human respiratory tracts limit the precision with which the rat test predicts the human response (Stucki et al. 2024). Therefore, efforts have focused on optimizing in vitro testing approaches that can more effectively and efficiently predict the risk of inhaled substances to humans (Jeong et al. 2019, 2021; McGee Hargrove et al. 2021; Sadekar et al. 2025; Choe et al. 2026).

The work presented here builds upon the 2016 workshop “Alternative Approaches for Acute Inhalation Toxicity Testing to Address Global Regulatory and Non-Regulatory Data Requirements” and a testing approach previously developed and used for assessing silane vapors (Clippinger et al. 2018a, 2018b; Sharma et al. 2023). Expanding on the previous study design, the present study evaluated the same two biological systems for their ability to assess local irritation hazards of surfactants and demonstrated the use of liquid application as a suitable mode of exposure for the test chemicals.

Surfactants are used in various products (e.g. soaps, adhesives, and agrochemical formulations) as emulsifiers, wetting agents, detergents, foaming agents, or dispersants. Surfactants are “surface-active agents” that alter the surface tension of liquids and form micelles in solutions above their critical micelle concentration (CMC). Exposure to surfactants can occur during manufacturing, occupational settings (e.g. commercial cleaning or herbicide application), and consumer use via various routes, including inhalation of aerosolized products. Due to their extensive use (global consumption of 17.6 million metric tons in 2023; S&P Global 2024), surfactants are of interest to regulatory agencies (Lanigan 2001; Perinelli et al. 2016; Seweryn 2018; Okasaka et al. 2019).

Despite the widespread use of surfactants and the potential for inhalation, respiratory toxicity data for this chemical class remain limited. Oleoyl sarcosine is an anionic surfactant and a member of the class of alkyl sarcosines, and polyethylene glycol p-(1,1,3,3-tetramethylbutyl)phenyl ether (better known by its trade name, Triton X-100) is a nonionic surfactant and a member of the class of alkylphenol ethoxylates. These two chemicals were selected for in vitro evaluation because prior inhalation studies in rats identified portal-of-entry toxicity and local inflammation, but did not report a no observed adverse effect concentration (NOAEC), increasing uncertainty (US EPA 2024). Assessing these two chemicals will determine whether in vitro toxicity assays can reliably identify known irritants, build confidence in their application for data-poor surfactants, and improve human relevance of available data that can be used to estimate inhalation hazards of surfactants.

A human bronchial epithelial cell line (BEAS-2B) and a reconstructed human respiratory epithelium (RHRE, MucilAir) were used to evaluate the respiratory tissue irritation potential of both surfactants. BEAS-2B cells were exposed to oleoyl sarcosine and Triton X-100 as liquids or aerosols, whereas MucilAir tissues were exposed via liquid only (as described in the Results section). Liquid exposure was selected based on previous studies, where chemicals were delivered to cells via direct pipetting as it allows for precise dose control, does not require special equipment and expertise, and is often considered as worst-case scenario (Jackson et al. 2018; Jeong et al. 2019, 2021; McGee Hargrove et al. 2021; Welch et al. 2021; OECD 2022; Ruijter et al. 2025; Choe et al. 2026). A suite of complementary cellular endpoints indicative of respiratory point-of-contact irritation was assessed 24 h and 7 d (MucilAir only) after exposure.

To render the generated results useful for hazard assessment, a fit-for-purpose driven in vitro to in vivo extrapolation (IVIVE) workflow was applied. To this end, benchmark dose (BMD) modelling was combined with multi-path particle dosimetry (MPPD) modelling to derive human equivalent concentrations (HEC). Translating in vitro mass per surface area (µg/cm^2^) into external concentrations (mg/m^3^) is necessary to characterize hazardous exposure concentrations. MPPD is a mechanistic dosimetry model that applies mathematical deposition efficiencies for diffusion, sedimentation, and impaction to predict deposition and clearance of aerosols within the human respiratory tract (Miller et al. 2016). Particle size and human activity patterns strongly influence regional deposition patterns within the human respiratory tract and must be considered during inhalation dosimetry modeling. Therefore, EPA MPPD v2.0 model simulations were tailored to accommodate the physicochemical properties of oleoyl sarcosine and Triton X-100 across a range of reported aerosol sizes under expected occupational and consumer inhalation exposure scenarios. This approach reduces uncertainty by leveraging human-relevant cell systems and mechanistic inhalation dosimetry, replacing the default interspecies assumptions and scaling factors otherwise required to extrapolate animal-derived point of departures (PODs).

This study thereby aimed to demonstrate the ability of two in vitro systems to reliably generate data on the point-of-contact respiratory irritation effects of surfactants, establish scientific confidence in the use of liquid exposure to these systems, and show how to translate the in vitro results to HEC values that can inform hazard assessments. This work shows for the first time how in vitro and in silico approaches can be combined to assess surfactants for their potential to cause respiratory tissue irritation without the use of animals.

## Materials and methods

All materials used in this study, including manufacturer information, can be found in Table S1.

### Experimental design

**Fig. 1.**
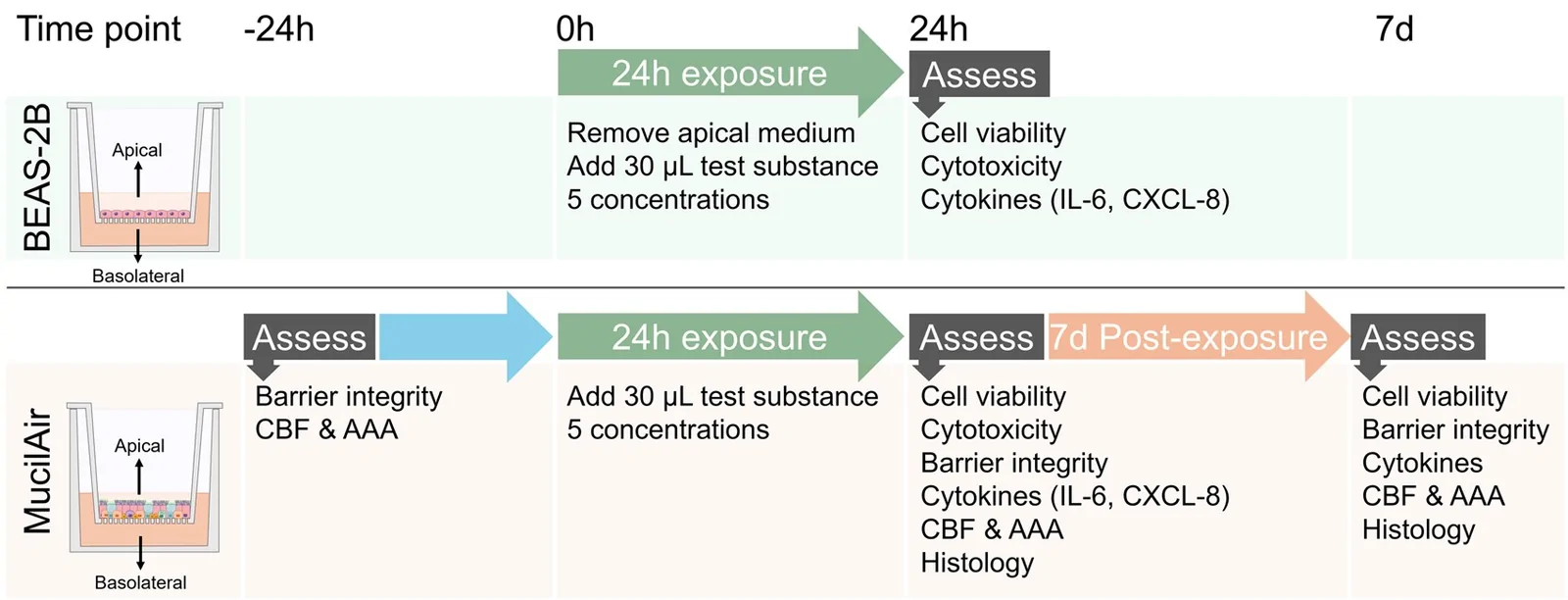
Study design for liquid exposure experiments, including exposure and cell collection times, cellular effects assessed, and model system used. AAA, average active (cilia) area; ALI, air–liquid interface; CBF, cilia beat frequency; IL, interleukin.

For both chemicals, five test concentrations (see Table 1) were selected based on range-finding experiments (data not shown). In the main experiments, cell viability (PrestoBlue assay) and cytotoxicity (lactate dehydrogenase (LDH) assay) were assessed for both biological systems; barrier integrity (transepithelial electrical resistance (TEER) measurements; Sharma et al. 2025) was also assessed in MucilAir (Fig. 1). Two modes of exposure (liquid and aerosol) were used to expose BEAS-2B cells, whereas MucilAir tissues were exposed to the test chemicals only via liquid exposures. Three independent experimental runs (n = 3) were conducted for each biological system and Table S2 provides the number of technical replicates (n) included for both cell models in each experimental run. For each experimental run with MucilAir, a different, individual (single) donor was used (see Table S3 for donor information).

**Table 1.** Test concentrations used in BEAS-2B cells and MucilAir for liquid exposures.

Oleoyl sarcosine						Triton X-100					
BEAS-2B			MucilAir			BEAS-2B			MucilAir		
NC (µM)	NC (µg/cm^2^)	MC (µg/cm^2^)	NC (µM)	NC (µg/cm^2^)	MC (µg/cm^2^)	NC (µM)	NC (µg/cm^2^)	MC (µg/cm^2^)	NC (µM)	NC (µg/cm^2^)	MC (µg/cm^2^)
18.7	0.6	0.5	155.6	5	5.2 ± 0.4	51.0	3	2.2 ± 0.38	85.0	5	4.2 ± 0.67
37.3	1.2	1.1 ± 0.06	311.1	10	10.3 ± 0.42	102.0	6	5.5 ± 0.76	170.1	10	9.1 ± 0.32
56.0	1.8	1.6 ± 0.10	622.3	20	20.1 ± 1.45	153.1	9	7.9 ± 0.78	340.1	20	18.6 ± 2.72
74.7	2.4	2.4 ± 0.17	1244.5	40	41.1 ± 1.38	204.1	12	13.9 ± 2.21	680.2	40	37.8 ± 2.57
93.3	3.0	3.1 ± 0.55	2489.1	80	77.8 ± 3.86	255.1	15	16.4 ± 1.18	1360.4	80	77.9 ± 4.61

Measured concentration displayed as mean ± SD. NC, nominal concentration; MC, measured concentration.

As described in a previous study (Sharma et al. 2023), controls included a solvent control (SC), an incubator control (IC), and a lysis buffer control (as a positive control for the LDH assay) for both systems. Bacterial lipopolysaccharide (LPS) was used as a positive control for proinflammatory cytokine secretion at 20 µg/ml in BEAS-2B cells and 200 µg/ml in MucilAir tissues. Cilia beating frequency (CBF) was assessed only in MucilAir, with isoproterenol hydrochloride (ISO; 50 µM) included as a positive control.

### Biological test systems

The BEAS-2B cell line, a normal human bronchial epithelial cell line, sourced from the American Type Culture Collection (ATCC), was established in 1988 by immortalizing nontumorigenic human bronchial cells with an adenovirus 12-SV40 hybrid virus. BEAS-2B cells were grown in T-75 culture flasks treated with a coating solution: 4.5 ml (0.06 ml/cm^2^) of 10 µg/ml human plasma fibronectin, 30 µg/ml VitroCol, and 10 µg/ml human serum albumin dissolved in Bronchial Epithelial Cell Basal Medium (BEBM). The Bronchial Epithelial Cell Growth Medium (BEGM) consisted of BEBM supplemented with BEGM BulletKit. Although bovine pituitary extract is a component of the BEGM BulletKit, it was not used in this study as it has been shown to be unnecessary to culture BEAS-2B cells (Sharma et al. 2023). The cells were kept in an incubator at 37°C and 5% carbon dioxide (CO_2_). Before reaching 80% confluence, the cells were subcultured using 1x TrypLE Express Enzyme. The medium was refreshed every 2 to 3 d and cells were subcultured every 4 to 5 d (1,500 to 3,000 cells/cm^2^). Cells were used until passage 20.

Prior to exposure, 24-well PET clear inserts with pore size of 0.4 µm (0.33 cm^2^ surface area) were coated with the coating solution described above. The inserts were placed in a sterile 24-well plate, and 600 µl BEGM was added to the basolateral compartment. BEAS-2B cells suspended in BEGM (1.2 × 10^5^ cells/ml) were seeded apically at a volume of 200 µl (resulting in ∼72,725 cells/cm^2^), and allowed to attach and grow for 72 h. The cells were airlifted right before exposure by removing the apical medium and adding 600 µl of fresh medium basally.

MucilAir is a fully differentiated, commercially available RHRE model grown on cell inserts that consists of primary human bronchial epithelial cells isolated from biopsies (Huang et al. 2009). For this study, individual (single-donor) tissues were used from three randomly selected normal, nonsmoking donors (see Table S3 for donor information provided by the supplier). Upon receipt, the MucilAir tissues were placed in an incubator (37°C, 5% CO_2_) for 1 h after which they were removed from the nutritive gel (proprietary) used during transport and transferred to a new 24-well plate filled with 700 µl prewarmed MucilAir Medium at 37°C. The plate was transferred to the incubator for culturing (37°C, 5% CO_2_). After 48 to 72 h, the basal medium was changed (700 µl), and each MucilAir insert was washed apically with MucilAir Medium to remove mucus. For this, 200 µl of 37°C warm medium was added for 10 to 20 min on the apical surface of the inserts and incubated at 37°C. After incubation, 100 µl of apical medium was gently pipetted up and down 3 times to loosen the mucus from the cells’ surface. All apical medium was gently removed by aspiration. After an additional 72 h (i.e. 24 h before exposure), CBF and cilia average active area (AAA) were measured before mucus washing. After washing, TEER was measured. Subsequently, basal medium (700 µl) was replaced and inserts were placed in the incubator for another 24 h until exposure.

### Preparation of test chemicals

The two chemicals tested in this study are oleoyl sarcosine and Triton X-100 (see Table 2). The chemicals were stored at room temperature for the duration of this study. All stocks and dilutions were prepared in sterilized glass vials. Due to the viscosity of the surfactants, all stock solutions were prepared using a positive displacement pipette. BEBM was used for BEAS-2B dilutions, and saline solution (0.9% sodium chloride (NaCl), 1.25 mM calcium chloride (CaCl_2_), and 10 mM HEPES) was used for MucilAir if not otherwise described.

**Table 2.** Properties of oleoyl sarcosine and Triton X-100.

Oleoyl sarcosine	Triton X-100 (polyethylene glycol p-(1,1,3,3-tetramethylbutyl)phenyl ether)
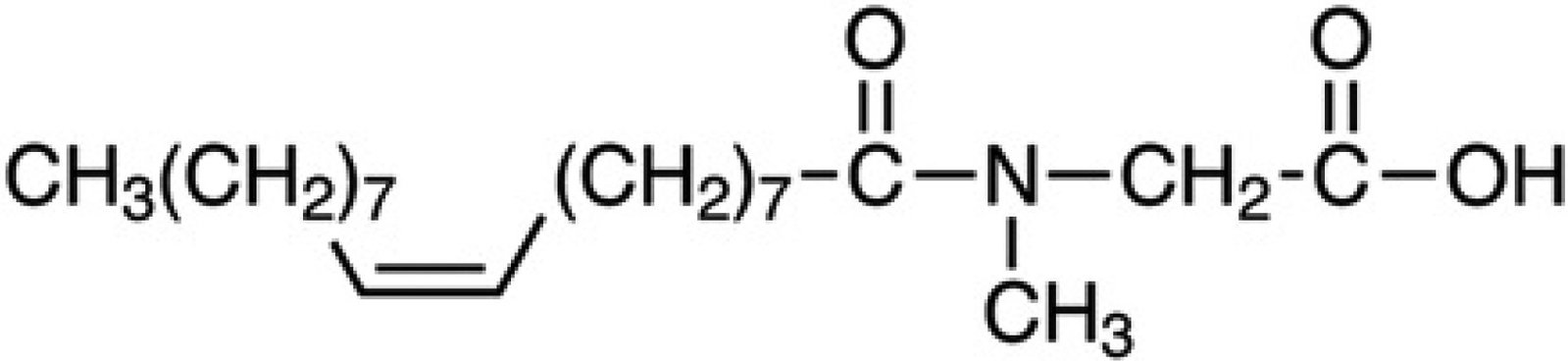	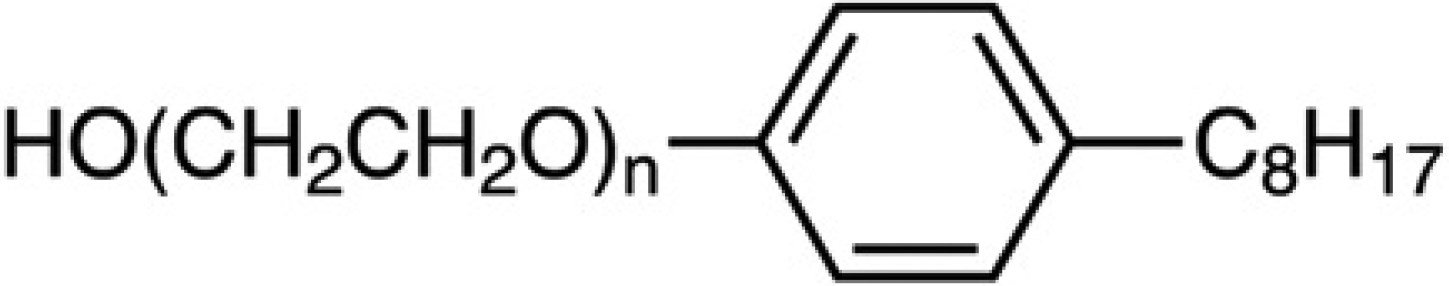
CAS number: 110-25-8	CAS number: 9036-19-5
Anionic surfactant; negatively charged	Nonionic surfactant; not charged
Insoluble in water	Soluble in water
Computed logP value (XLogP3-AA): 6.8	Computed logP value (XLogP3-AA): 4.6
Molecular weight: 353.5 g/mol	Molecular weight: 646.85 g/mol (n = 10)
Density: 0.961 g/ml	Density: 1.07 g/ml
Critical micelle concentration: 0.026 g/l (74 µM)	Critical micelle concentration: 0.17 g/l (260 µM)
GHS hazard (inhalation): Acute Tox Cat. 4; STOT SE 3	GHS hazard (inhalation): STOT SE 3
In vivo: Skin irritation 2; eye dam. 1 In vitro: Skin irritation 2; eye dam. 2 (see supplementary file)	In vivo: Skin irritation 2; eye dam. 1 In vitro: Skin irritation 2; eye dam. 1 (see supplementary file)

GHS, globally harmonized system; STOT SE, specific target organ toxicity–single exposure.

Oleoyl sarcosine stocks were prepared at two different concentrations in 100% dimethyl sulfoxide (DMSO). For BEAS-2B cells and MucilAir, stock solutions of 186.68 mM (66 mg/ml) and 497.8 mM (176 mg/ml) were prepared, respectively. For both cell systems, serial dilutions were prepared in 100% DMSO at 200x the intended final exposure concentration. Immediately before dosing, each dilution was diluted 1:200 into BEBM (BEAS-2B) or saline (MucilAir) to generate the 1x working solutions used for exposure (final DMSO: 0.05%). 0.05% DMSO in the corresponding vehicle, BEBM (for BEAS-2B) or saline solution (MucilAir), was therefore used as solvent controls (SC) for oleoyl sarcosine exposures.

For BEAS-2B and MucilAir, a stock of 0.255 mM (0.165 mg/ml) and 1.36 mM (0.879 mg/ml) of Triton X-100 were prepared, respectively. From the stocks, serial dilutions were prepared in BEBM (for BEAS-2B) or saline (MucilAir) and mixed by vortexing. Finally, BEBM (for BEAS-2B) or saline solution (MucilAir) were used as SC for Triton X-100 exposures.

### Analysis of test chemicals

A single 100 µl sample of all dilutions was taken to quantify the test chemical concentration. These samples were diluted 10 times in ultrapure water/methanol and vortexed before analysis. The dilutions were transferred to a liquid chromatography vial, and 2 µl were used for injection. Each sample was analyzed in duplicates.

The instrumental analysis was performed by Ultra Performance Liquid Chromatography (UPLC)-tandem mass spectrometer using a Waters H-class Acquity UPLC system. Triton X-100 was separated on an Acquity UPLC BEH C18 column (100 mm × 2.1 mm; 1.7 µm) at 50°C. The mobile phase consisted of ultrapure water (solvent A) and methanol/acetonitrile with 2 mM ammonium acetate (1/1 v/v; solvent B), and both solvents A and B were acidified with 0.1% acetic acid. The gradient was programmed from 10% to 100% of solvent B (v/v) in 6 min at a 0.4 ml/min flow rate. For chemical identification, the UPLC system was coupled to a Waters Xevo tandem mass spectrometer, which was operated in the positive electrospray ionization mode (ESI+).

Oleoyl sarcosine was identified using multiple reaction monitoring mode (MRM) and by its characteristic retention time (LC). Triton X-100 was identified using a selected ion recording (SIR) mode and its LC retention time. For both chemicals, quantification was conducted with the external calibration method. The limit of quantification (LOQ) was 0.25 µg/ml for both compounds. Samples were analyzed in batches of 10. Every analysis batch contained the necessary quality control samples, including procedure blank, spiked water sample, spiked sample (trueness), and sample duplicate analysis (intraday precision). Nominal and measured concentrations of the test substances are provided in Table S4.

### Exposure of BEAS-2B cells to test chemicals

Immediately before exposure, cells were checked for confluency using a conventional inverted microscope. After that, the BEGM was completely removed from the apical and basolateral sides of all the inserts, and 600 µl of fresh BEBM (without growth factors to stop the cells from growing further) was added basolaterally.

For liquid exposure, 30 µl of test solution (91 µl/cm^2^) was pipetted on the apical surface of the cells and the tissues were then placed in the incubator for 24 h. Details for aerosol exposure are reported in the supplementary document.

After 24 h of exposure in the incubator, 200 µl basolateral medium was collected for assessment of cytotoxicity. The remaining volume was frozen and stored at −80°C for later assessment of cytokine secretion after the inserts were transferred to a new, sterile 24-well plate prefilled with warm 600 µl fresh BEBM.

Three biologically independent experimental runs, with three replicates per treatment, using different cell passages, were performed for each exposure condition.

### Exposure of bronchial MucilAir tissues to test chemicals

Twenty-four hours before exposure, CBF, AAA, and TEER were measured for quality control purposes and mucus was removed. Immediately before exposure, each tissue insert was inspected under a conventional inverted microscope to ensure the quality of the epithelia. Subsequently, tissues were exposed by pipetting 30 µl of test solution (91 µl/cm^2^) on the apical surface of MucilAir and then placed in the incubator for 24 h.

After 24 h of exposure, 200 µl basolateral medium was collected for cytotoxicity assessment. The remaining medium was stored at −80°C for later assessment of cytokine secretion. The inserts were then transferred to a new, sterile 24-well plate prefilled with 700 µl MucilAir medium. Next, CBF and AAA were measured before the inserts were washed apically three times with 37°C warm saline solution. TEER was measured after apical washing as described below. Out of four tissues, two tissues were used for PrestoBlue assay and afterwards fixed for histology.

The other two remaining tissues were kept for an additional 6 d (until 7 d postexposure). Medium was changed at days 3 and 6 postexposure and inserts were apically washed with saline solution to remove mucus. At 7 d postexposure, basolateral medium was collected and stored at −80°C for cytokine measurements. CBF, AAA, and TEER were again measured. The tissues were then used for PrestoBlue assay and afterwards fixed for histology.

Three biologically independent runs, using a different single donor in each run (see Table S3 for donor information), were performed for each exposure condition. Each run included 4 replicates of tissues from a single donor per treatment group.

### Mitochondrial activity to determine cell viability

Cell viability was assessed using the PrestoBlue Cell Viability Reagent according to the manufacturer’s instructions. The PrestoBlue assay measures cellular metabolic activity by detecting the reduction of the resazurin in the PrestoBlue reagent to resorufin. Viable cells reduce the PrestoBlue reagent, turning it red and becoming highly fluorescent, which can be measured using a spectrophotometer.

In this study, cell viability was determined after 24 h exposure and at 7 d postexposure (MucilAir only). Briefly, after removing the basolateral medium, 200 µl of a 10% v/v PrestoBlue solution in BEGM (for BEAS-2B) or in MucilAir Medium (MucilAir) was added on the apical side of the inserts. After 1 h in the incubator (at 37°C, 5% CO_2_) in the dark, 2 samples/well (90 µl each) were taken from the apical solution of each insert, transferred to a 96-well plate, and fluorescence was measured using a multi-mode microplate reader in fluorescence mode (excitation: 552-22 nm, emission: 590-20 nm). 10% v/v PrestoBlue diluted in the corresponding cell culture media was also incubated for 1 h at 37°C in the dark and used as a blank.

The averaged value of the blank was subtracted from all PrestoBlue measurements and cell viability was expressed as the percentage of relative fluorescence intensity (RFI) of treated cells relative to the RFI of the cells exposed to SC (SC equals 100% cell viability; see equation below). Samples from each experimental run were compared with their SC of the same experimental run. Significant changes in % cell viability compared with SC were assessed by mixed models considering experiment ID (biological replicate, i.e. donor ID) as random factor. The R-packages for mixed model analyses “lme4” (Bates et al. 2015) and “lmerTest” (Kuznetsova et al. 2017) were applied. *P*-value less than 0.05 was used as cut-off for statistical significance.


$$\mathrm{Cell}\;\mathrm{viability}\;(\%)=100\times \frac{\left({\mathrm{RFI}\;}_{\mathrm{Sample}}-{\mathrm{RFI}\;}_{\mathrm{blank}}\right)}{\left({\mathrm{RFI}\;}_{\mathrm{Solvent}\;\mathrm{control}}-{\mathrm{RFI}\;}_{\mathrm{blank}}\right)}$$

### Cellular membrane integrity using LDH assay to determine cytotoxicity

Cytotoxicity was assessed in both biological systems using the LDH Assay Kit-WST per manufacturer’s instructions (Dojindo). Briefly, after 24 h of exposure, 2 × 100 µl of the basolateral medium (two technical replicates) were transferred to a 96-well plate and used for the LDH assay. Basolateral samples from the insert(s) treated with the positive control (lysis solution included in the LDH assay kit; 50 µl incubated for 18 h (BEAS-2B) or 3 h (MucilAir)) and blanks (BEBM for BEAS-2B and MucilAir Medium) were also transferred to the 96-well plate. Subsequently, 100 µl LDH substrate mix were added to each sample well and incubated for 30 min at room temperature in the dark. The reaction was stopped by the addition of 50 µl stop solution. Optical density (O.D.) measurements were conducted using a multi-mode microplate reader (wavelength: 490 nm).

The averaged O.D. value of the blanks was subtracted from all measurements, and fold change in cytotoxicity levels relative to SC exposure was calculated using the following equation:


$$\mathrm{LDH}\;(\mathrm{fold}\;\mathrm{change})=\frac{\left({O.D.}_{\mathrm{Sample}}-{O.D.}_{\mathrm{blank}}\right)}{\left({O.D.}_{\mathrm{Solvent}\;\mathrm{control}}-{O.D.}_{\mathrm{blank}}\right)}.$$

Samples from each experimental run were compared with their SC of the same experimental run. Statistical analysis was assessed by mixed models upon logarithmic transformation (ln) while considering experiment ID (biological replicate, i.e. donor ID) as random factor. R-packages for mixed model analyses “lme4” and “lmerTest” were applied. *P*-value less than 0.05 was used as cut-off for statistical significance.

### Proinflammatory markers

To measure proinflammatory markers, interleukin (IL)-6 and CXCL-8 (also known as IL-8), the Meso Scale Discovery (MSD) V-PLEX Assay was conducted following the manufacturer’s protocol. Samples corresponding to tissues with viability lower than 10% were excluded from the analysis. The assay uses an electrochemiluminescent detection method and is a highly sensitive multiplex enzyme-linked immunosorbent assay (ELISA). Calibration curves were employed to determine the cytokine concentrations, which are expressed in pg/ml. After 24 h of exposure or at 7 d postexposure (MucilAir only), basolateral medium was stored at −80°C until analysis using the MSD assay.

A positive control insert for inflammation was prepared in each experimental run for both the 24 h and the 7 d time points. LPS was always administered as a liquid exposure. Briefly, BEAS-2B cells were exposed to 20 µg/ml and MucilAir tissues were exposed to 200 µg/ml apically for 24 h. The basolateral medium was collected 19 to 24 h postexposure (for the aerosol experiments) and directly after liquid exposures.

The precoated MSD plate was washed three times. Calibrators and samples were diluted 4-fold (standard curve) and 10-fold (samples) in assay diluent, then added. The plate was sealed and incubated overnight at 4°C. After another three washes, the detection antibody solution was added to each well. The plate was incubated at room temperature for 2 h on an orbital shaker, followed by another three washes. Finally, the read buffer was added, and the plate was analyzed on the MSD instrument.

For both cytokines, the MSD software calculated the protein concentrations in each sample based on the calibrators that were incubated alongside the samples and applying a weighted, four-parameter logistic fit per default. Reported concentrations were then processed further using R (Bunn 2008). Concentrations below the lower limit of detection (LLOD) were removed. Significant changes in protein concentration were analyzed relative to SC and were assessed by mixed models upon logarithmic transformation (ln) while considering experiment ID (biological replicate, i.e. donor ID) as random factor. R-packages for mixed model analyses “lme4” (Bates et al. 2015) and “lmerTest” (Kuznetsova et al. 2017) were applied. *P*-value less than 0.05 was used as cut-off for statistical significance.

### Barrier integrity (TEER, MucilAir only)

The template from the recent publication on reporting recommendations was used to provide the details of the TEER assay (Sharma et al. 2025). For MucilAir, the EVOM Manual voltohmmeter was used with STX4 EVOM electrode. The voltohmmeter was calibrated using the 99673-test resistor and the electrodes were calibrated using KCl (40 mM, 80 mM, and 160 mM) solutions at room temperature. The electrode was washed with 70% ethanol before use and wiped. For measurement, MucilAir Medium was used as electrolyte solution and equilibrated at 37°C for 30 min. The electrodes were equilibrated in MucilAir Medium for 5 min before measurement. The MucilAir tissues were removed from the incubator or, if measured after exposure, they were processed for TEER measurement after CBF/AAA measurement and apical washing steps (see section: Exposure of bronchial MucilAir tissues to test chemicals). The medium was exchanged (200 µl MucilAir Medium apical and 700 µl basolateral) and inserts were placed back in the incubator for 5 min. After the incubation time, individual well plates containing up to four inserts were transferred to the laminar flow hood, a timer was set to record the duration for which the test system was out of the incubator (2 min on average per plate), and the TEER was measured. The electrodes were rinsed with MucilAir Medium between measurements for each experimental group. After measurement, the electrolyte solution was removed via pipetting, ALI was restored apically, and 700 µl fresh MucilAir Medium was added to the basolateral side of the insert before putting it back in the incubator. The inserts were outside the incubator for 3 to 4 min. Resistance of the cells (R_cells_) was calculated by subtracting blank resistance (R_blank_; 100 Ωcm^2^) from total measured resistance (R_total_).


$$R_{\mathrm{cells}\;}(Ω)=R_{\mathrm{total}\;}(Ω)-R_{\mathrm{blank}}\;(Ω)$$

TEER was then calculated by multiplying R_cells_ by the cell culture insert surface area (SA; 0.33 cm^2^).


$$\mathrm{TEER}\;(\Omega \cdot {\mathrm{cm}}^{2})=R_{\mathrm{cells}}\;(Ω)\times \mathrm{SA}\;({\mathrm{cm}}^{2})$$

Significant changes in TEER compared with the SC for a particular time point (pre-exposure, immediately after 24 h exposure, or 7 d postexposure) were assessed by mixed models while considering experiment ID (biological replicate, i.e. donor ID) as random factor. R-packages for mixed model analyses “lme4” (Bates et al. 2015) and “lmerTest” (Kuznetsova et al. 2017) were applied. *P*-value less than 0.05 was used as cut-off for statistical significance.

### Cilia beating frequency and average active area (MucilAir only)

On each MucilAir insert, two microscopic fields were measured with a 10x objective on a Zeiss Axiovert microscope (Zeiss, Jena, Germany) equipped with a heated stage-top incubator system (Chamlide WP-S-10F with air mixer CU-501, Microsystem, Sweden) and a Basler acA1300-200 µm USB3 video camera. A 5-s video, 100 frames per second, 512 frames was taken and analyzed with the CiliaX software. All measurements were recorded at 37°C. Results are provided as percentage average active area (AAA; %) and cilia beating frequency (CBF; Hz) within active areas only (>3.5 Hz). A data handling error caused data loss, resulting in a single technical replicate (n = 1) for the 5 µg/cm^2^ Triton X-100-exposed tissues of Donor 2.

A positive control insert for CBF was prepared in each experimental run for the 24 h and 7 d time points. Isoproterenol (ISO) was always administered as a liquid exposure. Briefly, MucilAir tissues were exposed to 50 µM ISO apically for 2 h before CBF was measured. ISO is known to increase the CBF (Behrsing et al. 2022). Unfortunately, due to the same data handling error, the ISO control for donor 2 at 24 h is also lost.

### Histology (MucilAir only)

For MucilAir, two inserts/treatment were processed for histology. After conducting cell viability measurements, the inserts were washed 3 times for 5 min in PBS+ (PBS Ca^+2^, Mg^+2^) and fixed for 30 min at room temperature using 4% formaldehyde solution (0.4 ml apical and 0.4 ml basolateral). After fixation, the inserts were washed 3 times for 5 min each in PBS+ in a new 24-well plate. After washing, the inserts were transferred to a 25 ml tube filled with 25 ml PBS+ at 4°C and shipped to Cerba Research (France) for processing. After embedding, the samples were sectioned at 3 µm ±1 thickness and dried for at least 2 h in a ventilated oven at 45°C (±5°C) before proceeding to staining (Alcian Blue/Periodic Acid Schiff/Hematoxylin). The slides were digitized using the NANOZOOMER-S360 scanner in bright field mode with a 20x objective. No z-stacks were acquired.

### BMD modelling

BMD analysis was performed on TEER, viability (resazurin metabolism), cytotoxicity (LDH release), IL-6 and CXCL-8 secretion, ciliary beat frequency (CBF), and average active cilia area (AAA) using BMDS Online (version 25.1) developed by the EPA (US EPA 2025a). A benchmark response of one control SD (BMR = 1SD) from the control mean (BMD_1SD_) was utilized. Fits were performed with normal constant and nonconstant variance versions of a linear (unrestricted), power (restricted), polynomial degree 2 to 6 (restricted), exponential 3 and 5 (restricted), and Hill (restricted) models using default settings for continuous models. Lower 95% confidence intervals of the BMD (BMDL_1SD_) were also calculated and reported. For BMD analysis, technical replicates were averaged (n = 2 to 4 per MucilAir donor or BEAS-2B exposure group) and the mean and SD were entered as datapoints into BMDS. Both absolute and normalized values (% or fold-change normalized to vehicle controls) were analyzed. In most cases, data normalization improved BMD model fits and the reported BMD_1SD_ and BMDL_1SD_ values are based on normalized data. In cases where absolute values improved BMD model fits (e.g. 7 d TEER following Triton X-100 exposure), they were used to derive BMDL values.

The BMD modeling approach followed the U.S. EPA Benchmark Dose Technical Guidance (US EPA 2012) and was applied using a priori criteria to identify statistically adequate and biologically plausible models. When BMDS identified a recommended model with an acceptable fit, that model was retained as the primary result. In the absence of a BMDS recommendation, the best model was selected based on consideration of *P*-value (goodness of fit, only *P*-values >0.1 considered), model with lowest Akaike’s Information Criterion (AIC) value from models with viable fits, biological plausibility and stability of BMDL predictions, visual fit, and the absolute value of scaled residuals near the control and BMD. Due to the biphasic nature of cell viability measurements in MucilAir, the data were deemed unsuitable for BMD modeling and so the no observed adverse effect concentration (NOAEC) and lowest observed adverse effect level (LOAEC) were reported. The detailed BMD results are provided in the supplementary document.

### MPPD modelling for IVIVE

The MPPD model developed by Applied Research Associates, Inc (ARA, Raleigh, NC) in collaboration with the Dutch National Institute of Public Health and the Environment (RIVM) is a mechanistic dosimetry model that calculates regional deposition of particles throughout the respiratory tract (Asgharian and Anjilvel 1998; Asgharian et al. 2001; Miller et al. 2016). The EPA MPPD model (EPA MPPD v.2.0) includes deposition, clearance, and retention predictions for multiple particle types, including nonhygroscopic, solid hygroscopic, and liquid hygroscopic (US EPA 2026). The EPA MPPD v2.0 model was applied to extrapolate in vitro BMDLs for oleoyl sarcosine and Triton X-100 to human equivalent concentrations (HECs).

MPPD simulations must be customized for each chemical to ensure that relevant particle sizes and human ventilation rates are accurately applied. An AI-assisted search (using Microsoft Copilot) was first conducted to identify the primary product types and use cases for oleoyl sarcosine and Triton X-100 that would lead to inhalation exposures. The aerosolization of metalworking fluids or use of rust inhibitor sprays will likely lead to inhalation of oleoyl sarcosine, and the use of industrial spray cleaners may lead to inhalation of Triton X-100. A literature review was then conducted to identify airborne particle size and distribution information for aerosolized metalworking fluids, rust inhibitor sprays, and industrial spray cleaners (Table 3).

**Table 3.** Predicted product types for each chemical and their reported particle size distributions.

Chemical	Product type/use case	Possible aerosol MF (%)	Potential for inhalation	Reported MMAD (µm) and GSD
Oleoyl sarcosine	Metalworking fluids	0.1–5	Very high	MMAD: 3.3–8^a^^,^^b^^,^^c^ GSD: 2.2–4.2^b^^,^^c^^,^^d^
	Rust inhibitor sprays	0.1–10	High	Reviewed similar spray device: MMAD 7.5^d^^,^^e^ GSD: 2.7^d^^,^^e^
	Lubricant additives	2–3	Unlikely	Not considered
	Cosmetic products	1–5	Unlikely	Not considered
Triton X-100	Industrial spray cleaners	1–25	High	MMAD: 1.9–3.7^f^ GSD: 2.1^f^
	Lab buffers/aerosols	0.05–2	Unlikely	Not considered
	Older pesticide formulations	1–15	Possible, but less likely	Not considered

MF, mass fraction; MMAD, mass median aerodynamic diameter; GSD, geometric SD.

References:

a Rosenthal and Yeagy (2001).

b Woskie et al. (1996).

c Wang et al. (2005).

d Sabty-Daily et al. (2005a).

e Sabty-Daily et al. (2005b).

f Lovén et al. (2019).

All exposure conditions and particle properties applied to the EPA MPPD v2.0 model are listed in Tables S5–S8. The MPPD-PNNL asymmetric geometry was applied in all cases given this model most closely predicted the measured deposition following inhalation of micron-sized particles, which is appropriate for the larger particle sizes expected for oleoyl sarcosine and Triton X-100 (Rissler et al. 2023). Occupational and consumer exposures are expected for both chemicals, and the default EPA minute ventilation parameters described in the EPA MPPD v2.0 Model User Guide were applied for each exposure scenario (US EPA 2025b): 10 m^3^/8 h for occupational exposures (1141.089 ml tidal volume, 18.2574 breaths per min) and 20 m^3^/24 h for general exposures (1000 ml tidal volume, 13.889 breaths per min). The default general inhalation rate (20 m^3^/24 h) represents an average of ICRP resting and light exercise activity values, which will be appropriate for consumer exposures (ICRP 1994). Three particle sizes were selected for each chemical to cover the range of reported particle sizes across the predicted use case scenarios (2, 3, 4 µm for Triton X-100; 3, 6, and 8 µm for oleoyl sarcosine). Deposition rather than retained mass was selected due to the high reactivity of these chemicals and the acute exposure conditions (≤ 24 h). Predicted deposited mass rates (µg/min/cm^2^) for each simulation were multiplied by the duration of the targeted exposure scenarios (8 or 24 h × 60 min/h) to obtain total deposition (µg/cm^2^). Regional HECs were then calculated for each particle size and exposure scenario using the following equation, which was previously applied in an OECD case study evaluating contact toxicity of a pesticide (OECD 2022):


$$\mathrm{HEC}(\mathrm{mg}/m^{3})=\mathrm{AC}(\mathrm{mg}/m^{3})\times \mathrm{AMF}\times \frac{{\mathrm{BMDL}}_{1SD}\;(µg/{\mathrm{cm}}^{2})}{{\mathrm{Regional}\;\mathrm{Deposition}}_{@\mathrm{AC}}\;(µg/{\mathrm{cm}}^{2})\times \mathrm{AMF}}$$

where HEC is the human equivalent concentration (mg chemical/m^3^ air), AC is the aerosol concentration (1 mg/m^3^ for these extrapolations), BMDL_1SD_ is the lower bound of the 95% confidence interval on the BMD using a BMR = 1SD, and AMF is the aerosol mass fraction. Note that the AMF cancels out and resultant HEC values are independent of the amount of active ingredient within a given product.

## Results

BEAS-2B cells were exposed to five different concentrations of the two surfactants as aerosols or as liquids (via pipetting). The two types of exposure resulted in similar trends, yet the aerosol exposure led to less pronounced cellular effects compared with liquid exposure (Figs S1 and S2). This difference between exposure modes is likely attributed to the difficulty in aerosolizing and delivering these highly viscous surfactants to the apical cell surface. Although aerosolization issues could have been mitigated through troubleshooting steps, liquid exposure was simpler and produced similar trends; therefore, the aerosol exposure conditions were not optimized in these studies. The following sections include results only from the liquid exposures, and the data from aerosol experiments have been included in the supplementary file.

### Cell health after exposure of BEAS-2B cells to surfactants

**Fig. 2.**
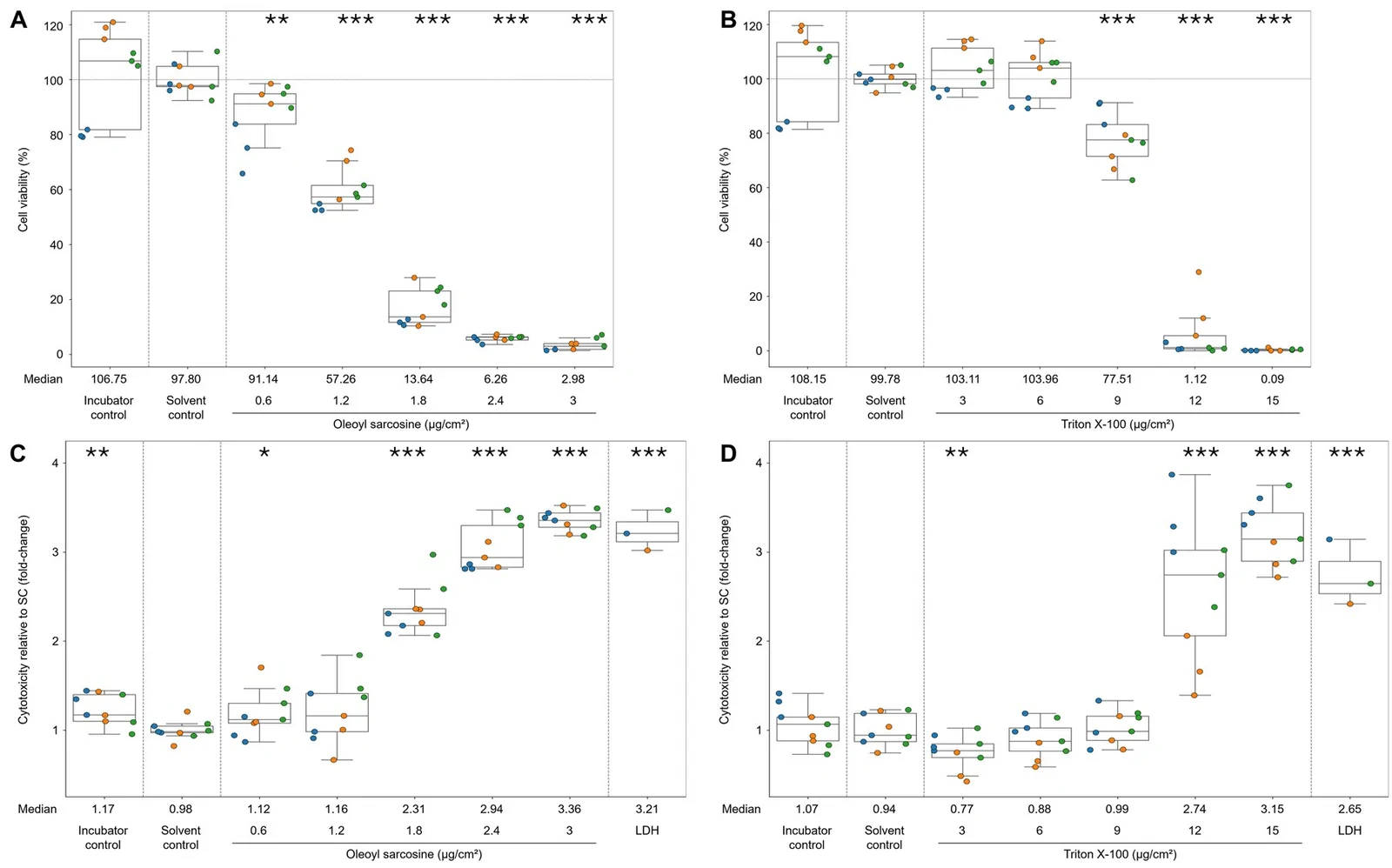
Effect of surfactant exposure on cell viability and cytotoxicity in BEAS-2B cells. BEAS-2B cells were exposed to 30 µl (91 µl/cm^2^) of five different concentrations of oleoyl sarcosine (0.6, 1.2, 1.8, 2.4, 3 µg/cm^2^), Triton X-100 (3, 6, 9, 12, 15 µg/cm^2^), their respective solvent controls (SC), or left untreated (incubator control; IC) for 24 h at the air–liquid interface. After exposure, cell viability (A and B) and cytotoxicity (C and D) were assessed. The graphs show results from three separate experimental runs (N = 3) for the two surfactants represented in different colors, with each run having three technical replicates (n = 3). The results were normalized to SC. Asterisks (*) show statistically significant differences compared with SC; *, *P *< 0.05; **, *P *< 0.01; ***, *P *< 0.001.

After 24 h of exposure, a concentration-dependent decrease in cell viability was observed for both surfactants in BEAS-2B cells (Fig. 2A and B). For oleoyl sarcosine, significant differences in cell viability relative to SC were observed for all concentrations. At the lowest tested concentration (0.6 µg/cm^2^), the decrease in cell viability was relatively mild (91.1% cell viability). Viability decreased to 57.3% for 1.2 µg/cm^2^ and below 20% for 1.8, 2.4, and 3 µg/cm^2^. For Triton X-100, no change in cell viability relative to SC was observed at the two lowest concentrations (3 and 6 µg/cm^2^), but it decreased to 77.5% at 9 µg/cm^2^ and below 10% for the two highest tested concentrations (12 and 15 µg/cm^2^). Furthermore, slightly more variability was observed in the IC than in the SC, likely because the SC was at the ALI for less time.

Similarly, a concentration-dependent increase in cytotoxicity was observed for both surfactants (Fig. 2C and D). Statistically significant increases in cytotoxicity were observed after oleoyl sarcosine exposure at the three highest concentrations, with 2.3-fold at 1.8 µg/cm^2^, 2.9-fold at 2.4 µg/cm^2^, and 3.4-fold increase at 3 µg/cm^2^ relative to the SC. A 1.1-fold increase was observed at 0.6 µg/cm^2^, but no statistically significant effects were observed at 1.2 µg/cm^2^. Cytotoxicity was slightly lower in SC relative to IC (1.2-fold increase). For Triton X-100, no change in cytotoxicity relative to SC was observed at 6 and 9 µg/cm^2^, and even a slight decrease of 0.8-fold was observed at 3 µg/cm^2^. At the two highest concentrations (12 and 15 µg/cm^2^) cytotoxicity increased 2.7- and 3.1-fold, respectively.

### Secretion of inflammatory markers after exposure of BEAS-2B cells to surfactants

**Fig. 3.**
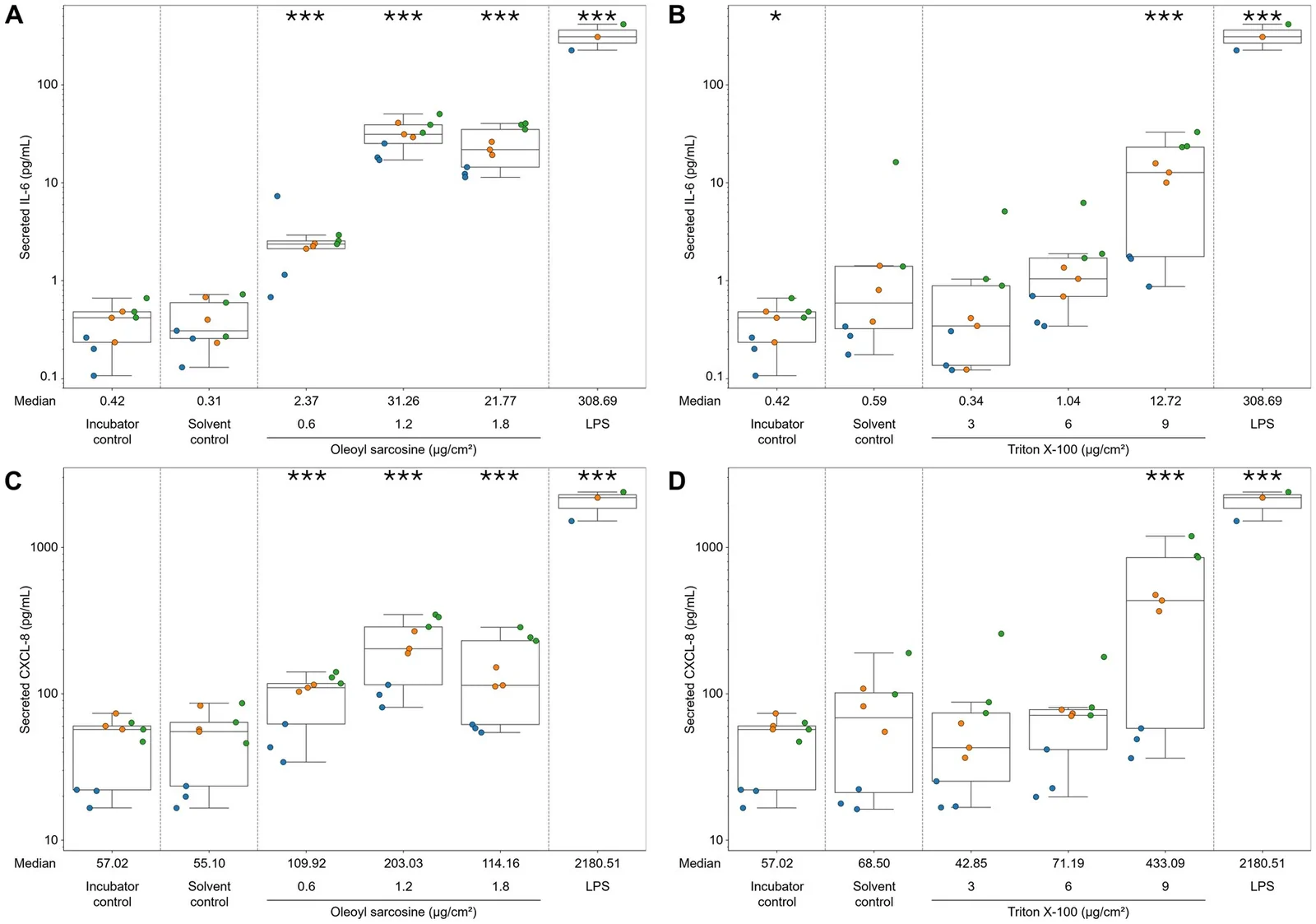
Effect of surfactant exposure on the secretion of inflammatory markers in BEAS-2B cells. Samples from the basolateral medium were collected to measure secreted Interleukin (IL)-6 and CXCL-8 following 24 h exposure to oleoyl sarcosine (A and C) or Triton X-100 (B and D). The two highest concentrations were not included because cell viability was below <10% (Fig. 1). The graphs show results from three experimental runs per condition (represented in different colors; N = 3), with each run having three technical replicates (n = 3). The asterisk (*) shows statistically significant differences compared with solvent control; *, *P *< 0.05; **, *P *< 0.01; ***, *P *< 0.001.

After exposure to oleoyl sarcosine, a significant increase in both IL-6 and CXCL-8 (IL-8) was observed at all three lower concentrations (0.6, 1.2, and 1.8 µg/cm^2^) (Fig. 3A and C). After exposure to Triton X-100, a significant increase in both IL-6 and CXCL-8 was observed at 9 µg/cm^2^, with no significant change observed at 3 and 6 µg/cm^2^ relative to SC (Fig. 3B and D). A slight decrease in IL-6 was observed in IC relative to SC. Overall, one experimental round seemed to yield slightly lower values of secreted cytokines (blue data points) but followed the same dose-response.

### Cell health after exposure of MucilAir tissues to surfactants

**Fig. 4.**
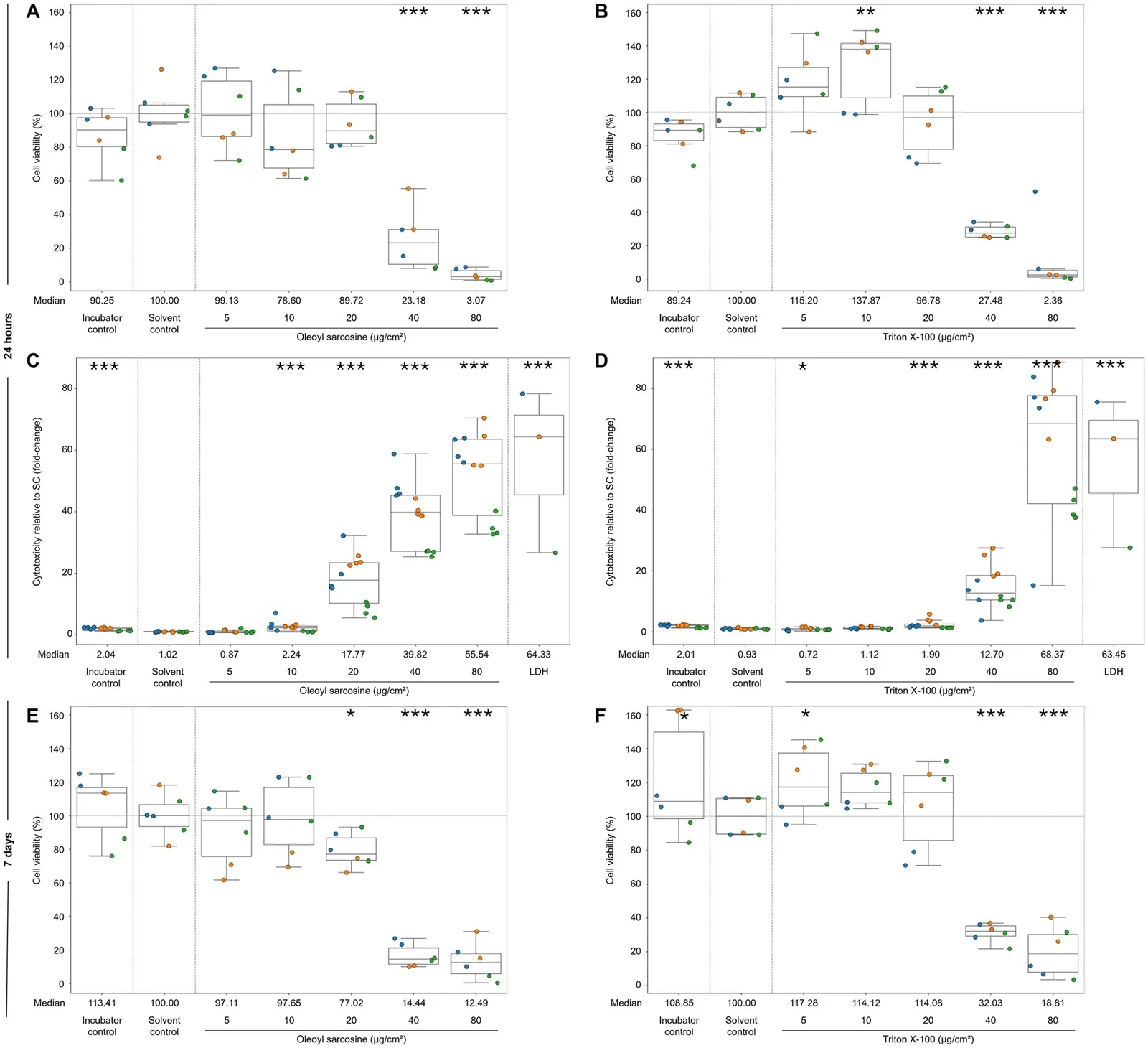
Effect on cell viability and cytotoxicity in bronchial MucilAir tissues after exposure to surfactants. MucilAir tissues were exposed to solvent control (SC) or 30 µl (91 µl/cm^2^) of five different concentrations (5, 10, 20, 40, 80 µg/cm^2^) of test substance for 24 h at the air–liquid interface. Following 24 h exposure to the test substances, cell viability was assessed using PrestoBlue assay and cytotoxicity was assessed using LDH assay. A and B show cell viability after 24 h for oleoyl sarcosine and Triton X-100, respectively. C and D show cytotoxicity response following 24 h exposure, and E and F show cell viability at 7 d postexposure. The graphs show results from three separate experimental runs for the two surfactants represented in different colors, with each run having either two (n = 2) or four replicates (n = 4). The results were normalized to solvent. The asterisk (*) shows statistical significance compared with SC; *, *P *< 0.05; **, *P *< 0.01; ***, *P *< 0.001.

For tissues treated with oleoyl sarcosine, cell viability after 24 h of exposure to the two highest concentrations, 40 and 80 µg/cm^2^, showed a statistically significant decrease to 23.2% and 3.1%, respectively. At the three lowest concentrations (5, 10, and 20 µg/cm^2^), no statistically significant change was observed (Fig. 4A). Similarly for tissues treated with Triton X-100, a marked decrease in cell viability was observed after 24 h of exposure for the two highest concentrations (27.5% at 40 µg/cm^2^ and 2.4% at 80 µg/cm^2^), whereas at the three lowest concentrations, no statistically significant decrease was observed (Fig. 4B). On the contrary, cell viability was statistically significantly increased at 10 µg/cm^2^ compared with SC. This increase likely reflects a transient increase in cellular metabolic activity, which can be elevated under stress conditions due to adaptive metabolic responses and induction of redox enzymes such as NQO1 via NRF2 signaling (Ross and Siegel 2021; Vieira-da-Silva and Castanho 2023).

For both surfactants, a significant increase in cytotoxicity was observed after 24 h of exposure to 20, 40, and 80 µg/cm^2^ (Fig. 4C and D). A difference in cytotoxicity was also observed in IC and at 10 µg/cm^2^ of oleoyl sarcosine and 5 and 20 µg/cm^2^ of Triton X-100 when compared with SC. These differences are potentially not biologically relevant but statistically significant due to the low variability in the SC group. Cytotoxicity was more pronounced for 20, 40, and 80 µg/cm^2^ for oleoyl sarcosine with 17.8-, 39.8-, and 55.5-fold increases compared with SC, respectively. For Triton X-100, cytotoxicity increased 12.7- and 68.4-fold at 40 and 80 µg/cm^2^, respectively.

At 7 d postexposure (Fig. 4E and F), cell viability remained significantly reduced at the two highest concentrations for both surfactants (14.4% and 12.5% for oleoyl sarcosine and 32.0% and 18.8% for Triton X-100, respectively). At the middle concentration of oleoyl sarcosine, cell viability was reduced to 77.0%. At all the other concentrations for oleoyl sarcosine and Triton X-100, cell viability remained around 100% compared with SCs for both. An increase in cell viability was observed for IC and at 5 µg/cm^2^ of Triton X-100. Cytotoxicity was not measured at 7 d.

### Secretion of inflammatory markers after exposure of MucilAir tissues to surfactants

**Fig. 5.**
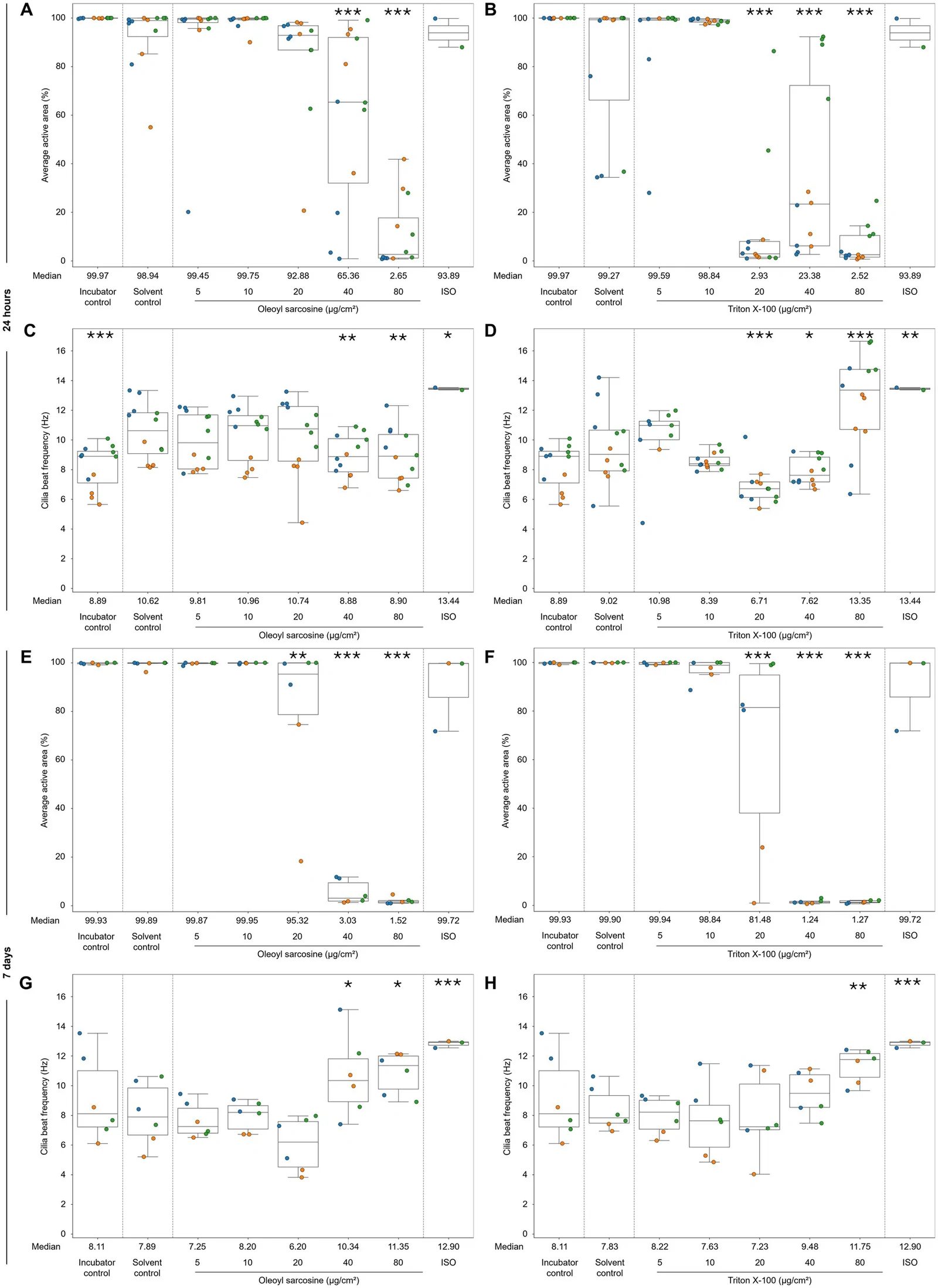
Assessment of inflammatory marker secretion in bronchial MucilAir tissues. After 24 h (A–D) of exposure or at 7 d (E–H) postexposure to oleoyl sarcosine or Triton X-100, secreted IL-6 and CXCL-8 in the basolateral medium were measured. The graphs show results from three separate experimental runs in different colors (N = 3), with each run having four individual replicates (n = 4) after 24 h of exposure and two replicates (n = 2) at 7d postexposure. The asterisk (*) shows statistical significance compared with solvent controls; *, *P *< 0.05; **, *P *< 0.01; ***, *P *< 0.001.

Exposure to both surfactants for 24 h (Fig. 5A–D) led to a statistically significant increase in IL-6 and CXCL-8 at all of the measured concentrations (5, 10, and 20 µg/cm^2^) compared with SC. No prolonged inflammatory processes were observed at 7 d postexposures with no statistically significant changes observed at any concentration except for the highest oleoyl sarcosine concentration, where a decrease was observed (Fig. 5E–H).

### Barrier integrity in bronchial MucilAir tissues

**Fig. 6.**
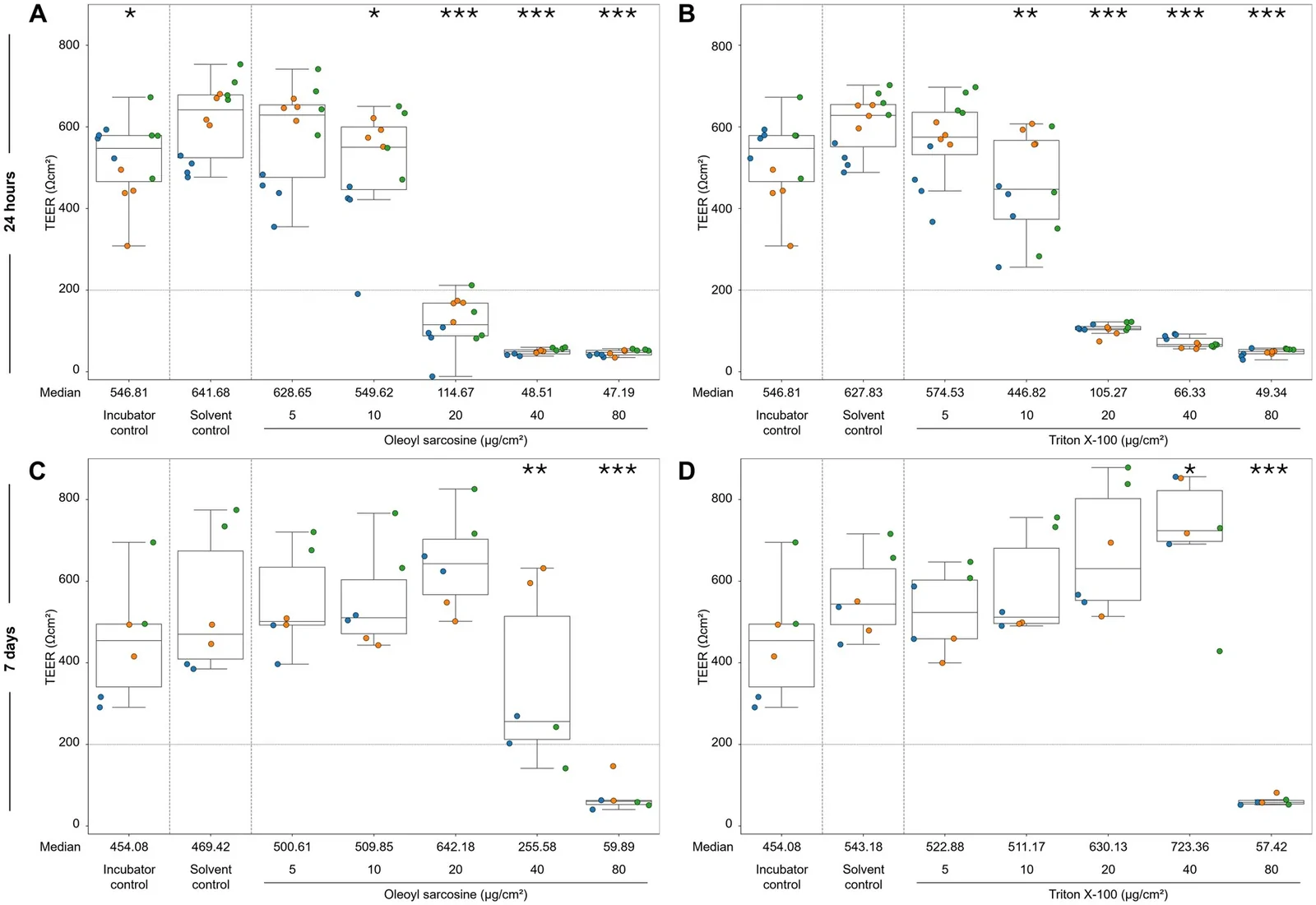
Assessment of barrier integrity in bronchial MucilAir tissues. TEER values of MucilAir tissues exposed to five different concentrations of oleoyl sarcosine (A) or Triton X-100 (B) for 24 h at the air–liquid interface. The graphs show measurements after 24 h of exposure (A and B) and 7 d after exposure (C and D) from three experimental runs (three different donors) represented in different colors, with each run having four technical replicates (n = 4) at the 24 h timepoint and two replicates (n = 2) at 7 d. TEER is represented as Ωcm^2^. The asterisk (*) shows statistical significance compared with solvent controls; *, *P *< 0.05; **, *P *< 0.01; ***, *P *< 0.001.

TEER was measured in MucilAir tissues 24 h before exposure (= pre-exposure) as a quality control similar to other studies (OECD 2021, 2025; Sharma et al. 2025), and after 24 h of exposure as well as 7 d postexposure to oleoyl sarcosine or Triton X-100 (Fig. 6). A value >200 Ωcm^2^ is generally accepted to indicate a tight barrier for MucilAir, per the manufacturer’s recommendation. The TEER values for SC remained similar with expected minor fluctuation throughout the experimental period. The electrical resistance of the epithelial barrier also remained above the threshold of 200 Ωcm^2^ for the tissues exposed to the two lowest concentrations (5 and 10 µg/cm^2^) of both surfactants although there was a statistically significant decrease at 10 µg/cm^2^ compared with SC. The TEER values dropped below the threshold of 200 Ωcm^2^ for all tissues after 24 h of exposure to 20, 40, and 80 µg/cm^2^ of both surfactants. For the highest tested concentration (80 µg/cm^2^), the TEER does not show recovery after 7 d but the tissues treated with 20 µg/cm^2^ of either surfactant showed recovery to TEER values similar to pre-exposure or SC measurements.

### Cilia beating frequency and active area in bronchial MucilAir tissues

**Fig. 7.**
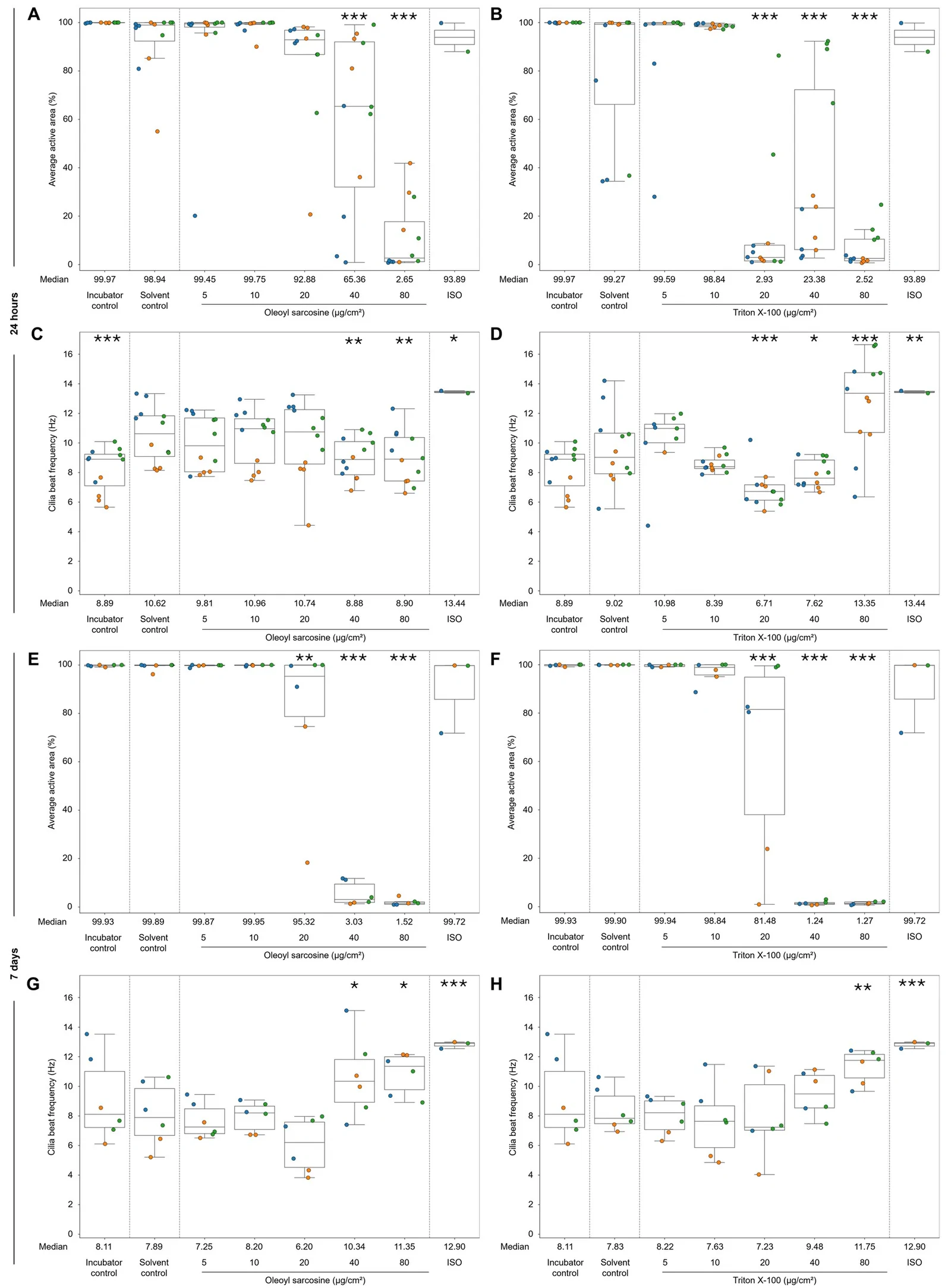
Assessment of average active area (AAA) and cilia beating frequency (CBF) in bronchial MucilAir tissues. The graphs show AAA and CBF measurements after 24 h of exposure to oleoyl sarcosine (A, C) or Triton X-100 (B, D) and 7 d after exposure (E, G for oleoyl sarcosine and F, H for Triton X-100) from three experimental runs (three different donors) represented in different colors, with each run having at least two replicates. AAA is presented as percentage (%) and CBF in hertz (Hz). Note: Due to a data handling error, three data points at 5 µg/cm^2^ and the ISO control are missing at 24 h for Donor 2 (orange). The asterisk (*) shows statistical significance compared with solvent control; *, *P *< 0.05; **, *P *< 0.01; ***, *P *< 0.001.

Average active area (AAA) indicates the average area of tissues containing viable cilia, and cilia beating frequency (CBF) measures the frequency of cilia movement within the AAA. When combined, these metrics may reveal the effects of exposure to the test chemicals on the mucociliary clearance of the tissues (Fig. 7). Since substantial cell death was observed for both surfactants at the two highest concentrations in several assays (cell viability, cytotoxicity, TEER, and in histology), any AAA and CBF measurements reported for these concentrations should be interpreted with caution. Furthermore, due to a data handling error, the ISO control at 24 h for donor 2 and three of four data points for tissues exposed to 5 µg/cm^2^ of Triton X-100 (donor 2 only) were unavailable.

For tissues treated with up to 20 µg/cm^2^ of oleoyl sarcosine, no statistically significant differences were observed after 24 h of exposure. At 40 µg/cm^2^, the AAA measurement was variable but showed an overall decrease (65.4%) and reached 2.7% at 80 µg/cm^2^. This decrease was even more marked at 7 d postexposure (3.0% and 1.5%, respectively). A statistically significant decrease in CBF was observed for the same concentrations after 24 h of exposure and an increase was observed at 7 d postexposure.

For Triton X-100-exposed MucilAir, a similar trend was observed for AAA, except that a marked decrease to 2.9% was already observed at the middle concentration of 20 µg/cm^2^ after 24 h of exposure. At 40 µg/cm^2^, variability was similar to oleoyl sarcosine-exposed tissue but with a lower overall decrease (23.4%). By 7d postexposure, the AAA was higher again (with 81.5%) but still decreased compared with the SC. Although CBF was decreased at 20 and 40 µg/cm^2^, an increase was observed at the highest concentration after 24 h and 7 d of exposure.

### Morphological changes in bronchial MucilAir tissues

**Fig. 8.**
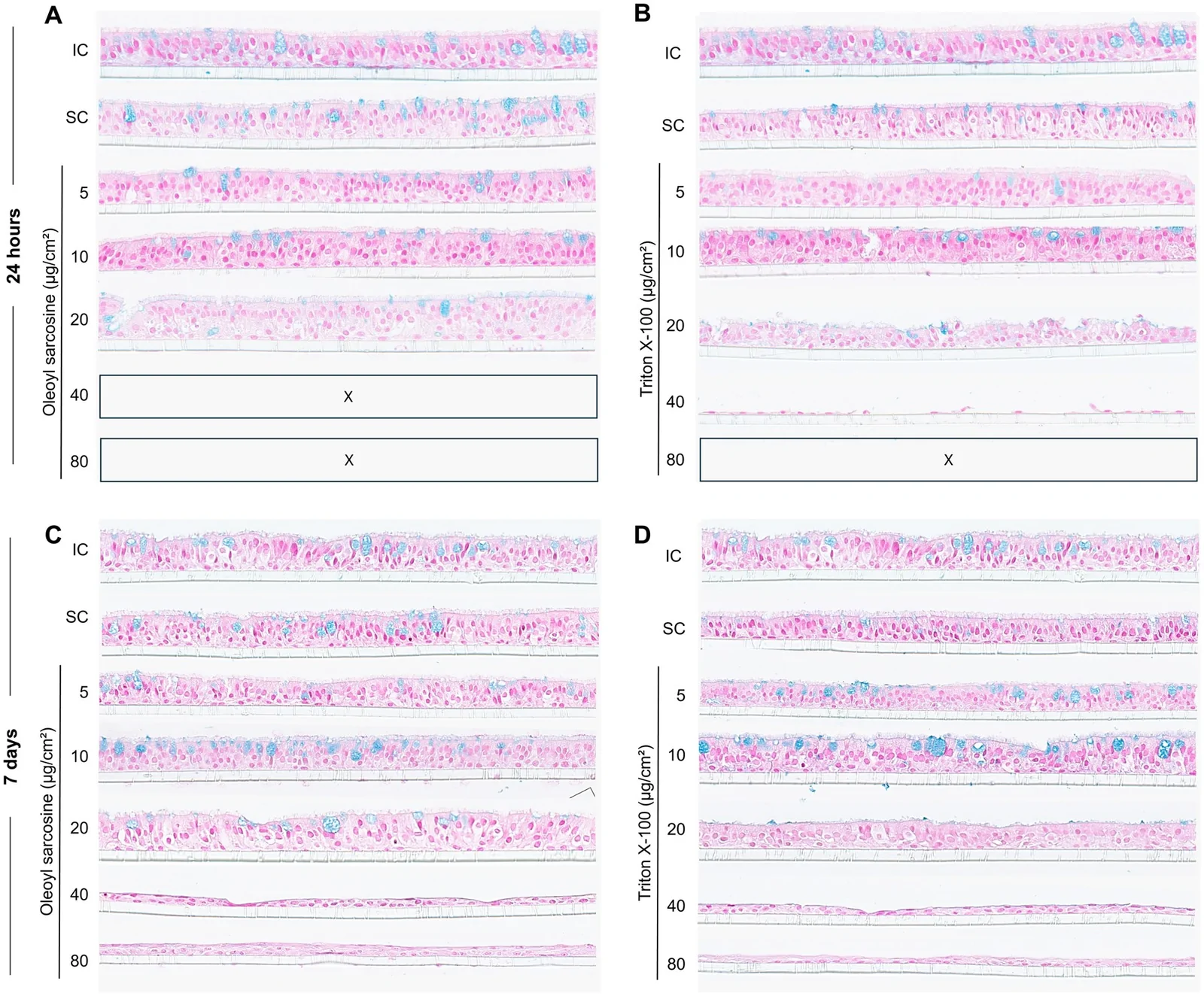
Cross-sections of bronchial MucilAir tissues. MucilAir tissues were exposed to controls (IC and SC) or 30 µl (91 µl/cm^2^) of five different concentrations of oleoyl sarcosine or Triton X-100 for 24 h at the air–liquid interface. After 24 h or 7 d postexposure, two inserts were fixed and processed using Alcian Blue/Periodic Acid Schiff/Hematoxylin stain for imaging. Figure 6A and B show the tissues exposed to oleoyl sarcosine after 24 h and 7 d, respectively. Figure 6C and D show the tissues exposed to Triton X-100 after 24 h and 7 d, respectively. These images are from one experimental run. IC, incubator control; SC, solvent control.

Histology at 24 h and 7 d postexposure to all treatments was conducted. The IC in Fig. 8 shows the typical pseudostratified structure of airway epithelium comprising basal cells, ciliated cells, and mucus-secreting goblet cells. No noticeable changes were observed in the SC and IC at 24 h or 7 d postexposure. Similarly, for tissues treated with the two lowest concentrations (5 and 10 µg/cm^2^) of oleoyl sarcosine, no obvious changes were observed at 24 h or 7 d postexposure (Fig. 8A and C). Tissues treated with 20 µg/cm^2^ of oleoyl sarcosine did not show substantial changes after 24 h of exposure but loss of cilia was observed after 7 d of exposure. No histological images are available for the tissues exposed to 40 and 80 µg/cm^2^ due to the strong cytotoxicity observed. Nevertheless, at 7 d the remaining cells on the membrane seem to proliferate and cover large parts of the membranes as monolayers.

Morphological changes after exposure to Triton X-100 followed similar trends as observed in tissues treated with oleoyl sarcosine where no changes were observed at the lower concentrations and extensive damage to tissues was observed at the highest concentrations (Fig. 8B and D). Tissues exposed to 20 µg/cm^2^ of Triton X-100 showed damage to the cell layer with loss of stratification after 24 h of exposure and loss of cilia was observed 7 d postexposure.

### Derivation of benchmark concentration

The in vitro data for each cellular effect was used to derive benchmark dose (BMD_1SD_) using BMDS online (version 25.1) (US EPA 2025a). BMDL_1SD_ values of cytokine secretion (IL-6 and CXCL-8) were generally lower than other cellular effects for all 24 h assessments across both cell models. In BEAS-2B cells, exposure to oleoyl sarcosine for 24 h led to lower BMDL_1SD_ values for all cellular effects compared with Triton X-100 exposures (Table 4). In MucilAir, Triton X-100 exposure for 24 h led to lower BMDL_1SD_ values in five out of seven cellular effects compared with oleoyl sarcosine exposure. Only cytotoxicity and CXCL-8 secretion were lower for oleoyl sarcosine exposures. At 7 d postexposure, oleoyl sarcosine had lower BMDL_1SD_ values than at 24 h for all endpoints except IL-6, CXCL-8, and TEER; in Triton X-100-exposed tissues, only AAA yielded a lower BMDL_1SD_ at 7 d.

**Table 4.** Benchmark dose (BMD_1SD_; in parentheses) and BMD lower limit (BMDL_1SD_) values shown in µg/cm^2^.

	Oleoyl sarcosine			Triton X-100		
	BEAS-2B	MucilAir		BEAS-2B	MucilAir	
Cellular effect	24 h	24 h	7 d	24 h	24 h	7 d
*Cell viability*	0.71 (0.83)	17.18 (26.99)	14.77 (18.94)	6.24 (6.53)	4.20^*^ (9.1^*^)	16.46 (21.52)
*Cytotoxicity*	0.92 (1.13)	5.29 (6.96)	–	5.49 (7.12)	7.81 (11.73)	–
*IL-6*	0.24 (0.42)	5.52 (9.32)	10.59 (19.42)	3.88 (5.77)	1.15 (1.79)	10.72^^^ (41.28)
*CXCL-8*	0.53 (0.99)	3.59 (5.33)	3.60 (6.60)	5.16 (6.90)	2.94 (3.57)	8.08^^^ (18.89)
*TEER*	–	9.18 (11.12)	23.75 (39.68)	–	4.31 (5.87)	40.02 (67.06)
*AAA*	–	16.04 (25.94)	12.91 (16.62)	–	9.33 (15.26)	8.91 (13.93)
*CBF*	–	31.73 (74.05)	7.14 (19.66)	–	4.98 (9.11)	11.54^^^ (56.03)

IL, interleukin; TEER, transepithelial electrical resistance; AAA, average active cilia area; CBF, cilia beat frequency; NOAEC, no observable adverse effect concentration; LOAEC, lowest observable adverse effect concentration. TEER, CBF, AAA were not measured in BEAS-2B, and LDH was not measured at 7 d postexposure in MucilAir.

* MucilAir viability data was unsuitable for BMDS modeling and so the NOAEC and LOAEC (in parentheses) values are reported.

^ High variability observed in the dataset which decreases confidence.

### Derivation of human equivalent concentrations

HECs for acute, point-of-contact irritation were calculated using the above BMDL_1SD_ values derived from endpoints measured at 24 h (Table 5). MPPD simulations were tailored to accommodate the physicochemical properties of oleoyl sarcosine (nonhygroscopic) and Triton X-100 (hygroscopic) across a range of reported aerosol sizes under potential occupational and consumer inhalation exposure scenarios. Oleoyl sarcosine exposures are expected when working with metalworking fluid aerosols or rust inhibitor sprays. A literature review identified MMAD values ranging from 3.3 to 8 µm, so simulations with three particle sizes (3, 6, 8 µm) were conducted with a representative GSD of 2.5. When occupational exposures were simulated using the EPA default ventilation rate (10 m^3^/8 h), the total deposition fraction ranged from 0.80 for 3 µm particles to 0.85 for 6 µm particles (Table S5). In each case, the extrathoracic (ET) region was the primary target of deposition (deposition fraction: 0.66 to 0.76). Slightly lower deposition fractions were observed when simulating consumer exposures using the general EPA default ventilation rate (20 m^3^/24 h; Table S6).

**Table 5.** Human equivalent concentration values for the extrathoracic region (HEC_ET_; mg chemical/m^3^ air) were computed for a range of particle size distributions for both consumer (top) and occupational (bottom) exposure conditions.

HEC_ET_ values for oleoyl sarcosine and Triton X-100 (mg chemical/m^3^) for consumer exposure conditions (Ventilation rate = 20 m³/24 h)							
		Oleoyl sarcosine			Triton X-100		
	Cellular effect	MMAD: 3 µm (GSD: 2.5)	MMAD: 6 µm (GSD: 2.5)	MMAD: 8 µm (GSD: 2.5)	MMAD: 2 µm (GSD: 2)	MMAD: 3 µm (GSD: 2)	MMAD: 4 µm (GSD: 2)
**BEAS-2B**	Cell viability	0.03	0.02	0.02	0.26	0.21	0.19
	Cytotoxicity	0.03	0.03	0.03	0.22	0.18	0.16
	IL-6	0.01	0.01	0.01	0.16	0.13	0.12
	CXCL-8	0.02	0.02	0.02	0.21	0.17	0.15
**MucilAir**	Cell viability	0.63	0.52	0.51	0.17	0.14	0.13
	Cytotoxicity	0.19	0.16	0.16	0.32	0.26	0.23
	IL-6	0.20	0.17	0.16	0.05	0.04	0.03
	CXCL-8	0.13	0.11	0.11	0.12	0.10	0.09
	TEER	0.34	0.28	0.27	0.18	0.14	0.13

HEC_ET_ values for oleoyl sarcosine and Triton X-100 (mg chemical/m^3^) for occupational exposure conditions (Ventilation rate = 10 m³/8 h)							
		Oleoyl sarcosine			Triton X-100		
	Cellular effect	MMAD: 3 µm (GSD: 2.5)	MMAD: 6 µm (GSD: 2.5)	MMAD: 8 µm (GSD: 2.5)	MMAD: 2 µm (GSD: 2)	MMAD: 3 µm (GSD: 2)	MMAD: 4 µm (GSD: 2)
**BEAS-2B**	Cell viability	0.05	0.04	0.04	0.46	0.38	0.35
	Cytotoxicity	0.06	0.05	0.05	0.40	0.34	0.31
	IL-6	0.02	0.01	0.01	0.28	0.24	0.22
	CXCL-8	0.03	0.03	0.03	0.38	0.32	0.29
**MucilAir**	Cell viability	1.13	0.98	0.98	0.31	0.26	0.24
	Cytotoxicity	0.35	0.30	0.30	0.57	0.48	0.44
	IL-6	0.36	0.32	0.31	0.08	0.07	0.06
	CXCL-8	0.24	0.21	0.20	0.22	0.18	0.16
	TEER	0.60	0.53	0.52	0.32	0.26	0.24

Calculations are based on the 24 h BMDL_1SD_ values (or NOAEL values when data were not amenable to BMD modeling, see Table 4) and the EPA MPPD model predictions (Tables S5–S8). Note that these HEC_ET_ values are independent of product formulations.

MMAD, mean mass aerodynamic diameter; GSD, geometric SD; IL, interleukin; TEER, transepithelial electrical resistance.

Triton X-100 exposures are expected during use of industrial spray cleaners. A recent evaluation of airborne aerosols produced from trigger cleaning sprays revealed MMAD values ranging from 2.1 to 3.7 µm; accordingly, the simulations included 2, 3, and 4 µm liquid hygroscopic aerosols using a representative GSD of 2. During simulations of occupational and consumer exposure scenarios, the total deposition fraction ranged from 0.97 to 0.98 across all particle sizes (Tables S7 and S8). As in the case of oleoyl sarcosine, the ET region was the primary target of deposition (0.59 to 0.78), and so HEC_ET_ values will be reported for both chemicals.

## Discussion

The uptake of new methods for regulatory decision-making relies on building confidence in their use (van der Zalm et al. 2022; ICCVAM 2024). The results of the present study build scientific confidence through technical characterization and demonstrating fitness-for-purpose in the use of two human cell-based systems to assess the respiratory tissue irritation of two surfactants. Oleoyl sarcosine and Triton X-100 served as reference chemicals given that previous repeat-dose inhalation studies indicated point-of-contact respiratory tract toxicity and inflammation (US EPA 2024). Both human airway cell models confirmed these findings following liquid application exposure and provided human-relevant hazard points of departure (PODs) that reduce uncertainty in evaluating potential inhalation risks.

Although aerosol exposures may be considered more physiologically relevant, they require specialized equipment and expertise in aerosol generation and characterization (Petersen et al. 2021). Liquid exposure by pipetting simplifies dosimetry and is more accessible for laboratories worldwide (Jeong et al. 2019, 2021; McGee Hargrove et al. 2021; Ruijter et al. 2025). Aerosol-exposed BEAS-2B cells showed dose-dependent effects, but viability did not fall below 50% (Fig. S1 and Table S2). Furthermore, aerosolizing viscous substances proved to be challenging as it introduced high variability in the measured deposition of each test substance (Table S4) and sufficient concentrations of each test substance could not be achieved (especially for Triton X-100). Although this variability could be reduced with optimization, no obvious differences were observed in these studies or similar studies in BEAS-2B cells (Lindenberg et al. 2019). Given the comparable outcomes, liquid exposures were selected for the remainder of the study. A volume of 30 µl (91 µl/cm^2^) was selected to ensure full coverage of the entire cell surface while limiting potential side effects observed with higher volumes (Mallek et al. 2023). The liquid was fully absorbed by the tissues within 24 h, reestablishing the ALI, and 30 µl reduces pipetting inaccuracies compared with lower volumes (Guan et al. 2023). This same volume of liquid was used in the first published case study using an RHRE for regulatory decision-making (McGee Hargrove et al. 2021; US EPA 2021; OECD 2022).

The impact of the surfactants on the in vitro test systems was assessed using a suite of complementary cellular endpoints to assess multiple modes of injury and improve interpretability. Cell health was assessed with cell viability and cytotoxicity assays which increase confidence in the results without adding substantial time or cost, and can help detect potential chemical interference with the assays. Barrier integrity was monitored measuring TEER, with high values generally reflecting healthy cells, whereas substantial TEER reductions can permit leakage of the cell culture medium from the basal compartment into the apical compartment. Further work is needed to understand how minor or moderate changes in TEER may predict adverse health outcomes, but TEER values below 200 to 250 Ωcm^2^ are commonly considered indicative of a compromised barrier (Metz et al. 2020; Sharma et al. 2025). The proinflammatory mediators interleukin (IL)-6 and CXCL-8 were also measured, which are produced at low baseline levels in epithelial cells in vitro but are readily induced by nonspecific cellular stress, injury, mechanical, or metabolic perturbation (Quinn et al. 2018; Al Yazeedi et al. 2025; Meier and Brieger 2025). Thus, transient increases in epithelial secretion may reflect a general stress response. Ciliary activity, including ciliary beat frequency and ciliary average active area, was also measured to assess potential impairment to mucociliary clearance capacity. For example, a decrease in ciliary average active area may indicate loss of cilia that would also compromise mucociliary clearance, potentially leading to coughing, indicating respiratory irritation. Lastly, histology provides confirmatory and quality control information, but is limited by reliance on expert scoring from a pathologist to be used in a (semi-) quantitative manner. Altogether, this study presents a viable, human-relevant approach for assessing respiratory irritation by inhaled chemicals.

A concentration-dependent response was observed in all cellular effects after exposure to surfactants in both cell systems. In BEAS-2B cells, a clear difference in the toxicities of the two surfactants was observed. Oleoyl sarcosine had BMDL_1SD_ values between 0.2 and 0.9 µg/cm^2^ and Triton X-100 between 3.9 and 6.2 µg/cm^2^. Nonionic and anionic surfactants seem to show toxic effects at concentrations below their critical micelle concentration (CMC); 74 µM (approximately 2.4 µg/cm^2^) for oleoyl sarcosine and 260 µM (15 µg/cm^2^) for Triton X-100 in H_2_0 at 25°C. In addition, ionic solutions—such as basal cell culture medium (which contains salts, sugars, and vitamins) or saline—can change the behavior of surfactants, including lowering the CMC, especially of ionic surfactants (Akhlaghi et al. 2019; Belhaj et al. 2020). Therefore, the higher concentrations of Triton X-100 needed to cause toxic effects in BEAS-2B cells, compared with oleoyl sarcosine, may be explained based on the CMC or its interaction with the liquid vehicle (cell culture medium).

Unlike in BEAS-2B cells, Triton X-100 generally produced lower BMDL_1SD_ values in MucilAir which reflects the expected difference in potency based on previous OECD test guideline (TG) 431, 439, and 492B results (Table S9). One explanation for the difference between BEAS-2B and MucilAir may be the interactions of the differently charged surfactants with the mucus layer on the RHRE model. Even though 30 µl (91 µl) of liquid is applied to the apical side of the tissues, diluting most of the mucus, some mucus will remain on top of the cells and more will likely be released from the goblet cells. Mucus can act as a selective barrier to particles and molecules, and physico-chemical properties, such as size, pH, ionic strength, charge, and hydrophilicity, govern the translocation of these substances across the mucus barrier (Sigurdsson et al. 2013; Leal et al. 2017; Dong et al. 2020). In addition, MucilAir are covered in beating cilia. Those cilia carry a glycocalyx consisting of sialic acid and sulfate groups, providing a strongly negatively charged milieu around the cells (Ridley and Thornton 2018; Chatterjee et al. 2020). Therefore, the hydrophobicity of oleoyl sarcosine paired with the negative milieu around MucilAir could potentially explain the differences observed in this study. In addition, differences in used solvents and cell systems lead to variations in the microenvironment (e.g. salts, secreted lipids and proteins, cell debris, pH, etc.), which can alter surfactant surface activity and thereby potentially affect the interaction with intact tissues (Gelamo et al. 2002; Pugnaloni et al. 2004; Goodarzi and Zendehboudi 2019).

For MucilAir, cellular effects from a single exposure were assessed at 24 h and again at 7 d. Generally, partial recovery of cellular health effects was observed at 7 d for lower doses, except when the cells died, as expected. For this study, the 7 d timepoint produced highly variable data and did not provide additional useful information compared with 24 h exposure. As a result, HEC values were calculated using the 24 h endpoints. The extrathoracic region was the primary deposition target and so HEC_ET_ values were reported even though bronchial-derived models were used. This is a reasonable approach given the similarities between nasal and bronchial mucociliary epithelium tissues (McDougall et al. 2008; Iskandar et al. 2013; Moreau et al. 2022; Pohl et al. 2025; Van Woudenbergh et al. 2025).

In conclusion, the IVIVE workflow presented here demonstrates a generalized method using the EPA MPPD v2.0 model to translate in vitro PODs for respiratory irritants when inhalation exposures to aerosols are expected. A literature review informed the potential use cases and expected airborne particle size distributions for both Triton X-100 and oleoyl sarcosine. HEC values are dependent upon the particle size and activity characteristics, and so additional simulations could be performed to target additional populations and exposure scenarios for specific product types. Although the EPA MPPD model parameters reported here are specific to Triton X-100 and oleoyl sarcosine, the IVIVE workflow is generic and can be applied to similar respiratory irritants by adjusting the particle parameters and ventilation rates to accommodate specific use cases and inhalation exposure scenarios. When these factors are appropriately captured during inhalation dosimetry modeling, resulting HEC values produced from in vitro data may be valuable for hazard assessment.

The appropriate in vitro study design will depend on the test chemical and purpose of testing, and the results show that, for the chemicals tested, a cell line and RHRE are both valuable model systems to screen point-of-contact respiratory irritants. Although cell systems that do not produce mucus may give a good indication about a substance’s toxicity in the proximal respiratory tract, our study showed that the interaction with the RHRE’s mucus and cilia can lead to different results, especially when charged substances are tested.

The approach presented in this paper could be particularly valuable in understanding acute point-of-contact effects resulting from a single exposure and can be applied to fill data gaps for structurally diverse, data-poor surfactants. Overall, the results of this study demonstrate the ability of in vitro systems to reliably generate data on the point-of-contact respiratory effects of surfactants with known irritation potential, establish confidence in the use of liquid exposure to these systems, and show how to translate the in vitro results to human-equivalent concentrations to evaluate inhalation hazards. When PODs from in vitro respiratory irritation assays are translated using mechanistic inhalation dosimetry models, they can provide valuable insights for understanding the potential respiratory effects of inhaled chemicals in humans.

## Supplementary Material

kfag118_Supplementary_Data
