# Supplementary material for: Human in vitro airway models to assess point-of-contact respiratory toxicity of surfactants and derive human equivalent concentrations after single dose exposure
Source: Toxicol Sci. 2026 Sep 10;209(9):kfag118. doi: 10.1093/toxsci/kfag118 (PMC13616041; doi:10.1093/toxsci/kfag118)
Supplement: Supplementary file 1 [file kfag118_supplementary_data.docx]

**Supplementary document**

**Human *in vitro* airway models to assess point-of-contact respiratory toxicity of surfactants and derive human equivalent concentrations after single dose exposure**

Andreas O. Stucki^1^<https://orcid.org/0000-0002-0997-8502>, Monita Sharma^1^<https://orcid.org/0000-0003-2859-2581>, Jessica R. Murray^2^<https://orcid.org/0000-0002-3936-4982>, Sandra Verstraelen^3^<https://orcid.org/0000-0003-0529-8208> , Evelien Frijns^3^, Karen Hollanders^3^, An Jacobs^3^, Sylvie Remy^3^<https://orcid.org/0000-0003-0322-9620>, Nuria Roldan^1^<https://orcid.org/0000-0002-3728-804X>, Jo Van Laer^3^, Miyoung Yoon^4^<https://orcid.org/0000-0002-3653-3488>, Keith Salazar^4^<https://orcid.org/0000-0002-0086-9916?lang=en>, Natalia Garcia-Reyero Vinas^4^<https://orcid.org/0000-0002-1667-5294>, Anna Lowit^4^, Amy J. Clippinger^1^<https://orcid.org/0000-0003-3623-1196>

^1^PETA Science Consortium International e.V., 70499 Stuttgart, Germany.

^2^U.S. Environmental Protection Agency, Office of Chemical Safety and Pollution Prevention, Risk Assessment Support Division, Research Triangle Park, NC 27711, United States.

^3^Environmental Intelligence Unit, Flemish Institute for Technological Research (VITO), BE-2400 Mol, Belgium.

^4^U.S. Environmental Protection Agency, Office of Chemical Safety and Pollution Prevention, Washington, D.C. 20460, United States.

Corresponding author: Andreas O. Stucki; [andreass@thepsci.eu](mailto:andreass@thepsci.eu)

# Supplementary materials and methods

**Supplementary Table 1.** Materials and equipment used in this study, their source/manufacturer, and catalog number.

| **Product name** | **Source/Manufacturer** | **Catalog #** |
| --- | --- | --- |
| Acetic acid | Supelco, Pennsylvania, USA | 1.0063.1000 |
| Acetonitrile | Biosolve, Valkenswaard, The Netherlands | 001204102BS |
| Acquity UPLC BEH C18 column | Waters, Milford, Massachusetts, USA | 186002352 |
| Albumin, human serum | Sigma-Aldrich, Saint Louis, Missouri, USA | A9731 (Lot: SLCM5919) |
| Ammoniumacetate | VWR, Radnor, Pennsylvania, USA | 84885 |
| Basler acA1300-200µm USB3 video camera | Ammons Engineering, Clio, Michigan, USA | n/a |
| BEAS-2B cell line | American Type Culture Collection (ATCC), Manassas, Virginia, USA | CRL-9609 |
| BEBM™ Bronchial Epithelial Cell Growth Basal Medium | Lonza, Basel, Switzerland | CC-3171 |
| BEGM™ Bronchial Epithelial Cell Growth Medium BulletKit™ | Lonza, Basel, Switzerland | CC-3170 |
| Calcium chloride dihydrate | Merck Production Chemicals, Darmstadt, Germany | 1.42002.1000 |
| Caps for glass vials | Thermo Fisher Scientific, Waltham, Massachusetts, USA | 9150868 |
| Cell culture flask, 250 ml, 75 cm², PS | Greiner Bio-One nv, Kremsmünster, Austria | 658170 |
| Cell culture multi-well plate, 24-well, polystyrene, clear | Greiner Bio-One nv, Kremsmünster, Austria | 662160 |
| cellQuart^®^ 24 well insert 0.4 µm PET clear | SABEU GmbH & Co. Kg, Northeim, Germany | 932 04 12 |
| Cilia-X software | Epithelix Sàrl, Geneva, Switzerland | CiliaX V1.0 |
| Collison atomizer | Topas, Dresden, Germany | ATM-220 |
| Cytotoxicity LDH Assay Kit-WST | Tebu-bio, Le Perray-en-Yvelines, France | Ck12-05 |
| Dimethyl sulfoxide (DMSO) | Sigma-Aldrich, Saint Louis, Missouri, USA | D5879 |
| Ethanol (99,8% denatured, water free) | Acros Organics, Geel, Belgium | 448440025 |
| EVOM Manual voltohmmeter | World Precision Instruments Germany, GmbH, Friedberg, Germany | EVM-MT-03-01 |
| EVOM STX-4 electrodes | World Precision Instruments Germany, GmbH, Friedberg, Germany | STX4 |
| Fibronectin, from human plasma | Sigma-Aldrich, Saint Louis, Missouri, USA | F2006 |
| Fisherbrand™ EPA Screw Neck Vial, Glass | Thermo Fisher Scientific, Waltham, Massachusetts, USA | 10000782 |
| Formaldehyde 4% | Avantor, Radnor, Pennsylvania, USA | 9713 |
| Gibco™ DPBS, calcium, magnesium | Thermo Fisher Scientific, Waltham, Massachusetts, USA | 11580456 |
| Glass vials | Thermo Fisher Scientific, Waltham, Massachusetts, USA | 11090500 |
| Gibco™ HEPES (1M) | Thermo Fisher Scientific, Waltham, Massachusetts, USA | 15630080 |
| Isoproterenol hydrochloride | Merck KGaA, Darmstadt, Germany | PHR2722 (Lot: LRAD5082) |
| LCGC Certified Clear Glass 12 x 32 mm Screw Neck Vial | Millipore, Burlington, Massachusetts, USA | 186000307C |
| Lipopolysaccharides (LPS) from Escherichia coli O111:B4 | Sigma-Aldrich, Saint Louis, Missouri, USA | L4391 (Lot. 0000252542) |
| Mass flow controllers | Bronkhorst, Veenendaal, The Netherlands | Low-∆P-Flow / EL-flow |
| Methanol | Sigma-Aldrich, Saint Louis, Missouri, USA | 1.06018 |
| Methanol | Biosolve, valkenswaard, The Netherlands | 0013684102BS |
| Microplate, 96 well, PS, F-bottom (chimney well) | Greiner Bio-One nv, Kremsmünster, Austria | 655094 |
| MilliQ water | Merck KGaA, Darmstadt, Germany | IQ7005 |
| MucilAir™ | Epithelix Sàrl, Geneva, Switzerland | EP01MD |
| MucilAir™ Medium | Epithelix Sàrl, Geneva, Switzerland | EP05MM |
| Multi-mode microplate reader CLARIOstar Plus | BMG Labtech, Ortenberg, Germany | n/a CLARIOstar V5.21 |
| Oleoyl sarcosine | Tokyo Chemical Industry (TCI) Europe N.V., Zwijndrecht, Belgium | O0113 (Lot: EEY8F-RK) |
| Dulbecco’s phosphate buffered saline | Sigma-Aldrich, Saint Louis, Missouri, USA | n/a |
| Potassium Chloride (KCl) | Sigma-Aldrich, Saint Louis, Missouri, USA | P3911 |
| PrestoBlue™ Cell Viability Reagent | Thermo Fisher Scientific, Waltham, Massachusetts, USA | A13261 |
| Screw cap tubes (25 mL) | Eppendorf, Hamburg, Germany | 30122437 |
| Sealing Tape | Nunc, Sjaelland, Denmark | 236366 |
| Sisson-Ammons Video Analysis (SAVA) software | Ammons Engineering, Clio, Michigan, USA | n/a |
| Sodium chloride | Sigma-Aldrich, Saint Louis, Missouri, USA | S7653 |
| Test resistor | World Precision Instruments Germany, GmbH, Friedberg, Germany | n/a |
| Total hydrocarbon analyzer | JUM Engineering GmbH, Karlsfeld, Germany | 3-300 |
| Triton™ X-100 | Sigma-Aldrich, Saint Louis, Missouri, USA | X100 (Lot: WXBD9346V) |
| TrypLE^™^ Express Enzyme, 1X | Thermo Fisher Scientific, Waltham, Massachusetts, USA; Gibco™ | 12604013 |
| Ultra Performance Liquid Chromatography (UPLC)-tandem MS using a Waters H-class Acquity UPLC system | Waters, Milford, Massachusetts, USA | n/a |
| Ultrapure water | Millipore, Burlington, Massachusetts, USA | n/a |
| VITROCELL^®^ 6/4 CF Stainless Steel Exposure Module | VITROCELL^®^ Systems, Waldkirch, Germany | n/a |
| VitroCol^®^ | Sigma-Aldrich, Saint Louis, Missouri, USA | 5008 |
| V-PLEX Custom Human Biomarkers Proinflammatory Panel (human): Human IL-2, Human IL-6, Human IL-8, and Human TNF-α | Meso Scale Diagnostics, Rockville, Maryland, USA | K151A9H-2 |
| Waters Xevo tandem mass spectrometer | Waters, Milford, Massachusetts, USA | n/a |
| Zeiss Axiovert 10 microscope | Zeiss, Oberkochen, Germany | n/a |

**Supplementary Table 2.** Numbers of technical replicates used per cellular effect and time point (before/after exposure) for BEAS-2B cells and MucilAir for liquid exposures. * For Donor 2, only 1 replicate was available for CBF/AAA of Triton X-100 due to data handling errors. Abbreviations: NA, Not Assessed; TEER, Transepithelial Electrical Resistance; CBF, Cilia Beating Frequency; AAA, Average Active Area.

| **Cellular effect** | **Biological test system** | | | |
| --- | --- | --- | --- | --- |
|  | **BEAS-2B** | **MucilAir** | | |
|  | *24 h* | –*24 h* | *24 h* | *7 d* |
| Cell viability | 3 | NA | 2 | 2 |
| Cytotoxicity | 3 | NA | 4 | NA |
| Pro-inflammatory markers | 3 | NA | 4 | 2 |
| TEER | NA | 4 | 4 | 2 |
| CBF, AAA | NA | 4 | 4* | 2 |
| Histology | NA | NA | 2 | 2 |

**Supplementary Table 3.** Donor information provided for MucilAir tissues by the manufacturer.

| **Date of exposure** | **Color in graphs** | **Sex** | **Age** | **Pathology reported** | **Smoker** | **Morphology** |
| --- | --- | --- | --- | --- | --- | --- |
| 22 May 2024 | ⚫ (Blue) | Male | 42 | None | No | Normal with some mucus vesicles |
| 18 June 2024 | ⚫ (Orange) | Male | 17 | None | No | Normal |
| 2 July 2024 | ⚫ (Green) | Female | 64 | None | No | Normal |

## Aerosol generation and characterization

To aerosolize oleoyl sarcosine, a 1 ml syringe (BD Plastipak, Becton Dickinson, Franklin Lakes, NJ USA) filled with 1 ml oleoyl sarcosine-ethanol (OS-EtOh, 10%) solution was positioned in a syringe pump (OEM pump model #78-2910, KD Scientific Inc., Holliston, MA, USA) to accurately continuously dose the solution to a collision atomizer (Model 3076, TSI, Inc., Shoreview, MN USA) connected to HEPA- and active-carbon filtered laboratory compressed air (2.5 bar). By varying the pump speed (0.5, 1, 3, 5, or 10 µL/min) different aerosol concentrations were generated. The aerosol flow (2.5 lpm) leaving the atomizer was guided into a stainless steel flow tube with four consecutive perpendicular sample outlets to connect to the exposure module (6/4 CF Stainless Steel Exposure Module, VITROCELL^®^ Systems, Waldkirch, Germany). Additional HEPA-filtered clean air (CA, 5 lpm) was mixed in the first part of the dispersion tube to dilute the generated concentration and obtain a well-mixed homogenous concentration across the full length of the tube. Each cell insert position (4x) extracted a limited airflow (3 mlpm per position) from this main aerosol flow to expose the air-liquid interface (ALI)-cultured lung cells.

Triton X-100 aerosols were generated using a glass collision atomizer (in-house built) containing a 250 ml solution (0.063%, 0.25%, 1%, 4.5%, 18%) of Triton X-100 with MilliQ water (Merck KGaA, Brand Millipore, Darmstadt, Germany) and HEPA- and active carbon-filtered laboratory compressed air (2 bar). The aerosol flow (0.5 lpm)) leaving the atomizer was guided into a stainless-steel flow tube as described above. Additional HEPA-filtered CA (7 lpm for 0.063%-4.5% TX-100 solutions, and 5.5 lpm for 18% TX-100 solution) was mixed in the first part of the dispersion tube to dilute the generated concentration and obtain a well-mixed homogenous concentration across the full length of the tube. Each cell insert position (4x) extracted a limited airflow (3 mlpm per position) from this main aerosol flow to expose the ALI-cultured lung cells.

The aerosol size distribution was determined using an Aerodynamic Particle Sizer (APS, Model 3321, TSI, Inc., Shoreview, MN USA). The instrumental sample inlet was connected to the second insert position inlet to measure the aerosol size distribution. The median mass aerodynamic diameter (MMAD) of the atomized 18% Triton X-100 particles was 2.8 µm. The aerosol size distributions of other Triton X-100 concentrations were not determined because all Triton X-100 concentrations were made in MilliQ water and similar pressure was used for atomization. The aerodynamic mass mode of the oleoyl sarcosine-ethanol concentration particles was 0.9 µm for all syringe speed settings.

## Aerosol exposure conditions and concentrations

For aerosol exposures in BEAS-2B cells, oleoyl sarcosine was diluted in 10% ethanol (due to high viscosity and water insolubility) and Triton X-100 was diluted in MilliQ water and an aerosol atmosphere was generated using a collision atomizer. CA was used as a negative control for aerosol exposures of Triton X-100 and MilliQ water with 10% ethanol was used as solvent control for oleoyl sarcosine exposures. The inserts were transferred to a VITROCELL^®^ 6/4 exposure module. The module was maintained at 37°C using a circulating water bath and each well was filled with 5.5 mL BEBM, allowing cells to be nourished from the basolateral side while being exposed at the ALI from the apical side. The distance between the inlet and insert (i.e., trumpet height) was 3 mm and the flow of the test atmospheres at the apical surface of the cells was generated using a vacuum pump at a constant rate of 3.0 mL/min. All exposures were carried out for 30 minutes (min) and the cells were exposed to five different concentrations of the two surfactants (See Supplementary Table 4). After aerosol exposure, inserts were placed in a new, sterile 24-well plate with 600 µL fresh BEBM added basolaterally. The cells were not washed apically after exposure. After 19-24 h in the incubator, 200 µL basolateral medium from the inserts was collected for assessment of cytotoxicity and the remaining was frozen and stored at -80°C for later assessment of cytokine secretion as described in the main part of this manuscript. The cells were used for the cell viability assay. At least three biologically independent experimental runs, using different cell passages, were performed for each exposure condition. After exposure, the VITROCELL^®^ exposure module and all tubing were thoroughly rinsed with 70% ethanol.

Dosimetry was performed by exposing stainless steel inserts filled with 125 µL DMSO for oleoyl sarcosine and 125 µL phosphate buffered saline for Triton X-100. DMSO was chosen as trapping liquid because oleoyl sarcosine is hydrophobic and is not well trapped in PBS. After generation, 100 µL was pipetted from the stainless steel insert and transferred to a glass vial for LC-MS/MS analysis. Two runs were analyzed and the measured versus nominal concentrations can be observed in Suppl Table 4.

**Supplementary Table 4.** Test concentrations used in BEAS-2B cells for aerosol exposures

|  | **Aerosol** | | | | |
| --- | --- | --- | --- | --- | --- |
|  | **Oleoyl sarcosine** | |  | **Triton X-100** | |
| **µL/min** | **Nominal concentration (µg/cm²)** | **Measured concentration (µg/cm²)** | **%** | **Nominal concentration (µg/cm²)** | **Measured concentration (µg/cm²)** |
| 0.5 | 0.6 | 0.02 +/- 0.01 | 0.0625 | 3 | 0.17 +/- 0.15 |
| 1 | 1.2 | 0.01 +/- 0.01 | 0.25 | 6 | 0.46 +/- 0.49 |
| 3 | 1.8 | 0.69 +/- 0.18 | 1 | 9 | 0.67 +/- 0.47 |
| 5 | 2.4 | 1.98 +/- 0.24 | 4.5 | 12 | 2.39 +/- 1.04 |
| 10 | 3 | 2.55 +/- 0.71 | 18 | 15 | 4.32 +/- 0.92 |

## Supplementary figures with results from aerosol exposure

**Supplementary Figure 1. Assessment of cell viability and cytotoxicity in BEAS-2B cell line after aerosol exposure.** BEAS-2B cells were exposed to five different concentrations of oleoyl sarcosine, Triton X-100, NO_2_, solvent control (SC) or clean air (CA) for 30 minutes at the air-liquid interface. After 24 hours, cell viability was assessed using PrestoBlue assay (A and B) and cytotoxicity (C and D) was assessed using lactate dehydrogenase (LDH) assay. The graphs show results from three separate experimental runs for the two surfactants represented in different colors (N=3), with each run having four replicates (n=4). The results were normalized to SC or CA, respectively (=100% cell viability and cytotoxicity). The numbers below the graph show the median response and the measured concentration of the test substance. The asterisk (*) shows statistical significance compared to SC or CA; *, *P*<0.05; **, *P* < 0.01; ***, *P* < 0.001.

**Supplementary Figure 2. Assessment of inflammatory marker secretion in BEAS-2B cell line after aerosol exposure.** BEAS-2B cells were exposed to five different concentrations of oleoyl sarcosine, Triton X-100 , NO_2_, solvent control (SC) or clean air (CA) for 30 minutes at the air-liquid interface. Samples from the basolateral medium were collected for assessment of inflammatory markers after 24 hours. The graphs show results from three separate experimental runs per condition (biological replicates; represented in different colors, N=3), with each run having four technical replicates (n=4). The numbers below the figures represent the median of secreted IL-6 and CXCL-8, and the measured concentrations of test substance. Abbreviations: IL, Interleukin; LPS, lipopolysacharide. The asterisk (*) shows statistical significance compared to SC and CA, respectively; *, *P*<0.05; **, *P* < 0.01; ***, *P* < 0.001.

## Exposure conditions and particle properties considered for multi-path particle dosimetry modelling

**Supplementary Table 5.** Exposure conditions and particle properties applied to the EPA MPPD v2.0 model for consumer exposures to Triton X-100.

|  | **Test Substance** | Triton X-100 (liquid hygroscopic) | | | |
| --- | --- | --- | --- | --- | --- |
|  | **Particle Sizes of Interest** | MMAD=2, 3, and 4 µm with GSD=2 | | | |
|  | **Particle Distribution Reference(s)** | (Lovén et al., 2019) | | | |
|  |  |  |  |  |  |
|  | **Population Considerations** | **Unit** | **MMAD=2, GSD=2** | **MMAD=3, GSD=2** | **MMAD=4, GSD=2** |
| Model Choice | Species |  | Human | Human | Human |
|  | Model/rationale |  | **PNNL Asymmetric** | **PNNL Asymmetric** | **PNNL Asymmetric** |
|  | Target Population |  | Adult Male | Adult Male | Adult Male |
|  | Function Residual Capacity (FRC) | ml | 3300 | 3300 | 3300 |
|  | Upper respiratory tract (URT) volume | ml | 50 | 50 | 50 |
|  |  |  |  |  |  |
|  | **Exposure Conditions and Activity** |  | **EPA General Default Breathing Rate: 20 m^3^/24 h** | | |
| Exposure conditions | Gravity | cm/s^2^ | 981 | 981 | 981 |
|  | Body Orientation |  | Upright | Upright | Upright |
|  | Aerosol concentration (BMCL) | mg/m³ | 1 | 1 | 1 |
|  | Breathing frequency | per min | **13.889** | **13.889** | **13.889** |
|  | Tidal volume | ml | **1000.0** | **1000.0** | **1000.0** |
|  | Inspiratory fraction | - | 0.5 | 0.5 | 0.5 |
|  | Pause fraction | - | 0 | 0 | 0 |
|  | Breathing scenario |  | **Oronasal-Normal Augmenter** | **Oronasal-Normal Augmenter** | **Oronasal-Normal Augmenter** |
|  |  |  |  |  |  |
|  | **Particle Properties** |  |  |  |  |
| Particle Properties | Density | g/cm³ | **1 (assume product is mostly water)** | **1 (assume product is mostly water)** | **1 (assume product is mostly water)** |
|  | Aspect Ratio | - | **1 (assume spherical)** | **1 (assume spherical)** | **1 (assume spherical)** |
|  | Diameter (MMAD) | µm | **2** | **3** | **4** |
|  | Particle Population Setting (single, multiple, etc) |  | Single | Single | Single |
|  | Inhalability Adjustment | - | Checked | Checked | Checked |
|  | GSD (diameter) | - | **2** | **2** | **2** |
|  | GSD (length) | - | 1.0 (default value) | 1.0 (default value) | 1.0 (default value) |
|  | Correlation | - | 0.0 (default value) | 0.0 (default value) | 0.0 (default value) |
|  | Type (liquid, solid, non-hygroscopic) | - | **Liquid hygroscopic** | **Liquid hygroscopic** | **Liquid hygroscopic** |
|  | Ambient RH | % | **50** | **50** | **50** |
|  | Mass fraction of soluble material (liquid only) |  | **25** | **25** | **25** |
|  | MW of soluble material (liquid only) | g/mol | **625** | **625** | **625** |
|  |  |  |  |  |  |
|  | Choice of Dose Metric | - | Deposition | Deposition | Deposition |
|  |  |  |  |  |  |
| MPPD Outputs | Total ET deposition fraction: |  | 0.5339 | 0.6531 | 0.7306 |
|  | Total TB deposition fraction: |  | 0.3259 | 0.2791 | 0.2218 |
|  | Total pulmonary deposition fraction: |  | 0.112 | 0.0497 | 0.0252 |
|  | Total LRT deposition fraction: |  | 0.4379 | 0.3288 | 0.247 |
|  | Total deposition fraction: |  | 0.9718 | 0.9819 | 0.9775 |
|  |  |  |  |  |  |
|  | ET deposited mass rate: | µg/min/cm² | 1.70E-02 | 2.08E-02 | 2.32E-02 |
|  | TB deposited mass rate: | µg/min/cm² | 9.75E-04 | 8.35E-04 | 6.64E-04 |
|  | Pulmonary deposited mass rate: | µg/min/cm² | 2.31E-06 | 1.02E-06 | 5.18E-07 |
|  | LRT deposited mass rate: | µg/min/cm² | 8.95E-06 | 6.72E-06 | 5.05E-06 |
|  | Total deposited mass rate: | µg/min/cm² | 1.99E-05 | 2.01E-05 | 2.00E-05 |
|  |  |  |  |  |  |
| Duration-Adjusted MPPD Outputs | Exposure Duration Adjustment | h | 24 | 24 | 24 |
|  | Exposure Duration Adjustment | min | 1440 | 1440 | 1440 |
|  |  |  |  |  |  |
|  | 24h ET deposition | µg/cm² | 2.45E+01 | 2.99E+01 | 3.35E+01 |
|  | 24h TB deposition | µg/cm² | 1.40E+00 | 1.20E+00 | 9.55E-01 |
|  | 24h Pulmonary deposition | µg/cm² | 3.32E-03 | 1.47E-03 | 7.46E-04 |
|  | 24h Total RT deposition | µg/cm² | 2.86E-02 | 2.89E-02 | 2.88E-02 |

**Supplementary Table 6.** Exposure conditions and particle properties applied to the EPA MPPD v2.0 model for consumer exposures to oleoyl sarcosine.

|  | **Test Substance** | Oleoyl Sarcosine (non-hygroscopic) | | | |
| --- | --- | --- | --- | --- | --- |
|  | **Particle Sizes of Interest** | MMAD=3, 6, and 8 µm with GSD=2.5 | | | |
|  | **Particle Distribution Reference(s)** | (Rosenthal & Yeagy, 2001; Sabty-Daily, Harris, et al., 2005; Sabty-Daily, Hinds, et al., 2005; Wang et al., 2005; Woskie et al., 1996) | | | |
|  |  |  |  |  |  |
|  | **Population Considerations** | **Unit** | **MMAD=3, GSD=2.5** | **MMAD=6, GSD=2.5** | **MMAD=8, GSD=2.5** |
| Model Choice | Species |  | Human | Human | Human |
|  | Model/rationale |  | **PNNL Asymmetric** | **PNNL Asymmetric** | **PNNL Asymmetric** |
|  | Target Population |  | Adult Male | Adult Male | Adult Male |
|  | Function Residual Capacity (FRC) | ml | 3300 | 3300 | 3300 |
|  | Upper respiratory tract (URT) volume | ml | 50 | 50 | 50 |
|  |  |  |  |  |  |
|  | **Exposure Conditions and Activity** |  | **EPA General Default Breathing Rate: 20 m^3^/24 h** | | |
| Exposure conditions | Gravity | cm/s^2^ | 981 | 981 | 981 |
|  | Body Orientation |  | Upright | Upright | Upright |
|  | Aerosol concentration (BMCL) | mg/m³ | 1 | 1 | 1 |
|  | Breathing frequency | per min | **13.889** | **13.889** | **13.889** |
|  | Tidal volume | ml | **1000.0** | **1000.0** | **1000.0** |
|  | Inspiratory fraction | - | 0.5 | 0.5 | 0.5 |
|  | Pause fraction | - | 0 | 0 | 0 |
|  | Breathing scenario |  | **Oronasal-Normal Augmenter** | **Oronasal-Normal Augmenter** | **Oronasal-Normal Augmenter** |
|  |  |  |  |  |  |
|  | **Particle Properties** |  |  |  |  |
| Particle Properties | Density | g/cm³ | **1 (assume product is mostly water)** | **1 (assume product is mostly water)** | **1 (assume product is mostly water)** |
|  | Aspect Ratio | - | **1 (assume spherical)** | **1 (assume spherical)** | **1 (assume spherical)** |
|  | Diameter (MMAD) | µm | **3** | **6** | **8** |
|  | Particle Population Setting (single, multiple, etc) |  | Single | Single | Single |
|  | Inhalability Adjustment | - | Checked | Checked | Checked |
|  | GSD (diameter) | - | **2.5** | **2.5** | **2.5** |
|  | GSD (length) | - | 1.0 (default value) | 1.0 (default value) | 1.0 (default value) |
|  | Correlation | - | 0.0 (default value) | 0.0 (default value) | 0.0 (default value) |
|  | Type (liquid, solid, non-hygroscopic) | - | **Non-hygroscopic** | **Non-hygroscopic** | **Non-hygroscopic** |
|  | Ambient RH | % | n/a | n/a | n/a |
|  | Mass fraction of soluble material (liquid only) |  | n/a | n/a | n/a |
|  | MW of soluble material (liquid only) | g/mol | n/a | n/a | n/a |
|  |  |  |  |  |  |
|  | Choice of Dose Metric | - | Deposition | Deposition | Deposition |
|  |  |  |  |  |  |
| MPPD Outputs | Total ET deposition fraction: |  | 0.5966 | 0.7213 | 0.7344 |
|  | Total TB deposition fraction: |  | 0.0841 | 0.0729 | 0.0725 |
|  | Total pulmonary deposition fraction: |  | 0.0939 | 0.0698 | 0.0379 |
|  | Total LRT deposition fraction: |  | 0.178 | 0.1427 | 0.1103 |
|  | Total deposition fraction: |  | 0.7746 | 0.8641 | 0.8447 |
|  |  |  |  |  |  |
|  | ET deposited mass rate: | µg/min/cm² | 1.90E-02 | 2.29E-02 | 2.34E-02 |
|  | TB deposited mass rate: | µg/min/cm² | 2.52E-04 | 2.18E-04 | 2.17E-04 |
|  | Pulmonary deposited mass rate: | µg/min/cm² | 1.93E-06 | 1.44E-06 | 7.79E-07 |
|  | LRT deposited mass rate: | µg/min/cm² | 3.64E-06 | 2.92E-06 | 2.26E-06 |
|  | Total deposited mass rate: | µg/min/cm² | 1.58E-05 | 1.77E-05 | 1.73E-05 |
|  |  |  |  |  |  |
| Duration-Adjusted MPPD Outputs | Exposure Duration Adjustment | h | 24 | 24 | 24 |
|  | Exposure Duration Adjustment | min | 1440 | 1440 | 1440 |
|  |  |  |  |  |  |
|  | 24h ET deposition | µg/cm² | 2.73E+01 | 3.30E+01 | 3.36E+01 |
|  | 24h TB deposition | µg/cm² | 3.62E-01 | 3.14E-01 | 3.12E-01 |
|  | 24h Pulmonary deposition | µg/cm² | 2.78E-03 | 2.07E-03 | 1.12E-03 |
|  | 24h Total RT deposition | µg/cm² | 2.28E-02 | 2.54E-02 | 2.49E-02 |

**Supplementary Table 7.** Exposure conditions and particle properties applied to the EPA MPPD v2.0 model for occupational exposures to Triton X-100.

|  | **Test Substance** | Triton X-100 (liquid hygroscopic) | | | |
| --- | --- | --- | --- | --- | --- |
|  | **Particle Sizes of Interest** | MMAD=2, 3, and 4 µm with GSD=2 | | | |
|  | **Particle Distribution Reference(s)** | (Lovén et al., 2019) | | | |
|  | | | | | |
|  | **Population Considerations** | **Unit** | **MMAD=2, GSD=2** | **MMAD=3, GSD=2** | **MMAD=4, GSD=2** |
| Model Choice | Species |  | Human | Human | Human |
|  | Model/rationale |  | **PNNL Asymmetric** | **PNNL Asymmetric** | **PNNL Asymmetric** |
|  | Target Population |  | Adult Male | Adult Male | Adult Male |
|  | Function Residual Capacity (FRC) | ml | 3300 | 3300 | 3300 |
|  | Upper respiratory tract (URT) volume | ml | 50 | 50 | 50 |
|  |  |  |  |  |  |
|  | **Exposure Conditions and Activity** |  | **EPA Occupational Breathing Rate: 10 m^3^/8 h** | | |
| Exposure conditions | Gravity | cm/s^2^ | 981 | 981 | 981 |
|  | Body Orientation |  | Upright | Upright | Upright |
|  | Aerosol concentration (BMCL) | mg/m³ | 1 | 1 | 1 |
|  | Breathing frequency | per min | **18.257** | **18.257** | **18.257** |
|  | Tidal volume | ml | **1141.089** | **1141.089** | **1141.089** |
|  | Inspiratory fraction | - | 0.5 | 0.5 | 0.5 |
|  | Pause fraction | - | 0 | 0 | 0 |
|  | Breathing scenario |  | **Oronasal-Normal Augmenter** | **Oronasal-Normal Augmenter** | **Oronasal-Normal Augmenter** |
|  |  |  |  |  |  |
|  | **Particle Properties** |  |  |  |  |
| Particle Properties | Density | g/cm³ | **1 (assume product is mostly water)** | **1 (assume product is mostly water)** | **1 (assume product is mostly water)** |
|  | Aspect Ratio | - | **1 (assume spherical)** | **1 (assume spherical)** | **1 (assume spherical)** |
|  | Diameter (MMAD) | µm | **2** | **3** | **4** |
|  | Particle Population Setting (single, multiple, etc) |  | Single | Single | Single |
|  | Inhalability Adjustment | - | Checked | Checked | Checked |
|  | GSD (diameter) | - | **2** | **2** | **2** |
|  | GSD (length) | - | 1.0 (default value) | 1.0 (default value) | 1.0 (default value) |
|  | Correlation | - | 0.0 (default value) | 0.0 (default value) | 0.0 (default value) |
|  | Type (liquid, solid, non-hygroscopic) | - | **Liquid hygroscopic** | **Liquid hygroscopic** | **Liquid hygroscopic** |
|  | Ambient RH | % | **50** | **50** | **50** |
|  | Mass fraction of soluble material (liquid only) |  | **25** | **25** | **25** |
|  | MW of soluble material (liquid only) | g/mol | **625** | **625** | **625** |
|  |  |  |  |  |  |
|  | Choice of Dose Metric | - | Deposition | Deposition | Deposition |
|  |  |  |  |  |  |
| MPPD Outputs | Total ET deposition fraction: |  | 0.5956 | 0.7104 | 0.7803 |
|  | Total TB deposition fraction: |  | 0.3107 | 0.2453 | 0.1837 |
|  | Total pulmonary deposition fraction: |  | 0.0716 | 0.0289 | 0.015 |
|  | Total LRT deposition fraction: |  | 0.3822 | 0.2742 | 0.1987 |
|  | Total deposition fraction: |  | 0.9778 | 0.9847 | 0.979 |
|  |  |  |  |  |  |
|  | ET deposited mass rate: | µg/min/cm² | 2.84E-02 | 3.39E-02 | 3.72E-02 |
|  | TB deposited mass rate: | µg/min/cm² | 1.39E-03 | 1.10E-03 | 8.24E-04 |
|  | Pulmonary deposited mass rate: | µg/min/cm² | 2.21E-06 | 8.93E-07 | 4.62E-07 |
|  | LRT deposited mass rate: | µg/min/cm² | 1.17E-05 | 8.41E-06 | 6.09E-06 |
|  | Total deposited mass rate: | µg/min/cm² | 3.00E-05 | 3.02E-05 | 3.00E-05 |
|  |  |  |  |  |  |
| Duration-Adjusted MPPD Outputs | Exposure Duration Adjustment | h | 8 | 8 | 8 |
|  | Exposure Duration Adjustment | min | 480 | 480 | 480 |
|  |  |  |  |  |  |
|  | 8h ET deposition | µg/cm² | 1.36E+01 | 1.63E+01 | 1.79E+01 |
|  | 8h TB deposition | µg/cm² | 6.69E-01 | 5.28E-01 | 3.96E-01 |
|  | 8h Pulmonary deposition | µg/cm² | 1.06E-03 | 4.28E-04 | 2.22E-04 |
|  | 8h Total RT deposition | µg/cm² | 1.44E-02 | 1.45E-02 | 1.44E-02 |

**Supplementary Table 8.** Exposure conditions and particle properties applied to the EPA MPPD v2.0 model for occupational exposures to oleoyl sarcosine.

|  | **Test Substance** | Oleoyl Sarcosine (non-hygroscopic) | | | |
| --- | --- | --- | --- | --- | --- |
|  | **Particle Sizes of Interest** | MMAD=3, 6, and 8 µm with GSD=2.5 | | | |
|  | **Particle Distribution Reference(s)** | (Rosenthal & Yeagy, 2001; Sabty-Daily, Harris, et al., 2005; Sabty-Daily, Hinds, et al., 2005; Wang et al., 2005; Woskie et al., 1996) | | | |
|  |  |  |  |  |  |
|  | **Population Considerations** | **Unit** | **MMAD=3, GSD=2.5** | **MMAD=6, GSD=2.5** | **MMAD=8, GSD=2.5** |
| Model Choice | Species |  | Human | Human | Human |
|  | Model/rationale |  | **PNNL Asymmetric** | **PNNL Asymmetric** | **PNNL Asymmetric** |
|  | Target Population |  | Adult Male | Adult Male | Adult Male |
|  | Function Residual Capacity (FRC) | ml | **3300** | **3300** | **3300** |
|  | Upper respiratory tract (URT) volume | ml | **50** | **50** | **50** |
|  |  |  |  |  |  |
|  | **Exposure Conditions and Activity** |  | **EPA Occupational Breathing Rate: 10 m^3^/8 h** | | |
| Exposure conditions | Gravity | cm/s^2^ | 981 | 981 | 981 |
|  | Body Orientation |  | Upright | Upright | Upright |
|  | Aerosol concentration (BMCL) | mg/m³ | 1 | 1 | 1 |
|  | Breathing frequency | per min | **18.257** | **18.257** | **18.257** |
|  | Tidal volume | ml | **1141.089** | **1141.089** | **1141.089** |
|  | Inspiratory fraction | - | 0.5 | 0.5 | 0.5 |
|  | Pause fraction | - | 0 | 0 | 0 |
|  | Breathing scenario |  | **Oronasal-Normal Augmenter** | **Oronasal-Normal Augmenter** | **Oronasal-Normal Augmenter** |
|  |  |  |  |  |  |
|  | **Particle Properties** |  |  |  |  |
| Particle Properties | Density | g/cm³ | **1 (assume product is mostly water)** | **1 (assume product is mostly water)** | **1 (assume product is mostly water)** |
|  | Aspect Ratio | - | **1 (assume spherical)** | **1 (assume spherical)** | **1 (assume spherical)** |
|  | Diameter (MMAD) | µm | **MMAD** | **MMAD** | **MMAD** |
|  | Particle Population Setting (single, multiple, etc) |  | **3** | **6** | **8** |
|  | Inhalability Adjustment | - | Single | Single | Single |
|  | GSD (diameter) | - | Checked | Checked | Checked |
|  | GSD (length) | - | **2.5** | **2.5** | **2.5** |
|  | Correlation | - | 1.0 (default value) | 1.0 (default value) | 1.0 (default value) |
|  | Type (liquid, solid, non-hygroscopic) | - | 0.0 (default value) | 0.0 (default value) | 0.0 (default value) |
|  | Ambient RH | % | **Non-hygroscopic** | **Non-hygroscopic** | **Non-hygroscopic** |
|  | Mass fraction of soluble material (liquid only) |  | n/a | n/a | n/a |
|  | MW of soluble material (liquid only) | g/mol | n/a | n/a | n/a |
|  |  |  |  |  |  |
|  | Choice of Dose Metric | - | Deposition | Deposition | Deposition |
|  |  |  |  |  |  |
| MPPD Outputs | Total ET deposition fraction: |  | 0.6634 | 0.763 | 0.7681 |
|  | Total TB deposition fraction: |  | 0.0743 | 0.0769 | 0.0664 |
|  | Total pulmonary deposition fraction: |  | 0.0667 | 0.0379 | 0.0173 |
|  | Total LRT deposition fraction: |  | 0.141 | 0.1148 | 0.0836 |
|  | Total deposition fraction: |  | 0.8044 | 0.8778 | 0.8517 |
|  |  |  |  |  |  |
|  | ET deposited mass rate: | µg/min/cm² | 3.17E-02 | 3.64E-02 | 3.66E-02 |
|  | TB deposited mass rate: | µg/min/cm² | 3.33E-04 | 3.45E-04 | 2.98E-04 |
|  | Pulmonary deposited mass rate: | µg/min/cm² | 2.06E-06 | 1.17E-06 | 5.33E-07 |
|  | LRT deposited mass rate: | µg/min/cm² | 4.32E-06 | 3.52E-06 | 2.57E-06 |
|  | Total deposited mass rate: | µg/min/cm² | 2.47E-05 | 2.69E-05 | 2.61E-05 |
|  |  |  |  |  |  |
| Duration-Adjusted MPPD Outputs | Exposure Duration Adjustment | h | 8 | 8 | 8 |
|  | Exposure Duration Adjustment | min | 480 | 480 | 480 |
|  |  |  |  |  |  |
|  | 8h ET deposition | µg/cm² | 1.52E+01 | 1.75E+01 | 1.76E+01 |
|  | 8h TB deposition | µg/cm² | 1.60E-01 | 1.66E-01 | 1.43E-01 |
|  | 8h Pulmonary deposition | µg/cm² | 9.89E-04 | 5.63E-04 | 2.56E-04 |
|  | 8h Total RT deposition | µg/cm² | 1.18E-02 | 1.29E-02 | 1.25E-02 |

## Assessment of skin and eye irritation potential of the two surfactants

Skin and eye irritation testing (non-GLP) of oleoyl sarcosine and Triton X-100 was assessed according to Organisation for Economic Co-operation and Development (OECD) Test Guideline (TG) 431: In vitro skin corrosion: reconstructed human epidermis (RHE) test method, OECD TG 439: In Vitro Skin Irritation: Reconstructed Human Epidermis Test Method using SkinEthic RHE models, and OECD TG 492B: Reconstructed Human Cornea-like Epithelium (RHCE) Test Method for Eye Hazard Identification using SkinEthic HCE models, respectively.

To predict the potential of both surfactants to cause skin irritation and corrosion, a top-down approach was applied. According to OECD TG 431, both chemicals were non-corrosive and then predicted to be GHS Category 2 skin irritants based on OECD TG 439. Additionally, according to OECD TG 492B, Triton X-100 and oleoyl sarcosine are categorized as GHS Category 1 (serious eye damage) and GHS Category 2 (eye irritation), respectively. (See Supplementary Table 9).

**Supplementary Table 9:** Results from skin and eye irritation OECD test guidelines (TG).

| **Chemicals** | **TG 431** | | | **TG 439** | | **TG492B** | | | |
| --- | --- | --- | --- | --- | --- | --- | --- | --- | --- |
|  | **% viability ± SD** | | **CL** | **% viability ± SD** | **CL** | **% viability ± SD** | | | **CL** |
|  | *3 min* | *60 min* |  |  |  | *5 min* | *16 min* | *120 min* |  |
| **Oleoyl sarcosine** | 103.10 ± 7.34 | 95.96 ± 7.16 | Non-corrosive | 2.19 ± 0.14 | Cat 2 | 20.4 ± 7.16 | 56.1 ± 4.11 | 2.7 ± 0.065 | Cat 2 |
| **Triton X-100** | 64.08 ± 1.80 | 56.29 ± 2.80 | Non-corrosive | 1.49 ± 0.35 | Cat 2 | 28.0 ± 3.32 | 1.7 ± 0.07 | 0.5 ± 0.06 | Cat 1 |

# Benchmark dose (BMD) modelling results

## BEAS-2B: Oleoyl sarcosine, 24 h

### Dataset Name: BEAS-2B Oleoyl sarcosine, IL-6 (pg/ml)

| Dose (µg/cm²) (µg/cm²) | N | Mean (pg/ml) | Std. Dev. (pg/ml) |
| --- | --- | --- | --- |
| 0 | 3 | 0.399 | 0.153 |
| 0.5 | 3 | 2.637 | 0.393 |
| 1.1 | 3 | 31.499 | 10.43 |
| 1.6 | 3 | 24.413 | 12.849 |

Test 1 Dose response: < 0.0001

Test 2 Homogeneity of Variance: < 0.0001

Test 3 Variance Model Selection: 0.190

| **BMR Type** | Std. Dev. |
| --- | --- |
| **BMRF** | 1 |
| **Distribution Type** | Normal + Non-constant |
| **Adverse Direction** | Automatic |
| **Maximum Polynomial Degree** | 3 |
| **Confidence Level (one sided)** | 0.95 |

Maximum Likelihood Approach Model Results

| **Model** | **BMDL (µg/cm²)** | **BMD (µg/cm²)** | **BMDU (µg/cm²)** | ***P*-Value** | **AIC** | **Scaled Residual at Control** | **Scaled Residual near BMD** | **Recommendation and Notes** |
| --- | --- | --- | --- | --- | --- | --- | --- | --- |
| **Restricted Models** | | | | | | | | |
| Exponential 3 | 0.04 | 0.063 | 0.064 | < 0.0001 | 72.187 | -0.356 | -0.356 | Questionable |
| Exponential 5 | 0.207 | 0.361 | 0.445 | - | 57.456 | -0.201 | 0.391 | Questionable |
| Hill*† | 0.241 | 0.422 | 0.443 | 0.516 | 55.456 | -0.201 | 0.391 | Recommended - Lowest AIC |
| Polynomial 2 | 0.023 | 0.093 | 0.14 | 0.000677 | 67.631 | 0.03 | 0.03 | Questionable |
| Polynomial 3 | 0.034 | 0.18 | 0.242 | 0.000717 | 66.479 | -0.223 | -0.223 | Questionable |
| Power | 0.098 | 0.173 | 0.25 | 0.000763 | 66.364 | -0.194 | -0.194 | Questionable |
| **Unrestricted Models** | | | | | | | | |
| Linear | 0.006 | 0.011 | 0.026 | < 0.0001 | 73.59 | -0.013 | -0.013 | Questionable |
| * BMDS recommended best fitting model  † User selected best fitting model | | | | | | | | |

### Dataset Name: BEAS-2B Oleoyl sarcosine, IL-8 (pg/mL)

| Dose (µg/cm²) | N | Mean | Std. Dev. |
| --- | --- | --- | --- |
| 0 | 3 | 50.106 | 26.122 |
| 0.5 | 3 | 95.035 | 43.223 |
| 1.1 | 3 | 213.308 | 112.207 |
| 1.6 | 3 | 145.445 | 98.607 |
| Test 1 Dose response: 0.0255  Test 2 Homogeneity of Variance: 0.0794  Test 3 Variance Model Selection: 0.0794 | | | |

| **BMR Type** | Std. Dev. |
| --- | --- |
| **BMRF** | 1 |
| **Distribution Type** | Normal + Constant |
| **Adverse Direction** | Automatic |
| **Maximum Polynomial Degree** | 3 |
| **Confidence Level (one sided)** | 0.95 |

Maximum Likelihood Approach Model Results

| **Model** | **BMDL (µg/cm²)** | **BMD (µg/cm²)** | **BMDU (µg/cm²)** | ***P*-Value** | **AIC** | **Scaled Residual at Control** | **Scaled Residual near BMD** | **Recommendation and Notes** |
| --- | --- | --- | --- | --- | --- | --- | --- | --- |
| **Restricted Models** | | | | | | | | |
| Exponential 3 | 0.894 | 1.328 | 13.824 | 0.212 | 144.516 | -0.69 | 1.603 | Viable |
| Exponential 5 | 0.514 | 0.558 | 0.636 | - | 145.569 | -1.46e-8 | 5.39e-9 | Questionable |
| Hill | 4.06e-4 | 0.521 | - | 0.212 | 143.569 | 2.16e-6 | -4.78e-6 | Questionable |
| Polynomial 2 | 0.533 | 0.982 | 6.192 | 0.0514 | 145.803 | -0.332 | 1.477 | Questionable |
| Polynomial 3 | 0.533 | 0.98 | 6.192 | 0.150 | 143.803 | -0.328 | 1.476 | Viable |
| Power | 0.533 | 0.985 | 6.192 | 0.150 | 143.803 | -0.336 | 1.479 | Viable |
| **Unrestricted Models** | | | | | | | | |
| Linear*† | 0.533 | 0.985 | 6.192 | 0.150 | 143.803 | -0.336 | 1.479 | Recommended - Lowest AIC |
| * BMDS recommended best fitting model  † User selected best fitting model | | | | | | | | |

### Dataset Name: BEAS-2B Oleoyl sarcosine, Viability (rel. vehicle)

| Dose (µg/cm²) | N | Mean | Std. Dev. |
| --- | --- | --- | --- |
| 0 | 3 | 1 | 0.146 |
| 0.5 | 3 | 0.879 | 0.112 |
| 1.1 | 3 | 0.598 | 0.069 |
| 1.6 | 3 | 0.169 | 0.051 |
| 2.4 | 3 | 0.058 | 0.007 |
| 3.1 | 3 | 0.034 | 0.019 |
| Test 1 Dose response: < 0.0001  Test 2 Homogeneity of Variance: 0.000392  Test 3 Variance Model Selection: 0.284 | | | |

| **BMR Type** | Std. Dev. |
| --- | --- |
| **BMRF** | 1 |
| **Distribution Type** | Normal + Non-constant |
| **Adverse Direction** | Automatic |
| **Maximum Polynomial Degree** | 3 |
| **Confidence Level (one sided)** | 0.95 |

Maximum Likelihood Approach Model Results

| **Model** | **BMDL (µg/cm²)** | **BMD (µg/cm²)** | **BMDU (µg/cm²)** | ***P*-Value** | **AIC** | **Scaled Residual at Control** | **Scaled Residual near BMD** | **Recommendation and Notes** |  |
| --- | --- | --- | --- | --- | --- | --- | --- | --- | --- |
| **Restricted Models** | | | | | | | | | |
| Exponential 3 | 0.3 | 0.442 | 0.813 | 0.00130 | -35.436 | -0.305 | 0.018 | Questionable |  |
| Exponential 5 | 0.547 | 0.731 | 0.94 | 0.144 | -47.439 | 0.634 | -0.831 | Viable |  |
| Hill*† | 0.711 | 0.834 | 0.979 | 0.312 | -48.987 | 0.808 | 0.296 | Recommended - Lowest AIC |  |
| Polynomial 2 | 0.346 | 1.005 | 2.565 | <0.0001 | -12.999 | 1.185 | 0.525 | Questionable |  |
| Polynomial 3 | 0.346 | 1.007 | 2.565 | <0.0001 | -12.999 | 1.186 | 0.526 | Questionable |  |
| Power | 0.477 | 1.113 | 2.452 | <0.0001 | -12.965 | 1.273 | 0.639 | Questionable |  |
| **Unrestricted Models** | | | | | | | | | |
| Linear | 0.346 | 1.007 | 1.286 | <0.0001 | -12.999 | 1.186 | 0.526 | Questionable |  |
| * BMDS recommended best fitting model  † User selected best fitting model | | | | | | | | | |

### Dataset Name: BEAS-2B Oleoyl sarcosine, LDH (rel. vehicle)

| Dose (µg/cm²) | N | Mean | Std. Dev. |
| --- | --- | --- | --- |
| 0 | 3 | 1 | 0.241 |
| 0.5 | 3 | 1.191 | 0.178 |
| 1.1 | 3 | 1.201 | 0.32 |
| 1.6 | 3 | 2.345 | 0.179 |
| 2.4 | 3 | 3.059 | 0.291 |
| 3.1 | 3 | 3.352 | 0.038 |
| Test 1 Dose response: < 0.0001  Test 2 Homogeneity of Variance: 0.0821  Test 3 Variance Model Selection: 0.0821 | | | |

| **BMR Type** | Std. Dev. |
| --- | --- |
| **BMRF** | 1 |
| **Distribution Type** | Normal + Constant |
| **Adverse Direction** | Automatic |
| **Maximum Polynomial Degree** | 3 |
| **Confidence Level (one sided)** | 0.95 |

Maximum Likelihood Approach Model Results

| **Model** | **BMDL (µg/cm²)** | **BMD (µg/cm²)** | **BMDU (µg/cm²)** | ***P*-Value** | **AIC** | **Scaled Residual at Control** | **Scaled Residual near BMD** | **Recommendation and Notes** |  |
| --- | --- | --- | --- | --- | --- | --- | --- | --- | --- |
| **Restricted Models** | | | | | | | | | |
| Exponential 3 | 0.566 | 0.708 | 0.723 | 0.000525 | 18.462 | -0.255 | -0.46 | Questionable |  |
| Exponential 5 | 0.942 | 1.208 | 1.466 | 0.0974 | 5.128 | -0.779 | -0.006 | Questionable |  |
| Hill*† | 0.923 | 1.125 | 1.43 | 0.159 | 4.144 | -0.64 | -0.44 | Recommended - Lowest AIC |  |
| Polynomial 2 | 0.296 | 0.46 | 0.891 | 0.000253 | 17.635 | 0.73 | -0.112 | Questionable |  |
| Polynomial 3 | 0.296 | 0.456 | 0.892 | 0.000253 | 17.636 | 0.756 | -0.106 | Questionable |  |
| Power | 0.311 | 0.546 | 0.934 | 0.000378 | 16.788 | 0.516 | 0.033 | Questionable |  |
| **Unrestricted Models** | | | | | | | | | |
| Linear | 0.286 | 0.377 | 0.535 | 0.000545 | 16.277 | 1.151 | -0.12 | Questionable |  |
| * BMDS recommended best fitting model  † User selected best fitting model | | | | | | | | | |

## MucilAir: Oleoyl sarcosine, 24 h

### Dataset Name: MucilAir Oleoyl sarcosine, IL-6 (fold-change rel. vehicle)

| Dose (µg/cm²) | N | Mean | Std. Dev. |
| --- | --- | --- | --- |
| 0 | 3 | 1 | 0.585 |
| 5.2 | 3 | 2.35 | 1.119 |
| 10.3 | 3 | 33.604 | 36.135 |
| 20.1 | 3 | 70.015 | 74.449 |
| Test 1 Dose response: < 0.0001  Test 2 Homogeneity of Variance: < 0.0001  Test 3 Variance Model Selection: < 0.0001 | | | |

| **BMR Type** | Std. Dev. |
| --- | --- |
| **BMRF** | 1 |
| **Distribution Type** | Normal + Constant |
| **Adverse Direction** | Automatic |
| **Maximum Polynomial Degree** | 3 |
| **Confidence Level (one sided)** | 0.95 |

Maximum Likelihood Approach Model Results

| **Model** | **BMDL (µg/cm²)** | **BMD (µg/cm²)** | **BMDU (µg/cm²)** | ***P*-Value** | **AIC** | **Scaled Residual at Control** | **Scaled Residual near BMD** | **Recommendation and Notes** |
| --- | --- | --- | --- | --- | --- | --- | --- | --- |
| **Restricted Models** | | | | | | | | |
| Exponential 3 | 12.355 | 15.492 | 27.192 | 0.720 | 125.195 | -0.275 | -0.069 | Questionable |
| Exponential 5 | 8.843 | 12.006 | 20.366 | - | 128.809 | 0.05 | 0.311 | Questionable |
| Hill | 3.822 | 10.426 | - | - | 128.537 | -3.86e-7 | -7.60e-8 | Questionable |
| Polynomial 2 | 5.577 | 11.602 | 27.615 | 0.579 | 126.846 | 0.153 | 0.321 | Questionable |
| Polynomial 3 | 5.577 | 11.602 | 27.615 | 0.579 | 126.846 | 0.153 | 0.321 | Questionable |
| Power | 5.643 | 11.511 | 27.139 | 0.609 | 126.8 | 0.139 | 0.303 | Questionable |
| **Unrestricted Models** | | | | | | | | |
| Linear* | 5.517 | 9.32 | 29.104 | 0.801 | 124.982 | 0.359 | 0.085 | Questionable |
| * User selected best fitting model | | | | | | | | |

### Dataset Name: MucilAir Oleoyl sarcosine, IL-8 (pg/ml)

| Dose (µg/cm²) | N | Mean | Std. Dev. |
| --- | --- | --- | --- |
| 0 | 3 | 6204.131 | 2443.323 |
| 5.2 | 3 | 9497.087 | 1393.243 |
| 10.3 | 3 | 11940.251 | 719.192 |
| 20.1 | 3 | 13733.658 | 3172.682 |
| Test 1 Dose response: 0.00155  Test 2 Homogeneity of Variance: 0.108  Test 3 Variance Model Selection: 0.108 | | | |

| **BMR Type** | Std. Dev. |
| --- | --- |
| **BMRF** | 1 |
| **Distribution Type** | Normal + Constant |
| **Adverse Direction** | Automatic |
| **Maximum Polynomial Degree** | 3 |
| **Confidence Level (one sided)** | 0.95 |

Maximum Likelihood Approach Model Results

| **Model** | **BMDL (µg/cm²)** | **BMD (µg/cm²)** | **BMDU (µg/cm²)** | ***P*-Value** | **AIC** | **Scaled Residual at Control** | **Scaled Residual near BMD** | **Recommendation and Notes** | |
| --- | --- | --- | --- | --- | --- | --- | --- | --- | --- |
| **Restricted Models** | | | | | | | | |  |
| Exponential 3 | 0 | 7.59 | - | 0.148 | 223.167 | -1.186 | 0.444 | Unusable | |
| Exponential 5 | 1.129 | 2.865 | 7.022 | - | 223.349 | -3.60e-8 | 3.33e-8 | Questionable | |
| Hill | 0 | 3.063 | 4.146 | - | 223.349 | 4.11e-8 | -6.47e-8 | Unusable | |
| Polynomial 2 | 4.79 | 4.891 | 5.001 | 0.00894 | 228.184 | 0.391 | 0.749 | Questionable | |
| Polynomial 3 | 6.268 | 6.4 | 7.722 | 0.0274 | 226.541 | 0.079 | 0.525 | Questionable | |
| Power | 3.583 | 5.354 | - | 0.303 | 221.737 | -0.825 | 0.436 | Viable | |
| **Unrestricted Models** | | | | | | | | |  |
| Linear*† | 3.592 | 5.33 | 8.912 | 0.303 | 221.736 | -0.806 | 0.445 | Recommended - Lowest AIC | |
| * BMDS recommended best fitting model  † User selected best fitting model | | | | | | | | |  |

### Dataset Name: MucilAir Oleoyl sarcosine, Viablity (% vehicle)

| Dose (µg/cm²) | N | Mean | Std. Dev. |
| --- | --- | --- | --- |
| 0 | 3 | 1 | 0.044 |
| 5.2 | 3 | 1.009 | 0.206 |
| 10.3 | 3 | 0.87 | 0.156 |
| 20.1 | 3 | 0.94 | 0.116 |
| 41.1 | 3 | 0.249 | 0.175 |
| 77.8 | 3 | 0.041 | 0.037 |
| Test 1 Dose response: < 0.0001  Test 2 Homogeneity of Variance: 0.0472  Test 3 Variance Model Selection: 0.0635 | | | |

| **BMR Type** | Std. Dev. |
| --- | --- |
| **BMRF** | 1 |
| **Distribution Type** | Normal + Non-constant |
| **Adverse Direction** | Automatic |
| **Maximum Polynomial Degree** | 3 |
| **Confidence Level (one sided)** | 0.95 |

**Maximum Likelihood Approach Model Results**

| **Model** | **BMDL (µg/cm²)** | **BMD (µg/cm²)** | **BMDU (µg/cm²)** | ***P*-Value** | **AIC** | **Scaled Residual at Control** | **Scaled Residual near BMD** | **Recommendation and Notes** |
| --- | --- | --- | --- | --- | --- | --- | --- | --- |
| **Restricted Models** | | | | | | | | |
| Exponential 3*† | 17.182 | 26.99 | 28.982 | 0.669 | -17.477 | 0.488 | 0.095 | Recommended - Lowest AIC |
| Exponential 5 | 17.01 | 27.494 | 37.856 | 0.398 | -16.001 | 0.464 | 0.114 | Viable |
| Hill | 18.005 | 26.767 | 36.855 | 0.396 | -15.991 | 0.477 | 0.08 | Viable |
| Polynomial 2 | 10.234 | 15.069 | 21.99 | 0.00559 | -7.244 | -0.256 | -0.283 | Questionable |
| Polynomial 3 | 10.308 | 17.139 | 29.991 | 0.00994 | -8.553 | -0.061 | 1.683 | Questionable |
| Power | 11.047 | 17.117 | 31.927 | 0.00996 | -8.558 | -0.112 | 1.638 | Questionable |
| **Unrestricted Models** | | | | | | | | |
| Linear | 9.997 | 16.643 | 27.402 | 0.0100 | -8.573 | -0.137 | 1.644 | Questionable |
| * BMDS recommended best fitting model  † User selected best fitting model | | | | | | | | |

### Dataset Name: MucilAir Oleoyl sarcosine, LDH (fold change rel. vehicle)

| Dose (µg/cm²) | N | Mean | Std. Dev. |
| --- | --- | --- | --- |
| 0 | 3 | 1 | 0.411 |
| 5.2 | 3 | 1.057 | 0.264 |
| 10.3 | 3 | 2.415 | 1.251 |
| 20.1 | 3 | 17.538 | 8.316 |
| 41.1 | 3 | 38.89 | 11.485 |
| 77.8 | 3 | 52.237 | 14.826 |
| Test 1 Dose response: < 0.0001  Test 2 Homogeneity of Variance: < 0.0001  Test 3 Variance Model Selection: 0.711 | | | |

| **BMR Type** | Std. Dev. |
| --- | --- |
| **BMRF** | 1 |
| **Distribution Type** | Normal + Non-constant |
| **Adverse Direction** | Automatic |
| **Maximum Polynomial Degree** | 3 |
| **Confidence Level (one sided)** | 0.95 |

Maximum Likelihood Approach Model Results

| **Model** | **BMDL (µg/cm²)** | **BMD (µg/cm²)** | **BMDU (µg/cm²)** | ***P*-Value** | **AIC** | **Scaled Residual at Control** | **Scaled Residual near BMD** | **Recommendation and Notes** |  |
| --- | --- | --- | --- | --- | --- | --- | --- | --- | --- |
| **Restricted Models** | | | | | | | | | |
| Exponential 3 | 6.327 | 12.926 | 25.661 | <0.0001 | 138.525 | -1.025 | -0.745 | Questionable |  |
| Exponential 5 | 5.19 | 6.889 | 8.998 | 0.398 | 91.315 | 0.232 | -0.026 | Viable |  |
| Hill*† | 5.288 | 6.963 | 8.949 | 0.569 | 90.601 | 0.234 | 0.024 | Recommended - Lowest AIC |  |
| Polynomial 2 | 0.881 | 1.437 | 1.744 | <0.0001 | 120.292 | 0.321 | 0.321 | Questionable |  |
| Polynomial 3 | 0.881 | 1.437 | 1.744 | <0.0001 | 120.292 | 0.321 | 0.321 | Questionable |  |
| Power | 1.35 | 3.055 | 5.435 | <0.0001 | 112.597 | 0.891 | -1.274 | Questionable |  |
| **Unrestricted Models** | | | | | | | | | |
| Linear | 0.881 | 1.437 | 3.067 | <0.0001 | 120.292 | 0.321 | 0.321 | Questionable |  |
| * BMDS recommended best fitting model  † User selected best fitting model | | | | | | | | | |

### Dataset Name: MucilAir Oleoyl sarcosine, TEER (Ω.cm)

| Dose (µg/cm²) | N | Mean | Std. Dev. |
| --- | --- | --- | --- |
| 0 | 3 | 614.9 | 103.14 |
| 5.2 | 3 | 579.865 | 127.689 |
| 10.3 | 3 | 510.758 | 119.971 |
| 20.1 | 3 | 119.397 | 45.823 |
| 41.1 | 3 | 48.455 | 8.213 |
| 77.8 | 3 | 46.063 | 6.716 |
| Test 1 Dose response: < 0.0001  Test 2 Homogeneity of Variance: < 0.0001  Test 3 Variance Model Selection: 0.435 | | | |

| **BMR Type** | Std. Dev. |
| --- | --- |
| **BMRF** | 1 |
| **Distribution Type** | Normal + Non-constant |
| **Adverse Direction** | Automatic |
| **Maximum Polynomial Degree** | 3 |
| **Confidence Level (one sided)** | 0.95 |

Maximum Likelihood Approach Model Results

| **Model** | **BMDL (µg/cm²)** | **BMD (µg/cm²)** | **BMDU (µg/cm²)** | ***P*-Value** | **AIC** | **Scaled Residual at Control** | **Scaled Residual near BMD** | **Recommendation and Notes** |  |
| --- | --- | --- | --- | --- | --- | --- | --- | --- | --- |
| **Restricted Models** | | | | | | | | | |
| Exponential 3 | 5.957 | 9.485 | 12.778 | <0.0001 | 215.123 | 0.179 | 0.159 | Questionable |  |
| Exponential 5 | 7.774 | 11.254 | 18.551 | 0.908 | 192.417 | 0.293 | 0.033 | Viable |  |
| Hill*† | 9.179 | 11.115 | 16.369 | 0.963 | 192.298 | 0.334 | 0.034 | Recommended - Lowest AIC |  |
| Polynomial 2 | 27.74 | 45.972 | - | <0.0001 | 237.226 | 1.176 | 0.053 | Questionable |  |
| Polynomial 3 | 44.889 | 45.409 | 47.911 | <0.0001 | 237.106 | 1.147 | 0.036 | Questionable |  |
| Power | 38.423 | 53.291 | 54.394 | <0.0001 | 232.215 | 1.341 | -2.802 | Questionable |  |
| **Unrestricted Models** | | | | | | | | | |
| Linear | - | 35863.664 | - | <0.0001 | 251.177 | 1.619 | 1.739 | Unusable |  |
| * BMDS recommended best fitting model  † User selected best fitting model | | | | | | | | | |

### Dataset Name: MucilAir Oleoyl sarcosine, AAA (rel. vehicle)

| Dose (µg/cm²) | N | Mean | Std. Dev. |
| --- | --- | --- | --- |
| 0 | 3 | 1 | 0.076 |
| 5.2 | 3 | 1.002 | 0.158 |
| 10.3 | 3 | 1.069 | 0.069 |
| 20.1 | 3 | 0.918 | 0.082 |
| 41.1 | 3 | 0.648 | 0.359 |
| 77.8 | 3 | 0.127 | 0.122 |
| Test 1 Dose response: < 0.0001  Test 2 Homogeneity of Variance: 0.0168  Test 3 Variance Model Selection: 0.0168 | | | |

| **BMR Type** | Std. Dev. |
| --- | --- |
| **BMRF** | 1 |
| **Distribution Type** | Normal + Constant |
| **Adverse Direction** | Automatic |
| **Maximum Polynomial Degree** | 3 |
| **Confidence Level (one sided)** | 0.95 |

Maximum Likelihood Approach Model Results

| **Model** | **BMDL (µg/cm²)** | **BMD (µg/cm²)** | **BMDU (µg/cm²)** | ***P*-Value** | **AIC** | **Scaled Residual at Control** | **Scaled Residual near BMD** | **Recommendation and Notes** | |
| --- | --- | --- | --- | --- | --- | --- | --- | --- | --- |
| **Restricted Models** | | | | | | | | |  |
| Exponential 3* | 16.038 | 25.938 | 38.069 | 0.858 | -9.982 | -0.281 | -0.269 | Questionable | |
| Exponential 5 | 16.038 | 26.126 | 40.496 | 0.683 | -7.983 | -0.273 | -0.288 | Questionable | |
| Hill | 12.965 | 25.99 | 39.957 | 0.688 | -7.998 | -0.27 | -0.286 | Questionable | |
| Polynomial 2 | 11.186 | 21.712 | 37.469 | 0.665 | -9.172 | -0.525 | 0.1 | Questionable | |
| Polynomial 3 | 11.016 | 18.605 | 18.99 | 0.630 | -9.013 | -0.702 | 0.249 | Questionable | |
| Power | 11.613 | 22.577 | 38.617 | 0.742 | -9.498 | -0.451 | 0.054 | Questionable | |
| **Unrestricted Models** | | | | | | | | |  |
| Linear | 9.851 | 13.231 | 19.621 | 0.479 | -9.254 | -1.118 | 0.985 | Questionable | |
| * User selected best fitting model | | | | | | | | |  |

### Dataset Name: MucilAir Oleoyl sarcosine, CBF (Hz)

| Dose (µg/cm²) | N | Mean | Std. Dev. |
| --- | --- | --- | --- |
| 0 | 3 | 10.546 | 1.943 |
| 5.2 | 3 | 9.959 | 1.514 |
| 10.3 | 3 | 10.363 | 2.067 |
| 20.1 | 3 | 10.214 | 2.625 |
| 41.1 | 3 | 8.948 | 1.265 |
| 77.8 | 3 | 8.964 | 1.636 |
| Test 1 Dose response: 0.899  Test 2 Homogeneity of Variance: 0.850  Test 3 Variance Model Selection: 0.850 | | | |

| **BMR Type** | Std. Dev. |
| --- | --- |
| **BMRF** | 1 |
| **Distribution Type** | Normal + Constant |
| **Adverse Direction** | Automatic |
| **Maximum Polynomial Degree** | 3 |
| **Confidence Level (one sided)** | 0.95 |

Maximum Likelihood Approach Model Results

| **Model** | **BMDL (µg/cm²)** | **BMD (µg/cm²)** | **BMDU (µg/cm²)** | ***P*-Value** | **AIC** | **Scaled Residual at Control** | **Scaled Residual near BMD** | **Recommendation and Notes** |  |
| --- | --- | --- | --- | --- | --- | --- | --- | --- | --- |
| **Restricted Models** | | | | | | | | | |
| Exponential 3*† | 31.732 | 74.053 | 71,613.172 | 0.982 | 73.474 | 0.168 | 0.243 | Recommended - Lowest AIC |  |
| Exponential 5 | - | - | - | 0.973 | 74.988 | 0.286 | - | Unusable |  |
| Hill | - | - | - | 0.893 | 76.988 | 0.286 | - | Unusable |  |
| Polynomial 2 | 35.438 | 75.439 | - | 0.946 | 73.502 | 0.192 | 0.242 | Viable |  |
| Polynomial 3 | 35.438 | 75.531 | - | 0.946 | 73.502 | 0.193 | 0.24 | Viable |  |
| Power | 35.438 | 75.315 | - | 0.946 | 73.502 | 0.191 | 0.244 | Viable |  |
| **Unrestricted Models** | | | | | | | | | |
| Linear | 35.438 | 75.315 | - | 0.946 | 73.502 | 0.191 | 0.244 | Viable |  |
| * BMDS recommended best fitting model  † User selected best fitting model | | | | | | | | | |

## MucilAir: Oleoyl sarcosine, 7 d

### Dataset Name: 7d MucilAir Oleoyl sarcosine, IL-6 (fold-change rel. vehicle)

| Dose (µg/cm²) | N | Mean | Std. Dev. |
| --- | --- | --- | --- |
| 0 | 3 | 1 | 0.857 |
| 5.2 | 3 | 0.66 | 0.321 |
| 10.3 | 3 | 0.704 | 0.423 |
| 20.1 | 3 | 0.315 | 0.117 |
| Test 1 Dose response: 0.0423  Test 2 Homogeneity of Variance: 0.0233  Test 3 Variance Model Selection: 0.943 | | | |

| **BMR Type** | Std. Dev. |
| --- | --- |
| **BMRF** | 1 |
| **Distribution Type** | Normal + Non-constant |
| **Adverse Direction** | Automatic |
| **Maximum Polynomial Degree** | 3 |
| **Confidence Level (one sided)** | 0.95 |

Maximum Likelihood Approach Model Results

| **Model** | **BMDL (µg/cm²)** | **BMD (µg/cm²)** | **BMDU (µg/cm²)** | ***P*-Value** | **AIC** | **Scaled Residual at Control** | **Scaled Residual near BMD** | **Recommendation and Notes** |  |
| --- | --- | --- | --- | --- | --- | --- | --- | --- | --- |
| **Restricted Models** | | | | | | | | | |
| Exponential 3 | 6.909 | 19.633 | - | 0.148 | 15.62 | 0.135 | -0.073 | Viable |  |
| Exponential 5 | 6.909 | 19.633 | - | - | 17.62 | 0.135 | -0.073 | Questionable |  |
| Hill | 6.313 | 19.198 | - | - | 17.382 | 0.072 | -0.042 | Questionable |  |
| Polynomial 2 | 10.594 | 19.582 | 60.769 | 0.182 | 15.309 | 0.148 | -0.036 | Viable |  |
| Polynomial 3 | 10.618 | 19.73 | 60.249 | 0.185 | 15.284 | 0.181 | -0.034 | Viable |  |
| Power | 10.584 | 19.959 | 61.009 | 0.408 | 13.32 | 0.134 | -0.066 | Viable |  |
| **Unrestricted Models** | | | | | | | | | |
| Linear*† | 10.588 | 19.422 | 50.22 | 0.409 | 13.316 | 0.112 | -0.038 | Recommended - Lowest AIC |  |
| * BMDS recommended best fitting model  † User selected best fitting model | | | | | | | | | |

### Dataset Name: 7d MucilAir Oleoyl sarcosine, IL-8 (rel. vehicle)

| Dose (µg/cm²) | N | Mean | Std. Dev. |
| --- | --- | --- | --- |
| 0 | 3 | 1 | 0.113 |
| 5.2 | 3 | 0.761 | 0.132 |
| 10.3 | 3 | 0.646 | 0.172 |
| 20.1 | 3 | 0.622 | 0.218 |
| Test 1 Dose response: 0.0807  Test 2 Homogeneity of Variance: 0.679  Test 3 Variance Model Selection: 0.679 | | | |

| **BMR Type** | Std. Dev. |
| --- | --- |
| **BMRF** | 1 |
| **Distribution Type** | Normal + Constant |
| **Adverse Direction** | Automatic |
| **Maximum Polynomial Degree** | 3 |
| **Confidence Level (one sided)** | 0.95 |

Maximum Likelihood Approach Model Results

| **Model** | **BMDL (µg/cm²)** | **BMD (µg/cm²)** | **BMDU (µg/cm²)** | ***P*-Value** | **AIC** | **Scaled Residual at Control** | **Scaled Residual near BMD** | **Recommendation and Notes** |  |
| --- | --- | --- | --- | --- | --- | --- | --- | --- | --- |
| **Restricted Models** | | | | | | | | | |
| Exponential 3*† | 3.604 | 6.598 | 20.328 | 0.284 | -5.714 | 0.733 | -0.664 | Recommended - Lowest AIC |  |
| Exponential 5 | 0.08 | 2.97 | 9.015 | - | -4.232 | 6.26e-9 | 1.23e-8 | Questionable |  |
| Hill | 0 | 3.545 | 9.022 | - | -4.232 | -5.82e-8 | 2.96e-8 | Unusable |  |
| Polynomial 2 | 5.294 | 8.768 | 25.084 | 0.194 | -4.949 | 0.985 | -0.98 | Viable |  |
| Polynomial 3 | 5.294 | 8.784 | 25.083 | 0.194 | -4.949 | 0.987 | -0.981 | Viable |  |
| Power | 5.294 | 8.785 | 25.084 | 0.194 | -4.949 | 0.987 | -0.981 | Viable |  |
| **Unrestricted Models** | | | | | | | | | |
| Linear | 5.294 | 8.785 | 25.086 | 0.194 | -4.949 | 0.987 | -0.981 | Viable |  |
| * BMDS recommended best fitting model  † User selected best fitting model | | | | | | | | | |

### Dataset Name: 7d MucilAir Oleoyl sarcosine, TEER (Ω·cm2)

| Dose (µg/cm²) | N | Mean | Std. Dev. |
| --- | --- | --- | --- |
| 0 | 3 | 537.955 | 191.271 |
| 5.2 | 3 | 547.47 | 133.217 |
| 10.3 | 3 | 553.41 | 129.53 |
| 20.1 | 3 | 645.755 | 123.211 |
| 41.1 | 3 | 346.72 | 231.768 |
| 77.8 | 3 | 69.85 | 29.569 |
| Test 1 Dose response: 0.000583  Test 2 Homogeneity of Variance: 0.101  Test 3 Variance Model Selection: 0.101 | | | |

| **BMR Type** | Std. Dev. |
| --- | --- |
| **BMRF** | 1 |
| **Distribution Type** | Normal + Constant |
| **Adverse Direction** | Automatic |
| **Maximum Polynomial Degree** | 3 |
| **Confidence Level (one sided)** | 0.95 |

Maximum Likelihood Approach Model Results

| **Model** | **BMDL (µg/cm²)** | **BMD (µg/cm²)** | **BMDU (µg/cm²)** | ***P*-Value** | **AIC** | **Scaled Residual at Control** | **Scaled Residual near BMD** | **Recommendation and Notes** |  |
| --- | --- | --- | --- | --- | --- | --- | --- | --- | --- |
| **Restricted Models** | | | | | | | | | |
| Exponential 3*† | 23.747 | 39.683 | 51.484 | 0.816 | 233.17 | -0.432 | -4.06e-6 | Recommended - Lowest AIC |  |
| Exponential 5 | 26.202 | 39.573 | 47.631 | 0.708 | 234.324 | -0.442 | -4.61e-6 | Viable |  |
| Hill | 26.466 | 39.228 | 42.433 | 0.708 | 234.324 | -0.442 | -8.45e-6 | Viable |  |
| Polynomial 2 | 18.643 | 40.41 | 51.934 | 0.479 | 234.428 | -0.361 | -0.972 | Viable |  |
| Polynomial 3 | 17.538 | 40.688 | 41.531 | 0.134 | 238.961 | -0.441 | -1.046 | Viable |  |
| Power | 32.638 | 37.547 | 60.112 | 0.330 | 236.364 | -0.442 | -0.809 | Viable |  |
| **Unrestricted Models** | | | | | | | | | |
| Linear | 15.556 | 22.211 | 38.111 | 0.227 | 236.584 | -0.97 | 1.873 | Viable |  |
| * BMDS recommended best fitting model  † User selected best fitting model | | | | | | | | | |

### Dataset Name: 7d MucilAir Oleoyl sarcosine, Viability (rel. vehicle)

| Dose (µg/cm²) | N | Mean | Std. Dev. |
| --- | --- | --- | --- |
| 0 | 3 | 1 | 0.133 |
| 5.2 | 3 | 0.909 | 0.223 |
| 10.3 | 3 | 0.981 | 0.211 |
| 20.1 | 3 | 0.792 | 0.077 |
| 41.1 | 3 | 0.166 | 0.075 |
| 77.8 | 3 | 0.132 | 0.103 |
| Test 1 Dose response: < 0.0001  Test 2 Homogeneity of Variance: 0.253  Test 3 Variance Model Selection: 0.253 | | | |

| **BMR Type** | Std. Dev. |
| --- | --- |
| **BMRF** | 1 |
| **Distribution Type** | Normal + Constant |
| **Adverse Direction** | Automatic |
| **Maximum Polynomial Degree** | 3 |
| **Confidence Level (one sided)** | 0.95 |

Maximum Likelihood Approach Model Results

| **Model** | **BMDL (µg/cm²)** | **BMD (µg/cm²)** | **BMDU (µg/cm²)** | ***P*-Value** | **AIC** | **Scaled Residual at Control** | **Scaled Residual near BMD** | **Recommendation and Notes** |
| --- | --- | --- | --- | --- | --- | --- | --- | --- |
| **Restricted Models** | | | | | | | | |
| Exponential 3 | 12.02 | 18.569 | 29.486 | 0.391 | -12.521 | 0.378 | -0.087 | Viable |
| Exponential 5 | 12.998 | 18.344 | 26.576 | 0.601 | -13.613 | 0.446 | -0.059 | Viable |
| Hill*† | 14.773 | 18.942 | 24.716 | 0.629 | -13.707 | 0.496 | -0.011 | Recommended - Lowest AIC |
| Polynomial 2 | 11.008 | 14.932 | 23.502 | 0.00319 | -2.756 | 0.086 | 1.108 | Questionable |
| Polynomial 3 | 11.008 | 14.931 | 23.501 | 0.00319 | -2.756 | 0.086 | 1.108 | Questionable |
| Power | 11.008 | 14.933 | 25.179 | 0.00319 | -2.756 | 0.087 | 1.108 | Questionable |
| **Unrestricted Models** | | | | | | | | |
| Linear | 11.008 | 14.933 | 22.664 | 0.00319 | -2.756 | 0.087 | 1.108 | Questionable |
| * BMDS recommended best fitting model  † User selected best fitting model | | | | | | | | |

### Dataset Name: 7d MucilAir Oleoyl sarcosine, AAA (%AAA)

| Dose (µg/cm²) | N | Mean | Std. Dev. |
| --- | --- | --- | --- |
| 0 | 3 | 99.273 | 1.111 |
| 5.2 | 3 | 99.672 | 0.276 |
| 10.3 | 3 | 99.846 | 0.165 |
| 20.1 | 3 | 80.578 | 29.665 |
| 41.1 | 3 | 5.362 | 5.374 |
| 77.8 | 3 | 1.966 | 1.038 |
| Test 1 Dose response: 0  Test 2 Homogeneity of Variance: < 0.0001  Test 3 Variance Model Selection: < 0.0001 | | | |

| **BMR Type** | Std. Dev. |
| --- | --- |
| **BMRF** | 1 |
| **Distribution Type** | Normal + Constant |
| **Adverse Direction** | Automatic |
| **Maximum Polynomial Degree** | 3 |
| **Confidence Level (one sided)** | 0.95 |

Maximum Likelihood Approach Model Results

| **Model** | **BMDL (µg/cm²)** | **BMD (µg/cm²)** | **BMDU (µg/cm²)** | ***P*-Value** | **AIC** | **Scaled Residual at Control** | **Scaled Residual near BMD** | **Recommendation and Notes** |  |
| --- | --- | --- | --- | --- | --- | --- | --- | --- | --- |
| **Restricted Models** | | | | | | | | | |
| Exponential 3* | 12.908 | 16.622 | 21.916 | 0.995 | 142.41 | -0.153 | -0.05 | Questionable |  |
| Exponential 5 | 12.994 | 16.756 | 21.827 | 0.960 | 144.281 | -0.143 | -0.041 | Questionable |  |
| Hill | 14.914 | 17.954 | 21.251 | 0.995 | 144.21 | -0.071 | -0.003 | Questionable |  |
| Polynomial 2 | 9.955 | 13.391 | 20.379 | <0.0001 | 164.873 | -0.31 | 1.072 | Questionable |  |
| Polynomial 3 | 9.955 | 13.336 | 20.384 | <0.0001 | 164.874 | -0.324 | 1.063 | Questionable |  |
| Power | 9.955 | 13.382 | 22.148 | <0.0001 | 164.873 | -0.312 | 1.07 | Questionable |  |
| **Unrestricted Models** | | | | | | | | | |
| Linear | 9.955 | 13.382 | 19.884 | <0.0001 | 164.873 | -0.312 | 1.07 | Questionable |  |
| * User selected best fitting model | | | | | | | | | |

### Dataset Name: 7d MucilAir Oleoyl sarcosine, CBF (Hz)

| Dose (µg/cm²) | N | Mean | Std. Dev. |
| --- | --- | --- | --- |
| 0 | 3 | 8.06 | 1.946 |
| 5.2 | 3 | 7.662 | 1.259 |
| 10.3 | 3 | 7.948 | 1.068 |
| 20.1 | 3 | 6.029 | 1.879 |
| Test 1 Dose response: 0.477  Test 2 Homogeneity of Variance: 0.684  Test 3 Variance Model Selection: 0.684 | | | |

| **BMR Type** | Std. Dev. |
| --- | --- |
| **BMRF** | 1 |
| **Distribution Type** | Normal + Constant |
| **Adverse Direction** | Automatic |
| **Maximum Polynomial Degree** | 3 |
| **Confidence Level (one sided)** | 0.95 |

Maximum Likelihood Approach Model Results

| **Model** | **BMDL (µg/cm²)** | **BMD (µg/cm²)** | **BMDU (µg/cm²)** | ***P*-Value** | **AIC** | **Scaled Residual at Control** | **Scaled Residual near BMD** | **Recommendation and Notes** |  |
| --- | --- | --- | --- | --- | --- | --- | --- | --- | --- |
| **Restricted Models** | | | | | | | | | |
| Exponential 3*† | 7.138 | 19.658 | 67.305 | 0.985 | 46.386 | 0.226 | -6.36e-7 | Recommended - Lowest AIC |  |
| Exponential 5 | 6.025 | 19.658 | 67.319 | 0.698 | 48.386 | 0.226 | -5.20e-7 | Viable |  |
| Hill | 5.712 | 19.69 | - | - | 50.386 | 0.226 | -4.69e-7 | Questionable |  |
| Polynomial 2 | 7.551 | 16.435 | 83.793 | 0.811 | 46.654 | -0.025 | -0.107 | Viable |  |
| Polynomial 3 | 7.697 | 17.67 | 69.359 | 0.884 | 46.484 | 0.108 | -0.034 | Viable |  |
| Power | 7.786 | 19.705 | 62.913 | 0.698 | 48.386 | 0.226 | -4.80e-7 | Viable |  |
| **Unrestricted Models** | | | | | | | | | |
| Linear | 7.11 | 13.879 | 261.903 | 0.599 | 47.261 | -0.296 | 0.846 | Viable |  |
| * BMDS recommended best fitting model  † User selected best fitting model | | | | | | | | | |

## BEAS-2B: Triton X-100, 24 h

### Dataset Name: BEAS-2B Triton X-100, IL-6 (rel. vehicle)

| **Dose (µg/cm²)** | **N** | **Mean** | **Std. Dev.** |
| --- | --- | --- | --- |
| 0 | 3 | 1 | 1.44 |
| 2.2 | 3 | 0.44 | 0.241 |
| 5.5 | 3 | 1.118 | 0.713 |
| 7.9 | 3 | 7.754 | 6.213 |
| Test 1 Dose response: < 0.0001  Test 2 Homogeneity of Variance: < 0.0001  Test 3 Variance Model Selection: < 0.0001 | | | |

| **BMR Type** | Std. Dev. |
| --- | --- |
| **BMRF** | 1 |
| **Distribution Type** | Normal + Constant |
| **Adverse Direction** | Automatic |
| **Maximum Polynomial Degree** | 3 |
| **Confidence Level (one sided)** | 0.95 |

Maximum Likelihood Approach Model Results

| **Model** | **BMDL (µg/cm²)** | **BMD (µg/cm²)** | **BMDU (µg/cm²)** | ***P*-Value** | **AIC** | **Scaled Residual at Control** | **Scaled Residual near BMD** | **Recommendation and Notes** |
| --- | --- | --- | --- | --- | --- | --- | --- | --- |
| **Restricted Models** | | | | | | | | |
| Exponential 3 | 5.145 | 7.204 | 7.859 | 0.966 | 65.256 | 0.182 | -1.06e-4 | Questionable |
| Exponential 5 | 5.177 | 6.979 | 7.767 | - | 67.255 | 0.184 | -1.84e-5 | Questionable |
| Hill | 4.793 | 6.474 | 7.819 | - | 67.255 | 0.184 | 2.94e-7 | Questionable |
| Polynomial 2 | 3.263 | 5.17 | 8.242 | 0.273 | 65.781 | 0.644 | -1.246 | Questionable |
| Polynomial 3* | 3.882 | 5.766 | 7.584 | 0.534 | 64.442 | 0.524 | -0.905 | Questionable |
| Power | 4.77 | 6.979 | 7.325 | 0.794 | 65.255 | 0.184 | -1.84e-5 | Questionable |
| **Unrestricted Models** | | | | | | | | |
| Linear | 2.414 | 4.212 | 16.019 | 0.0804 | 68.229 | 0.759 | -1.44 | Questionable |
| - * User selected best fitting model; rationale - lowest AIC, reasonable p-value + residuals | | | | | | | | |

### Dataset Name: BEAS-2B Triton X-100, IL-8 (rel. vehicle)

| **Dose (µg/cm²)** | **N** | **Mean** | **Std. Dev.** |
| --- | --- | --- | --- |
| 0 | 3 | 1 | 0.769 |
| 2.2 | 3 | 0.864 | 0.25 |
| 5.5 | 3 | 1.052 | 0.387 |
| 7.9 | 3 | 4.818 | 2.118 |
| Test 1 Dose response: < 0.0001  Test 2 Homogeneity of Variance: 0.00236  Test 3 Variance Model Selection: 0.295 | | | |

| **BMR Type** | Std. Dev. |
| --- | --- |
| **BMRF** | 1 |
| **Distribution Type** | Normal + Non-constant |
| **Adverse Direction** | Automatic |
| **Maximum Polynomial Degree** | 3 |
| **Confidence Level (one sided)** | 0.95 |

Maximum Likelihood Approach Model Results

| **Model** | **BMDL (µg/cm²)** | **BMD (µg/cm²)** | **BMDU (µg/cm²)** | ***P*-Value** | **AIC** | **Scaled Residual at Control** | **Scaled Residual near BMD** | **Recommendation and Notes** |
| --- | --- | --- | --- | --- | --- | --- | --- | --- |
| **Restricted Models** | | | | | | | | |
| Exponential 3 | 5.09 | 7.117 | 7.479 | 0.205 | 32.146 | 0.127 | -0.001 | Viable |
| Exponential 5 | 6.614 | 6.896 | 7.146 | - | 34.146 | 0.13 | -0.002 | Questionable |
| Hill | 5.172 | 6.869 | 7.301 | - | 34.146 | 0.13 | -0.002 | Questionable |
| Polynomial 2 | 2.335 | 3.584 | 14.733 | 0.00322 | 40.015 | 0.518 | -0.507 | Questionable |
| Polynomial 3 | 3.186 | 4.315 | 10.846 | 0.0152 | 36.914 | 0.542 | -1.387 | Questionable |
| Power*† | 5.158 | 6.896 | 7.351 | 0.205 | 32.146 | 0.13 | -0.002 | Recommended - Lowest AIC |
| **Unrestricted Models** | | | | | | | | |
| Linear | 1.135 | 2.669 | 40.375 | 0.000260 | 45.051 | 0.28 | -0.91 | Questionable |
| - * BMDS recommended best fitting model - † User selected best fitting model | | | | | | | | |

### Dataset Name: BEAS-2B Triton X-100, Viability (rel. vehicle)

| **Dose (µg/cm²)** | **N** | **Mean** | **Std. Dev.** |
| --- | --- | --- | --- |
| 0 | 3 | 1 | 0.156 |
| 2.2 | 3 | 1.037 | 0.09 |
| 5.5 | 3 | 1.009 | 0.093 |
| 7.9 | 3 | 0.777 | 0.092 |
| 13.9 | 3 | 0.058 | 0.084 |
| 16.4 | 3 | -0.001 | 0.004 |
| Test 1 Dose response: < 0.0001  Test 2 Homogeneity of Variance: 0.00269  Test 3 Variance Model Selection: 0.412 | | | |

| **BMR Type** | Std. Dev. |
| --- | --- |
| **BMRF** | 1 |
| **Distribution Type** | Normal + Non-constant |
| **Adverse Direction** | Automatic |
| **Maximum Polynomial Degree** | 3 |
| **Confidence Level (one sided)** | 0.95 |

Maximum Likelihood Approach Model Results

| **Model** | **BMDL (µg/cm²)** | **BMD (µg/cm²)** | **BMDU (µg/cm²)** | ***P*-Value** | **AIC** | **Scaled Residual at Control** | **Scaled Residual near BMD** | **Recommendation and Notes** |
| --- | --- | --- | --- | --- | --- | --- | --- | --- |
| **Restricted Models** | | | | | | | | |
| Exponential 3 | 5.633 | 7.183 | 8.708 | 0.0736 | -37.468 | -0.153 | -0.875 | Questionable |
| Exponential 5 | 5.633 | 7.183 | 8.708 | 0.0310 | -35.468 | -0.153 | -0.875 | Questionable |
| Hill*† | 6.24 | 6.526 | 6.861 | 0.634 | -41.506 | -1.3 | -0.574 | Recommended - Lowest AIC |
| Polynomial 2 | 2.113 | 2.804 | 2.862 | 0.000390 | -26.163 | -2.067 | -0.373 | Questionable |
| Polynomial 3 | 2.83 | 4.155 | 4.241 | 0.000194 | -25.321 | -0.649 | 1.884 | Questionable |
| Power | 4.209 | 4.924 | 5.026 | 0.000931 | -27.998 | -0.795 | 1.434 | Questionable |
| **Unrestricted Models** | | | | | | | | |
| Linear | 1.95 | 2.912 | 2.972 | 0.000524 | -26.523 | -1.499 | 0.119 | Questionable |
| - * BMDS recommended best fitting model - † User selected best fitting model | | | | | | | | |

### Dataset Name: BEAS-2B Triton X-100, LDH (rel. vehicle)

| **Dose (µg/cm²)** | **N** | **Mean** | **Std. Dev.** |
| --- | --- | --- | --- |
| 0 | 3 | 1 | 0.311 |
| 2.2 | 3 | 0.748 | 0.17 |
| 5.5 | 3 | 0.897 | 0.185 |
| 7.9 | 3 | 1.025 | 0.082 |
| 13.9 | 3 | 2.601 | 0.847 |
| 16.4 | 3 | 3.205 | 0.281 |
| Test 1 Dose response: < 0.0001  Test 2 Homogeneity of Variance: 0.00243  Test 3 Variance Model Selection: 0.0827 | | | |

| **BMR Type** | Std. Dev. |
| --- | --- |
| **BMRF** | 1 |
| **Distribution Type** | Normal + Non-constant |
| **Adverse Direction** | Automatic |
| **Maximum Polynomial Degree** | 3 |
| **Confidence Level (one sided)** | 0.95 |

Maximum Likelihood Approach Model Results

| **Model** | **BMDL (µg/cm²)** | **BMD (µg/cm²)** | **BMDU (µg/cm²)** | ***P*-Value** | **AIC** | **Scaled Residual at Control** | **Scaled Residual near BMD** | **Recommendation and Notes** |
| --- | --- | --- | --- | --- | --- | --- | --- | --- |
| **Restricted Models** | | | | | | | | |
| Exponential 3 | 4.835 | 7.101 | 9.608 | 0.0267 | 19.854 | 1.313 | -0.579 | Questionable |
| Exponential 5 | 6.613 | 8.799 | 12.312 | 0.141 | 16.568 | 1.009 | 0.299 | Viable |
| Hill | 6.656 | 8.69 | 12.088 | 0.142 | 16.561 | 1.003 | 0.29 | Viable |
| Polynomial 2 | 4.246 | 5.311 | 6.713 | 0.0200 | 20.32 | 1.677 | -0.765 | Questionable |
| Polynomial 3*† | 5.494 | 7.118 | 8.319 | 0.122 | 15.922 | 1.356 | -0.636 | Recommended - Lowest AIC |
| Power | 5.552 | 7.574 | 9.845 | 0.0686 | 17.76 | 1.262 | -0.418 | Questionable |
| **Unrestricted Models** | | | | | | | | |
| Linear | 1.862 | 2.923 | 5.079 | < 0.0001 | 34.363 | 1.799 | -0.589 | Questionable |
| - * BMDS recommended best fitting model - † User selected best fitting model | | | | | | | | |

## MucilAir: Triton X-100, 24 h

### Dataset Name: MucilAir Triton X-100, IL-6 (rel. vehicle)

| **Dose (µg/cm²)** | **N** | **Mean** | **Std. Dev.** |
| --- | --- | --- | --- |
| 0 | 3 | 1 | 0.341 |
| 4.2 | 3 | 10.251 | 3.949 |
| 9.1 | 3 | 68.601 | 35.017 |
| 18.6 | 3 | 106.988 | 20.748 |
| Test 1 Dose response: < 0.0001  Test 2 Homogeneity of Variance: < 0.0001  Test 3 Variance Model Selection: 0.331 | | | |

| **BMR Type** | Std. Dev. |
| --- | --- |
| **BMRF** | 1 |
| **Distribution Type** | Normal + Non-constant |
| **Adverse Direction** | Automatic |
| **Maximum Polynomial Degree** | 3 |
| **Confidence Level (one sided)** | 0.95 |

Maximum Likelihood Approach Model Results

| **Model** | **BMDL (µg/cm²)** | **BMD (µg/cm²)** | **BMDU (µg/cm²)** | ***P*-Value** | **AIC** | **Scaled Residual at Control** | **Scaled Residual near BMD** | **Recommendation and Notes** |  |
| --- | --- | --- | --- | --- | --- | --- | --- | --- | --- |
| **Restricted Models** | | | | | | | | | |
| Exponential 3 | 0.508 | 1.053 | 3.224 | < 0.0001 | 113.579 | -1.269 | -1.269 | Questionable |  |
| Exponential 5 | 0.907 | 1.461 | 3.549 | - | 84.713 | 0.044 | 0.044 | Questionable |  |
| Hill*† | 1.153 | 1.791 | 3.07 | 0.926 | 82.721 | 0.039 | 0.039 | Recommended - Lowest AIC |  |
| Polynomial 2 | 0.167 | 0.695 | 1.089 | 0.00354 | 92.001 | 0.019 | 0.019 | Questionable |  |
| Polynomial 3 | 0.167 | 0.695 | 1.089 | 0.00354 | 92.001 | 0.019 | 0.019 | Questionable |  |
| Power | 0.044 | 0.485 | 1.139 | 0.000952 | 93.632 | 0.082 | 0.082 | Questionable |  |
| **Unrestricted Models** | | | | | | | | | |
| Linear | 0.169 | 0.293 | 0.769 | < 0.0001 | 101.839 | 0.017 | 0.017 | Questionable |  |
| - * BMDS recommended best fitting model - † User selected best fitting model | | | | | | | | | |

### Dataset Name: MucilAir Triton X-100, IL-8 (rel. vehicle)

| **Dose (µg/cm²)** | **N** | **Mean** | | **Std. Dev.** | |
| --- | --- | --- | --- | --- | --- |
| 0 | 3 | 1 | | 0.208 | |
| 4.2 | 3 | 2.082 | | 0.314 | |
| 9.1 | 3 | 2.422 | | 0.206 | |
| 18.6 | 3 | 2.353 | | 0.318 | |
| Test 1 Dose response: 0.000245  Test 2 Homogeneity of Variance: 0.791  Test 3 Variance Model Selection: 0.791 | | | | | |
| **BMR Type** | | | Std. Dev. | |  |
| **BMRF** | | | 1 | |  |
| **Distribution Type** | | | Normal + Constant | |  |
| **Adverse Direction** | | | Automatic | |  |
| **Maximum Polynomial Degree** | | | 3 | |  |
| **Confidence Level (one sided)** | | | 0.95 | |  |

Maximum Likelihood Approach Model Results

| **Model** | **BMDL (µg/cm²)** | **BMD (µg/cm²)** | **BMDU (µg/cm²)** | ***P*-Value** | **AIC** | **Scaled Residual at Control** | **Scaled Residual near BMD** | **Recommendation and Notes** |  |
| --- | --- | --- | --- | --- | --- | --- | --- | --- | --- |
| **Restricted Models** | | | | | | | | | |
| Exponential 3 | 6.565 | 9.704 | 22.709 | 0.000127 | 21.454 | -2.126 | 1.609 | Questionable |  |
| Exponential 5 | 0.232 | 2.255 | 3.89 | - | 7.657 | -8.41e-9 | -1.45e-8 | Questionable |  |
| Hill*† | 2.941 | 3.568 | 3.803 | 0.699 | 5.657 | -3.65e-8 | -4.13e-9 | Recommended - Lowest AIC |  |
| Polynomial 2 | 4.412 | 7.027 | 16.779 | 0.000250 | 20.097 | -1.87 | 1.544 | Questionable |  |
| Polynomial 3 | 4.412 | 7.026 | 16.779 | 0.000250 | 20.097 | -1.87 | 1.544 | Questionable |  |
| Power | 4.412 | 7.03 | 16.779 | 0.000250 | 20.097 | -1.871 | 1.544 | Questionable |  |
| **Unrestricted Models** | | | | | | | | | |
| Linear | 4.412 | 7.03 | 16.779 | 0.000250 | 20.097 | -1.871 | 1.544 | Questionable |  |
| - * BMDS recommended best fitting model - † User selected best fitting model | | | | | | | | | |

### Dataset Name: MucilAir Triton X-100, Viability

- Unsuitable for BMDS due to biphasic nature, no model selected
- NOAEC = 4.20 µg/cm^2^; LOAEC = 9.1 µg/cm^2^

### Dataset Name: MucilAir Triton X-100, LDH (rel. vehicle)

| **Dose (µg/cm²)** | **N** | **Mean** | **Std. Dev.** |
| --- | --- | --- | --- |
| 0 | 3 | 1 | 0.373 |
| 4.2 | 3 | 0.817 | 0.472 |
| 9.1 | 3 | 1.213 | 0.429 |
| 18.6 | 3 | 2.347 | 1.325 |
| 37.8 | 3 | 14.647 | 6.842 |
| 77.9 | 3 | 60.3 | 17.737 |
| Test 1 Dose response: 0  Test 2 Homogeneity of Variance: < 0.0001  Test 3 Variance Model Selection: 0.849 | | | |

| **BMR Type** | Std. Dev. |
| --- | --- |
| **BMRF** | 1 |
| **Distribution Type** | Normal + Non-constant |
| **Adverse Direction** | Automatic |
| **Maximum Polynomial Degree** | 3 |
| **Confidence Level (one sided)** | 0.95 |

Maximum Likelihood Approach Model Results

| **Model** | **BMDL (µg/cm²)** | **BMD (µg/cm²)** | **BMDU (µg/cm²)** | ***P*-Value** | **AIC** | **Scaled Residual at Control** | **Scaled Residual near BMD** | **Recommendation and Notes** |  |
| --- | --- | --- | --- | --- | --- | --- | --- | --- | --- |
| **Restricted Models** | | | | | | | | | |
| Exponential 3 | 3.934 | 5.759 | 9.297 | 0.00331 | 83.929 | 0.839 | -0.93 | Questionable |  |
| Exponential 5*† | 7.814 | 11.734 | 16.285 | 0.853 | 72.455 | 0.368 | 0.512 | Recommended - Lowest AIC |  |
| Hill | 8.252 | 11.82 | 16.352 | 0.847 | 72.47 | 0.358 | 0.53 | Viable |  |
| Polynomial 2 | 4.863 | 6.43 | 8.353 | 0.163 | 74.659 | 1.09 | -0.487 | Viable |  |
| Polynomial 3 | 5.315 | 8.082 | 12.719 | 0.244 | 74.308 | 0.865 | -0.204 | Viable |  |
| Power | 6.311 | 8.934 | 12.502 | 0.383 | 73.194 | 0.746 | -0.014 | Viable |  |
| **Unrestricted Models** | | | | | | | | | |
| Linear | 2.654 | 7.453 | 32.624 | 0.000447 | 88.381 | 0.398 | -0.071 | Questionable |  |
| - * BMDS recommended best fitting model - † User selected best fitting model | | | | | | | | | |

### Dataset Name: MucilAir Triton X-100, TEER (rel. vehicle)

| **Dose (µg/cm²)** | **N** | **Mean** | **Std. Dev.** | |
| --- | --- | --- | --- | --- |
| 0 | 3 | 1 | 0.127 | |
| 4.2 | 3 | 0.931 | 0.058 | |
| 9.1 | 3 | 0.759 | 0.146 | |
| 18.6 | 3 | 0.175 | 0.028 | |
| 37.8 | 3 | 0.121 | 0.041 | |
| 77.9 | 3 | 0.079 | 0.004 | |
| Test 1 Dose response: < 0.0001  Test 2 Homogeneity of Variance: < 0.0001  Test 3 Variance Model Selection: < 0.0001 | | | | |
| **MR Type** | | | Std. Dev. |  |
| **BMRF** | | | 1 |  |
| **Distribution Type** | | | Normal + Constant |  |
| **Adverse Direction** | | | Automatic |  |
| **Maximum Polynomial Degree** | | | 3 |  |
| **Confidence Level (one sided)** | | | 0.95 |  |

Maximum Likelihood Approach Model Results

| **Model** | **BMDL (µg/cm²)** | **BMD (µg/cm²)** | **BMDU (µg/cm²)** | ***P*-Value** | **AIC** | **Scaled Residual at Control** | **Scaled Residual near BMD** | **Recommendation and Notes** |
| --- | --- | --- | --- | --- | --- | --- | --- | --- |
| **Restricted Models** | | | | | | | | |
| Exponential 3 | 4.022 | 6.119 | 8.929 | 0.0184 | -26.963 | 0.261 | -0.368 | Questionable |
| Exponential 5* | 4.31 | 5.873 | 7.871 | 0.578 | -33.89 | 0.435 | -0.565 | Questionable |
| Hill | 5.518 | 6.93 | 8.389 | 0.447 | -33.376 | 0.706 | 0.082 | Questionable |
| Polynomial 2 | 14.834 | 20.989 | 35.172 | < 0.0001 | 6.632 | 1.411 | -2.857 | Questionable |
| Polynomial 3 | 14.833 | 20.992 | 35.171 | < 0.0001 | 6.632 | 1.411 | -2.857 | Questionable |
| Power | 14.833 | 20.989 | 35.171 | < 0.0001 | 6.632 | 1.411 | -2.857 | Questionable |
| **Unrestricted Models** | | | | | | | | |
| Linear | 14.833 | 20.989 | 35.172 | < 0.0001 | 6.632 | 1.411 | -2.857 | Questionable |
| - * User selected best fitting model | | | | | | | | |

### Dataset Name: MucilAir Triton X-100, AAA (rel. vehicle)

| **Dose (µg/cm²)** | **N** | **Mean** | **Std. Dev.** |
| --- | --- | --- | --- |
| 0 | 3 | 1 | 0.237 |
| 4.2 | 3 | 1.152 | 0.136 |
| 9.1 | 3 | 1.261 | 0.33 |
| 18.6 | 3 | 0.169 | 0.2 |
| 37.8 | 3 | 0.442 | 0.49 |
| 77.9 | 3 | 0.078 | 0.089 |
| Test 1 Dose response: < 0.0001  Test 2 Homogeneity of Variance: 0.0772  Test 3 Variance Model Selection: 0.0772 | | | |

| **BMR Type** | Std. Dev. |
| --- | --- |
| **BMRF** | 1 |
| **Distribution Type** | Normal + Constant |
| **Adverse Direction** | Automatic |
| **Maximum Polynomial Degree** | 3 |
| **Confidence Level (one sided)** | 0.95 |

Maximum Likelihood Approach Model Results

| **Model** | **BMDL (µg/cm²)** | **BMD (µg/cm²)** | **BMDU (µg/cm²)** | ***P*-Value** | **AIC** | **Scaled Residual at Control** | **Scaled Residual near BMD** | **Recommendation and Notes** |
| --- | --- | --- | --- | --- | --- | --- | --- | --- |
| **Restricted Models** | | | | | | | | |
| Exponential 3 | 10.205 | 16.813 | 17.794 | 0.0392 | 14.076 | -0.787 | -1.22e-5 | Questionable |
| Exponential 5*† | 9.332 | 15.26 | 17.38 | 0.155 | 11.238 | -0.901 | -0.397 | Recommended - Lowest AIC |
| Hill | 9.626 | 12.625 | 16.785 | 0.154 | 11.259 | -0.902 | 0.812 | Viable |
| Polynomial 2 | 18.661 | 27.82 | 54.163 | 0.00118 | 22.097 | -0.084 | -2.729 | Questionable |
| Polynomial 3 | 18.661 | 27.815 | 54.917 | 0.00118 | 22.097 | -0.084 | -2.729 | Questionable |
| Power | 18.661 | 27.816 | 54.141 | 0.00118 | 22.097 | -0.084 | -2.729 | Questionable |
| **Unrestricted Models** | | | | | | | | |
| Linear | 18.661 | 27.816 | 53.776 | 0.00118 | 22.097 | -0.084 | -2.729 | Questionable |
| - * BMDS recommended best fitting model - † User selected best fitting model | | | | | | | | |

### Dataset Name: MucilAir Triton X-100, CBF (rel. vehicle)

| **Dose (µg/cm²)** | **N** | **Mean** | **Std. Dev.** |
| --- | --- | --- | --- |
| 0 | 3 | 1 | 0.136 |
| 4.2 | 3 | 1.055 | 0.19 |
| 9.1 | 3 | 0.911 | 0.134 |
| 18.6 | 3 | 0.725 | 0.08 |
| 37.8 | 3 | 0.836 | 0.119 |
| Test 1 Dose response: 0.100  Test 2 Homogeneity of Variance: 0.705  Test 3 Variance Model Selection: 0.705 | | | |

| **BMR Type** | Std. Dev. |
| --- | --- |
| **BMRF** | 1 |
| **Distribution Type** | Normal + Constant |
| **Adverse Direction** | Automatic |
| **Maximum Polynomial Degree** | 3 |
| **Confidence Level (one sided)** | 0.95 |

Maximum Likelihood Approach Model Results

| **Model** | **BMDL (µg/cm²)** | **BMD (µg/cm²)** | **BMDU (µg/cm²)** | ***P*-Value** | **AIC** | **Scaled Residual at Control** | **Scaled Residual near BMD** | **Recommendation and Notes** |
| --- | --- | --- | --- | --- | --- | --- | --- | --- |
| **Restricted Models** | | | | | | | | |
| Exponential 3 | 10.654 | 21.427 | 21.871 | 0.0777 | -10.44 | 0.081 | -1.759 | Questionable |
| Exponential 5*† | 4.979 | 9.11 | 18.17 | 0.417 | -13.516 | -0.403 | -6.82e-7 | Recommended - Lowest AIC |
| Hill | 5.146 | 9.113 | 70.25 | 0.417 | -13.516 | -0.403 | 8.15e-6 | Viable |
| Polynomial 2 | 13.232 | 24.114 | 130.756 | 0.0685 | -10.155 | 0.159 | -1.878 | Questionable |
| Polynomial 3 | 13.229 | 24.081 | 130.724 | 0.0685 | -10.155 | 0.158 | -1.877 | Questionable |
| Power | 13.229 | 24.08 | 130.729 | 0.0685 | -10.155 | 0.158 | -1.877 | Questionable |
| **Unrestricted Models** | | | | | | | | |
| Linear | 13.229 | 24.08 | 130.762 | 0.0685 | -10.155 | 0.158 | -1.877 | Questionable |
| - * BMDS recommended best fitting model - † User selected best fitting model | | | | | | | | |

## MucilAir: Triton X-100, 7 d

### Dataset Name: 7d MucilAir Triton X-100, IL-6 (pg/mL*^)

*raw units selected to improve model fit, but BMDL/BMD values were highly similar using either absolute or normalized data

^very large standard deviations within dataset which decreases confidence

| **Dose (µg/cm²)** | **N** | **Mean** | **Std. Dev.** |
| --- | --- | --- | --- |
| 0 | 3 | 45.853 | 53.168 |
| 4.2 | 3 | 33.364 | 29.38 |
| 9.1 | 3 | 58.167 | 64.595 |
| 18.6 | 3 | 59.557 | 60.282 |
| Test 1 Dose response: 0.866  Test 2 Homogeneity of Variance: 0.607  Test 3 Variance Model Selection: 0.607 | | | |
| **BMR Type** | | Std. Dev. |  |
| **BMRF** | | 1 |  |
| **Distribution Type** | | Normal + Constant |  |
| **Adverse Direction** | | Automatic |  |
| **Maximum Polynomial Degree** | | 3 |  |
| **Confidence Level (one sided)** | | 0.95 |  |

Maximum Likelihood Approach Model Results

| **Model** | **BMDL (µg/cm²)** | **BMD (µg/cm²)** | **BMDU (µg/cm²)** | ***P*-Value** | **AIC** | **Scaled Residual at Control** | **Scaled Residual near BMD** | **Recommendation and Notes** |
| --- | --- | --- | --- | --- | --- | --- | --- | --- |
| **Restricted Models** | | | | | | | | |
| Exponential 3 | 13.712 | 32.42 | - | 0.840 | 133.099 | 0.161 | -0.063 | Viable |
| Exponential 5 | - | - | - | 0.727 | 132.872 | 0.246 | - | Unusable |
| Hill | - | - | - | 0.727 | 132.872 | 0.246 | - | Unusable |
| Polynomial 2 | 10.721 | 32.123 | 32.788 | 0.552 | 133.104 | 0.15 | -0.061 | Viable |
| Polynomial 3 | 10.718 | 31.368 | 32.018 | 0.551 | 133.106 | 0.14 | -0.064 | Viable |
| Power | 27.616 | 35.008 | 47.078 | 0.559 | 133.092 | 0.168 | -0.07 | Viable |
| **Unrestricted Models** | | | | | | | | |
| Linear*† | 10.724 | 41.279 | - | 0.839 | 131.102 | 0.203 | -0.043 | Recommended - Lowest AIC |
| - * BMDS recommended best fitting model - † User selected best fitting model | | | | | | | | |

### Dataset Name: 7d MucilAir Triton X-100, IL-8 (rel. vehicle^)

^very large standard deviations within dataset which decreases confidence

| **Dose (µg/cm²)** | **N** | **Mean** | **Std. Dev.** |
| --- | --- | --- | --- |
| 0 | 3 | 1 | 0.224 |
| 4.2 | 3 | 1.455 | 0.675 |
| 9.1 | 3 | 1.402 | 0.614 |
| 18.6 | 3 | 1.543 | 0.44 |
| Test 1 Dose response: 0.419  Test 2 Homogeneity of Variance: 0.329  Test 3 Variance Model Selection: 0.329 | | | |

| **BMR Type** | Std. Dev. |
| --- | --- |
| **BMRF** | 1 |
| **Distribution Type** | Normal + Non-constant |
| **Adverse Direction** | Automatic |
| **Maximum Polynomial Degree** | 3 |
| **Confidence Level (one sided)** | 0.95 |

Maximum Likelihood Approach Model Results

| **Model** | **BMDL (µg/cm²)** | **BMD (µg/cm²)** | **BMDU (µg/cm²)** | ***P*-Value** | **AIC** | **Scaled Residual at Control** | **Scaled Residual near BMD** | **Recommendation and Notes** |
| --- | --- | --- | --- | --- | --- | --- | --- | --- |
| **Restricted Models** | | | | | | | | |
| Exponential 3 | 9.962 | 19.855 | - | 0.559 | 20.601 | -0.702 | -0.192 | Viable |
| Exponential 5 | 0.109 | 3.274 | - | 0.686 | 21.601 | -6.14e-4 | 0.021 | Questionable |
| Hill | 0.004 | 4.103 | - | 0.715 | 21.57 | -0.001 | 0.109 | Questionable |
| Polynomial 2 | 8.076 | 18.895 | - | 0.582 | 20.521 | -0.637 | -0.22 | Viable |
| Polynomial 3 | 8.078 | 18.959 | - | 0.582 | 20.521 | -0.639 | -0.216 | Viable |
| Power | 8.076 | 18.89 | - | 0.582 | 20.521 | -0.637 | -0.22 | Viable |
| **Unrestricted Models** | | | | | | | | |
| Linear*† | 8.076 | 18.89 | - | 0.582 | 20.521 | -0.637 | -0.22 | Recommended - Lowest AIC |

### Dataset Name: 7d MucilAir Triton X-100, TEER (Ω ·cm2*)

*raw units selected to improve model fit, but BMDL/BMD values were highly similar using either absolute or normalized data

| **Dose (µg/cm²)** | **N** | **Mean** | **Std. Dev.** |
| --- | --- | --- | --- |
| 0 | 3 | 563.64 | 106.709 |
| 4.2 | 3 | 526.185 | 98.972 |
| 9.1 | 3 | 582.56 | 139.895 |
| 18.6 | 3 | 672.87 | 161.853 |
| 37.8 | 3 | 712.085 | 115.559 |
| 77.9 | 3 | 60.61 | 7.583 |
| Test 1 Dose response: < 0.0001  Test 2 Homogeneity of Variance: 0.0120  Test 3 Variance Model Selection: 0.956 | | | |

| **BMR Type** | Std. Dev. |
| --- | --- |
| **BMRF** | 1 |
| **Distribution Type** | Normal + Non-constant |
| **Adverse Direction** | Automatic |
| **Maximum Polynomial Degree** | 3 |
| **Confidence Level (one sided)** | 0.95 |

Maximum Likelihood Approach Model Results

| **Model** | **BMDL (µg/cm²)** | **BMD (µg/cm²)** | **BMDU (µg/cm²)** | ***P*-Value** | **AIC** | **Scaled Residual at Control** | **Scaled Residual near BMD** | **Recommendation and Notes** |
| --- | --- | --- | --- | --- | --- | --- | --- | --- |
| **Restricted Models** | | | | | | | | |
| Exponential 3 | - | 68.092 | - | 0.0283 | 221.705 | -0.736 | 0.406 | Unusable |
| Exponential 5*† | 40.02 | 67.056 | 70.154 | 0.114 | 216.813 | -0.664 | -1.70e-4 | Recommended - Lowest AIC |
| Hill | 40.933 | 59.908 | 62.784 | 0.114 | 216.814 | -0.664 | -1.59e-5 | Viable |
| Polynomial 2 | 36.67 | 42.699 | 51.682 | 0.00408 | 224.174 | -0.997 | 2.463 | Questionable |
| Polynomial 3 | 43.67 | 49.326 | 55.717 | 0.0472 | 218.483 | -0.817 | 2.006 | Questionable |
| Power | 50.37 | 71.642 | 73.356 | 0.113 | 216.827 | -0.685 | 0.002 | Viable |
| **Unrestricted Models** | | | | | | | | |
| Linear | 13,759.926 | 13,918.07 | 14,402.584 | < 0.0001 | 277.325 | 1.501 | 1.723 | Questionable |
| - * BMDS recommended best fitting model - † User selected best fitting model | | | | | | | | |

### Dataset Name: 7d MucilAir Triton X-100, TEER2 (Ω ·cm2*) [dropped highest Dose (µg/cm²) since strong evidence of a biphasic response]

| **Dose (µg/cm²)** | **N** | **Mean** | **Std. Dev.** |
| --- | --- | --- | --- |
| 0 | 3 | 563.64 | 106.709 |
| 4.2 | 3 | 526.185 | 98.972 |
| 9.1 | 3 | 582.56 | 139.895 |
| 18.6 | 3 | 672.87 | 161.853 |
| 37.8 | 3 | 712.085 | 115.559 |
| Test 1 Dose response: 0.578  Test 2 Homogeneity of Variance: 0.909  Test 3 Variance Model Selection: 0.883 | | | |

| **BMR Type** | Std. Dev. |
| --- | --- |
| **BMRF** | 1 |
| **Distribution Type** | Normal + Non-constant |
| **Adverse Direction** | Automatic |
| **Maximum Polynomial Degree** | 3 |
| **Confidence Level (one sided)** | 0.95 |

Maximum Likelihood Approach Model Results

| **Model** | **BMDL (µg/cm²)** | **BMD (µg/cm²)** | **BMDU (µg/cm²)** | ***P*-Value** | **AIC** | **Scaled Residual at Control** | **Scaled Residual near BMD** | **Recommendation and Notes** |
| --- | --- | --- | --- | --- | --- | --- | --- | --- |
| **Restricted Models** | | | | | | | | |
| Exponential 3 | 11.031 | 22.647 | 94.574 | 0.718 | 190.746 | 0.271 | 0.683 | Viable |
| Exponential 5 | 4.786 | 13.854 | - | 0.550 | 193.756 | 0.373 | -0.149 | Viable |
| Hill | 5.568 | 12.499 | - | 0.579 | 193.706 | 0.387 | -0.034 | Viable |
| Polynomial 2 | 9.416 | 20.726 | 91.352 | 0.739 | 190.657 | 0.341 | 0.616 | Viable |
| Polynomial 3*† | 9.324 | 20.726 | - | 0.739 | 190.657 | 0.341 | 0.616 | Recommended - Lowest AIC |
| Power | 9.314 | 20.726 | 95.879 | 0.739 | 190.657 | 0.341 | 0.616 | Viable |
| **Unrestricted Models** | | | | | | | | |
| Linear | 9.314 | 20.726 | 95.892 | 0.739 | 190.657 | 0.341 | 0.616 | Viable |
| - * BMDS recommended best fitting model - † User selected best fitting model | | | | | | | | |

### Dataset Name: 7d MucilAir Triton X-100, Viability (rel. vehicle)

| **Dose (µg/cm²)** | **N** | **Mean** | **Std. Dev.** |
| --- | --- | --- | --- |
| 0 | 3 | 1 | 0.132 |
| 4.2 | 3 | 1.202 | 0.176 |
| 9.1 | 3 | 1.165 | 0.115 |
| 18.6 | 3 | 1.059 | 0.274 |
| 37.8 | 3 | 0.312 | 0.044 |
| 77.9 | 3 | 0.199 | 0.122 |
| Test 1 Dose response: < 0.0001  Test 2 Homogeneity of Variance: 0.122  Test 3 Variance Model Selection: 0.122 | | | |

| **BMR Type** | Std. Dev. |
| --- | --- |
| **BMRF** | 1 |
| **Distribution Type** | Normal + Non-constant |
| **Adverse Direction** | Automatic |
| **Maximum Polynomial Degree** | 3 |
| **Confidence Level (one sided)** | 0.95 |

Maximum Likelihood Approach Model Results

| **Model** | **BMDL (µg/cm²)** | **BMD (µg/cm²)** | **BMDU (µg/cm²)** | ***P*-Value** | **AIC** | **Scaled Residual at Control** | **Scaled Residual near BMD** | **Recommendation and Notes** |
| --- | --- | --- | --- | --- | --- | --- | --- | --- |
| **Restricted Models** | | | | | | | | |
| Exponential 3 | 15.761 | 23.753 | 35.039 | 0.0346 | -5.566 | -1.271 | -0.045 | Questionable |
| Exponential 5 | 15.941 | 22.542 | 34.22 | 0.158 | -8.504 | -1.46 | -0.038 | Viable |
| Hill*† | 16.458 | 21.518 | 32.447 | 0.159 | -8.523 | -1.464 | -0.012 | Recommended - Lowest AIC |
| Polynomial 2 | 11.946 | 16.358 | 28.679 | 0.000695 | 3.074 | -1.229 | 1.198 | Questionable |
| Polynomial 3 | 11.946 | 16.361 | 28.678 | 0.000695 | 3.074 | -1.229 | 1.198 | Questionable |
| Power | 11.946 | 16.36 | 31.463 | 0.000695 | 3.074 | -1.229 | 1.198 | Questionable |
| **Unrestricted Models** | | | | | | | | |
| Linear | 11.946 | 16.36 | 25.351 | 0.000695 | 3.074 | -1.229 | 1.198 | Questionable |

### Dataset Name: 7d MucilAir Triton X-100, AAA (rel. vehicle)

| **Dose (µg/cm²)** | **N** | **Mean** | **Std. Dev.** |
| --- | --- | --- | --- |
| 0 | 3 | 1 | 0.001 |
| 4.2 | 3 | 0.997 | 0.002 |
| 9.1 | 3 | 0.97 | 0.028 |
| 18.6 | 3 | 0.644 | 0.459 |
| 37.8 | 3 | 0.014 | 0.008 |
| 77.9 | 3 | 0.014 | 0.006 |
| Test 1 Dose response: 0  Test 2 Homogeneity of Variance: 0  Test 3 Variance Model Selection: 0 | | | |

| **BMR Type** | Std. Dev. |
| --- | --- |
| **BMRF** | 1 |
| **Distribution Type** | Normal + Constant |
| **Adverse Direction** | Automatic |
| **Maximum Polynomial Degree** | 3 |
| **Confidence Level (one sided)** | 0.95 |

Maximum Likelihood Approach Model Results

| **Model** | **BMDL (µg/cm²)** | **BMD (µg/cm²)** | **BMDU (µg/cm²)** | ***P*-Value** | **AIC** | **Scaled Residual at Control** | **Scaled Residual near BMD** | **Recommendation and Notes** |  |
| --- | --- | --- | --- | --- | --- | --- | --- | --- | --- |
| **Restricted Models** | | | | | | | | | |
| Exponential 3* | 8.905 | 13.932 | 18.692 | 1.00 | -8.389 | -0.028 | -0.018 | Questionable |  |
| Exponential 5 | 8.937 | 14.384 | 18.647 | 1.00 | -6.426 | 0.009 | 5.01e-4 | Questionable |  |
| Hill | 10.601 | 16.045 | 18.603 | 0.969 | -6.363 | 0.112 | 0.005 | Questionable |  |
| Polynomial 2 | 12.323 | 16.944 | 26.705 | 0.00178 | 6.76 | 0.243 | -0.351 | Questionable |  |
| Polynomial 3 | 12.322 | 16.942 | 26.704 | 0.00178 | 6.76 | 0.243 | -0.351 | Questionable |  |
| Power | 12.323 | 16.943 | 27.504 | 0.00178 | 6.76 | 0.243 | -0.351 | Questionable |  |
| **Unrestricted Models** | | | | | | | | | |
| Linear | 12.323 | 16.943 | 26.493 | 0.00178 | 6.76 | 0.243 | -0.351 | Questionable |  |
| - * User selected best fitting model, Raw and normalized data both support selection of the exp3 model (constant variance). Both datasets support a BMD/BMDL of 13.1/8.9 for the exp 3 model, which is vary close to the NOAEC for this dataset of 9.1 | | | | | | | | | |

### Dataset Name: 7d MucilAir Triton X-100, CBF (rel. vehicle^)

^very large standard deviations within dataset which decreases confidence.

| **Dose (µg/cm²)** | **N** | **Mean** | **Std. Dev.** |
| --- | --- | --- | --- |
| 0 | 3 | 1 | 0.189 |
| 4.2 | 3 | 0.957 | 0.08 |
| 9.1 | 3 | 0.895 | 0.164 |
| 18.6 | 3 | 0.958 | 0.08 |
| Test 1 Dose response: 0.574  Test 2 Homogeneity of Variance: 0.321  Test 3 Variance Model Selection: 0.321 | | | |

| **BMR Type** | Std. Dev. |
| --- | --- |
| **BMRF** | 1 |
| **Distribution Type** | Normal + Constant |
| **Adverse Direction** | Automatic |
| **Maximum Polynomial Degree** | 3 |
| **Confidence Level (one sided)** | 0.95 |

Maximum Likelihood Approach Model Results

| **Model** | **BMDL (µg/cm²)** | **BMD (µg/cm²)** | **BMDU (µg/cm²)** | ***P*-Value** | **AIC** | **Scaled Residual at Control** | **Scaled Residual near BMD** | **Recommendation and Notes** |  |
| --- | --- | --- | --- | --- | --- | --- | --- | --- | --- |
| **Restricted Models** | | | | | | | | | |
| Exponential 3 | 18.493 | 1,533.879 | 20,822.401 | 0.530 | -9.193 | 0.696 | 0.081 | Questionable |  |
| Exponential 5 | 18.602 | 1,832.23 | 26,269.774 | - | -7.193 | 0.696 | 0.081 | Questionable |  |
| Hill | - | - | - | 0.532 | -11.202 | 0.723 | - | Unusable |  |
| Polynomial 2 | 11.541 | 57.043 | - | 0.580 | -11.375 | 0.459 | 0.404 | Viable |  |
| Polynomial 3 | 11.54 | 57.387 | - | 0.580 | -11.375 | 0.461 | 0.402 | Viable |  |
| Power*† | 11.541 | 56.025 | 19,064.672 | 0.580 | -11.375 | 0.455 | 0.41 | Recommended - Lowest AIC |  |
| **Unrestricted Models** | | | | | | | | | |
| Linear | 11.54 | 56.025 | - | 0.580 | -11.375 | 0.455 | 0.41 | Viable |  |
| - * BMDS recommended best fitting model - † User selected best fitting model | | | | | | | | | |
